# Supplementary material for: In vivo interrogation of regulatory genomes reveals extensive quasi-insufficiency in cancer evolution
Source: Cell Genom. 2023 Mar 8;3(3):100276. doi: 10.1016/j.xgen.2023.100276 (PMC10025556; doi:10.1016/j.xgen.2023.100276)
Supplement: Document S1. Figures S1–S16 [file mmc1.pdf]

Cell Genomics, Volume *3*

Supplemental information

In vivo interrogation of regulatory genomes reveals extensive quasi-insufficiency in cancer evolution

Anja Fischer, Robert Lersch, Niklas de Andrade Krätzig, Alexander Strong, Mathias J. Friedrich, Julia Weber, Thomas Engleitner, Rupert Öllinger, Hsi-Yu Yen, Ursula Kohlhofer, Irene Gonzalez-Menendez, David Sailer, Liz Kogan, Mari Lahnalampi, Saara Laukkanen, Thorsten Kaltenbacher, Christine Klement, Majdaddin Rezaei, Tim Ammon, Juan J. Montero, Günter Schneider, Julia Mayerle, Mathias Heikenwälder, Marc Schmidt-Supprian, Leticia Quintanilla-Martinez, Katja Steiger, Pentao Liu, Juan Cadiñanos, George S. Vassiliou, Dieter Saur, Olli Lohi, Merja Heinäniemi, Nathalie Conte, Allan Bradley, Lena Rad, and Roland Rad

Figure S1

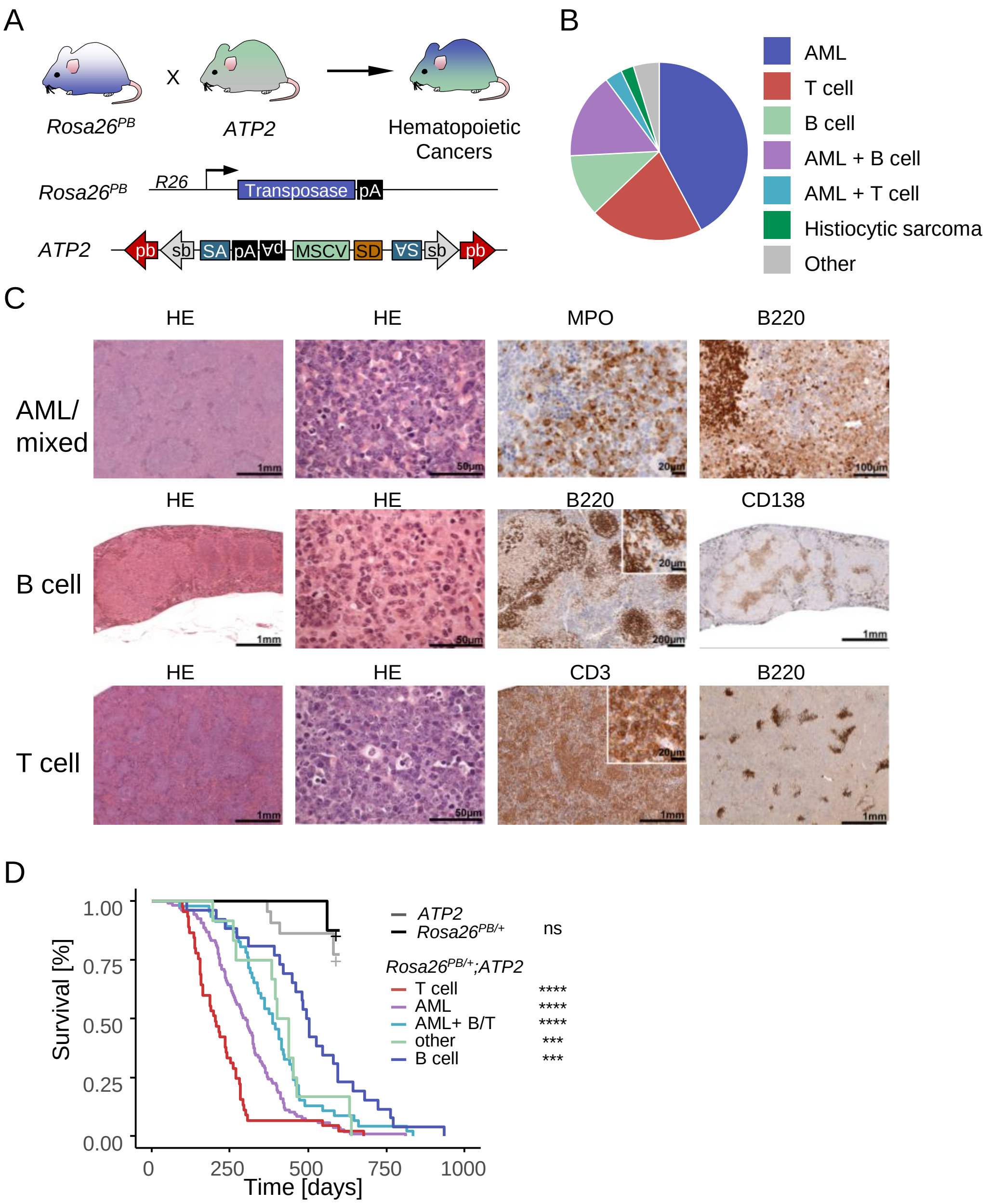

Supplementary Figure 1. Histopathological characterization of the Rosa26PB;ATP2 screening cohort. A, Mating scheme for *PiggyBac* screening using ATP2 mouse lines. In *Rosa26PB;ATP2* double positive mice transposon mobilization is active and mice develop hematopoietic cancers. Structure of Rosa26PB allele and ATP2 transposons used to generate ATP2 transgenic mice. **B,** Overview of histopathological diagnoses of hematopoietic malignancies in *Rosa26PB;ATP2* mice. Spectrum of 256 hematopoietic tumors diagnosed in the ATP2 screening cohort. Samples classified as 'other' represent samples where (i) no diagnosis was possible due to tissue lysis, (ii) the diagnosis of a benign immunological phenomenon such as spleen hyperplasia or (iii) the diagnosis of a lymphoblastic proliferation not further characterized. **C,** Representative images of tumors from all major subgroups. The first row shows an acute leukemia (AML) case with MPO expression. Additionally, the shown AML case expresses the B cell marker B220. The second row represents a diffuse large B cell lymphoma (DLBCL) characterized by B220 expression. The additional expression of CD138 led to the classification of DLBCL with plasmacytoid differentiation. The third row shows a T cell (lymphoblastic) lymphoma characterized by strong CD3 expression and the absence of B220 expression. Magnifications: first column: 25x; second: 630x; third: MPO 400x, B220 50x (insert 400x), CD3 25x (insert 400x); fourth: B220 200x, CD138 25x, B220 25x. **D,** Kaplan-Meier plot showing survival of *Rosa26PB/+;ATP2* and single-allele control mice. Survival of Rosa26PB;ATP2 mice with different types of hematopoietic tumors (AML n = 107, AML + B/T cell n = 46, B cell n = 26, T cell n = 45, other n = 12). All comparisons between the single-allele ATP2 control mice and the tumor cohorts using the Log-Rank test are shown. Benjamini-Hochberg (FDR) was used to adjust for multiple testing. *P < 0.05, **P < 0.01, ***P < 0.001, ****P < 0.0001. Related to Table S1.

Figure S2

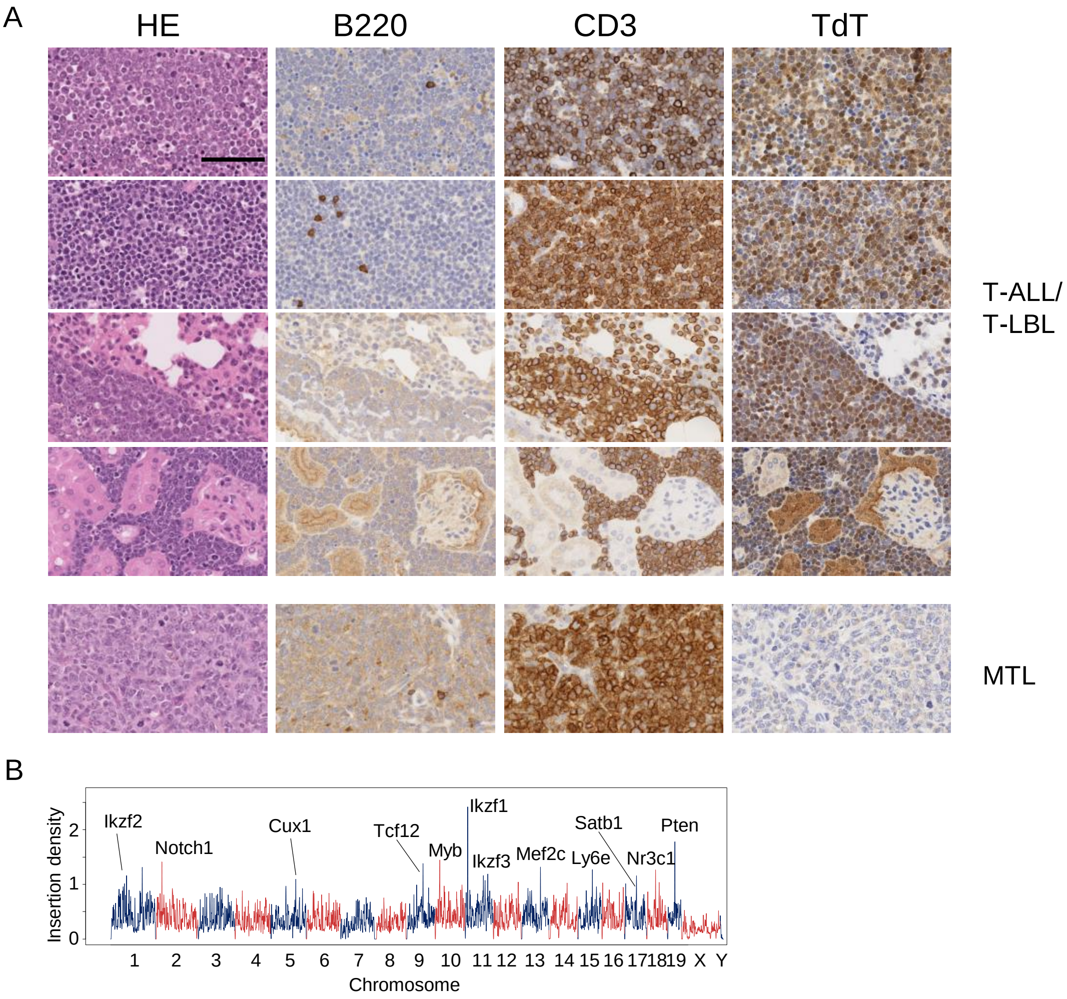

Supplementary Figure 2. Histopathological classification of Rosa26$^{PB/+}$;ATP2 transposon mice with T cell malignancies. A, Immunohistochemical characterization of mouse T cell leukemias/lymphomas. Microscopic images of five representative cases, 4 classified as T-ALL/T-LBL and one as mature T cell lymphoma (MTL). T-ALL/T-LBL cases were characterized by medium sized cells, round to ovoid nuclei and a scant cytoplasm. The nuclei had fine or dispersed (salt and pepper) chromatin and usually one central nucleolus. MTLs were negative for Tdt and showed a heterogeneous morphology ranging from cases with small cell size and inconspicuous nuclei to ones with medium cell size with irregular nuclei. Tumors are negative for B220/CD45R (B cell marker) and show strong expression of CD3 (T cell marker). CD3 expression was found on the membrane, but also in the cytoplasm. Cytoplasmic expression of Tdt (early T cell marker) was used to differentiate between T-ALL/T-LBL and mature T cell lymphomas (MTL). T-ALLs show infiltration in lung (third panel) and kidney (fourth panel). Tumors were classified according to the Bethesda proposals for classification of lymphoid neoplasms in mice (Morse et al., 2002). Scale bar, 50 µm. **B,** Genome-wide representation of transposon insertion density in T-ALLs (pooled data from 51 mice). Selected common insertion site target genes are indicated. Colors are used to distinguish chromosomes. Related to Table S1.

Figure S3

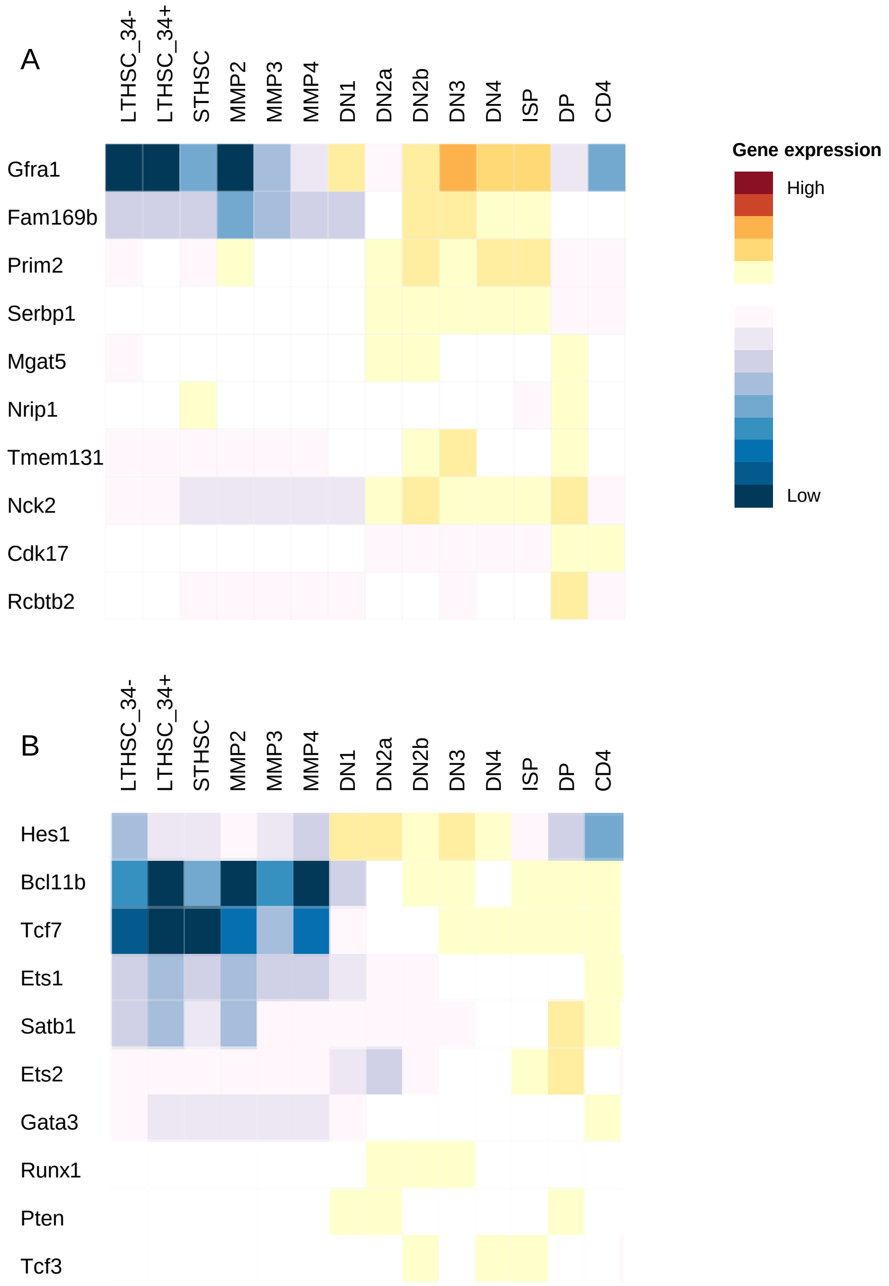

Supplementary Figure 3. Expression of candidate genes in T cell development. A, Heatmap showing expression of 10 candidate CIS genes with differential expression during murine T cell development. An increased expression level was observed in T cell precursor stages (*Prim2, Serbp1, Nck2*) or in later stages (*Nrip1, Cdk17, Rcbtb2*). **B,** Heatmap showing expression of known T cell developmental genes showing an increased expression level in T cell precursor stages (*Tcf7, Runx1*) or in later stages (*Satb1, Gata3, Ets2*). Mean-normalized heatmaps were created using the immunological genome project website (http://rstats.immgen.org/MyGeneSet_New/index.html). Related to Figure 1, Table S2/3.

Figure S4

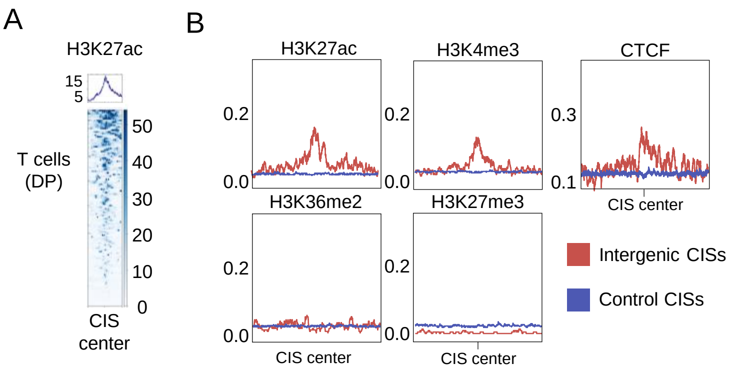

Supplementary Figure 4: Characteristics of intergenic insertions. A). Profile heatmap plot showing overlap of non-coding CIS regions with H3K27ac peaks in T cells (DP stage). **B)** Footprint plots of intergenic CISs. Enrichment of chromatin features was assessed in intergenic CISs (n = 227) from all T-ALL samples. Input data of thymus tissue (CTCF) and the EL4 cell line was used. The background line represents a set of control CISs with similar characteristics (selection of control CISs is in detail described in the Methods part). Related to Figure 1.

Figure S5

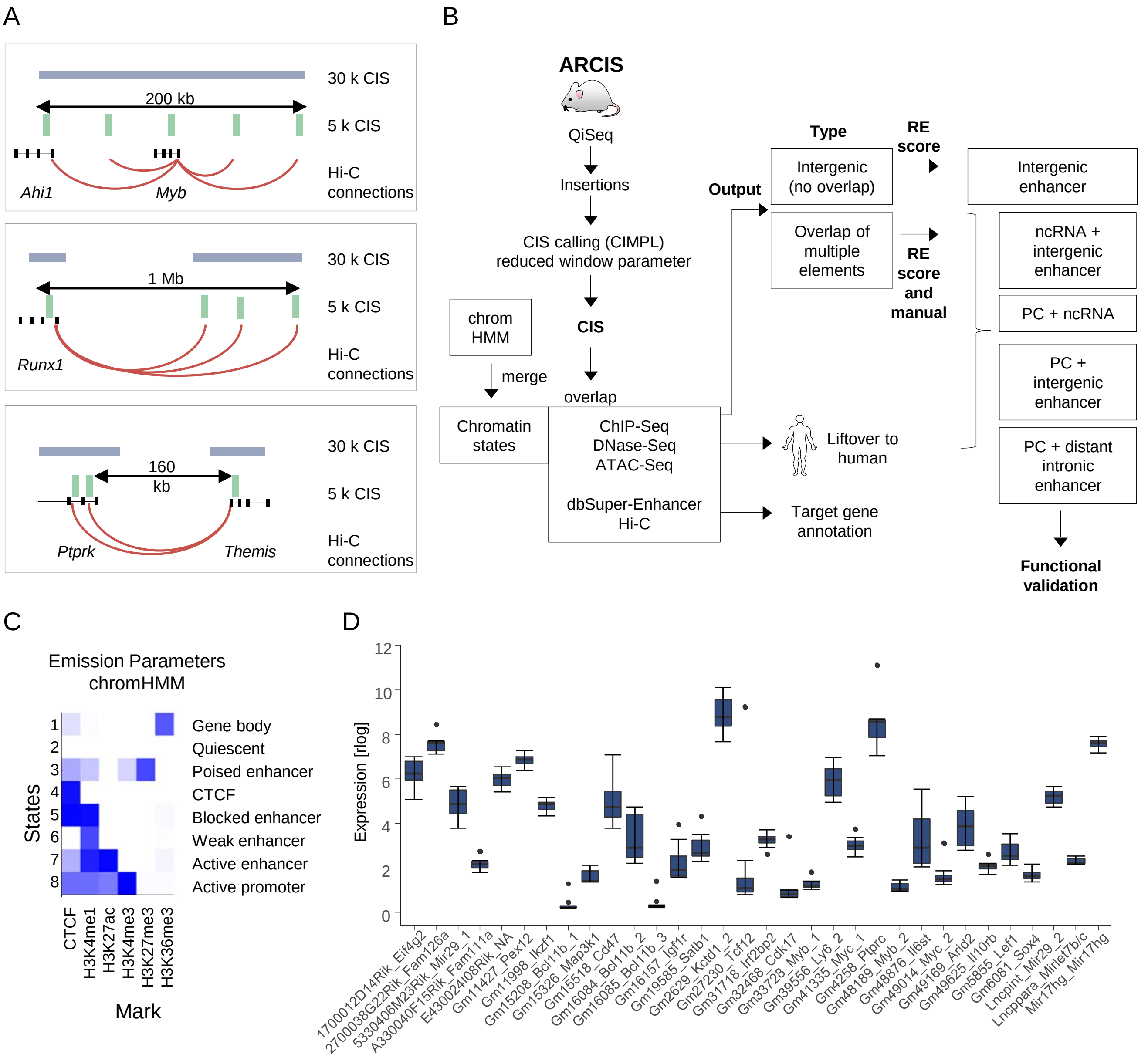

Supplementary Figure 5. The ARCIS annotation framework. A, Influence of size parameter change on common insertion sites (CIS). The effect of changing the scale parameter on CIS size using CIMPL. Exemplary loci of *Myb, Runx1* and *Themis* are shown. Blue bars represent CIS regions identified with a scale parameter of 30 k, green bars show CIS identified with a 5 k scale parameter. Relevant Hi-C connections from Hu et al. are schematically shown in red (GSE79422). To identify putative regulatory elements the desired (biologically relevant) scale needs to be adjusted by choosing a smaller window size. **B,** Schematic representation of the ARCIS framework to annotate putative regulatory elements in CIS regions. Intergenic CISs with a high "regulatory score" can directly be defined as intergenic enhancers, whilst CIS overlapping with multiple elements need further manual annotation. **C,** Emission parameters of the chromHMM model used as ARCIS input. ChromHMM was performed on thymus specific ChIP-Seq datasets (ENCODE). The 8 output states were defined as shown based on a combination of ChIP-Seq signals. **D,** rlog transformed expression values of non-coding transcripts identified by ARCIS in different stages of T cell development (HSC-DP, GSE79422). The CIS non-coding transcript and the PC gene located in close proximity are annotated. ARCIS identified 54 non-coding RNAs, 44 were annotated in the dataset and 32 were found expressed (73%). In total, the dataset includes 12,170 lncRNAs and miRNAs, of which only 1,925 are expressed (16%) with a median rlog expression level of 2,8. Related to Figure 2, Table S4/5.

Figure S6

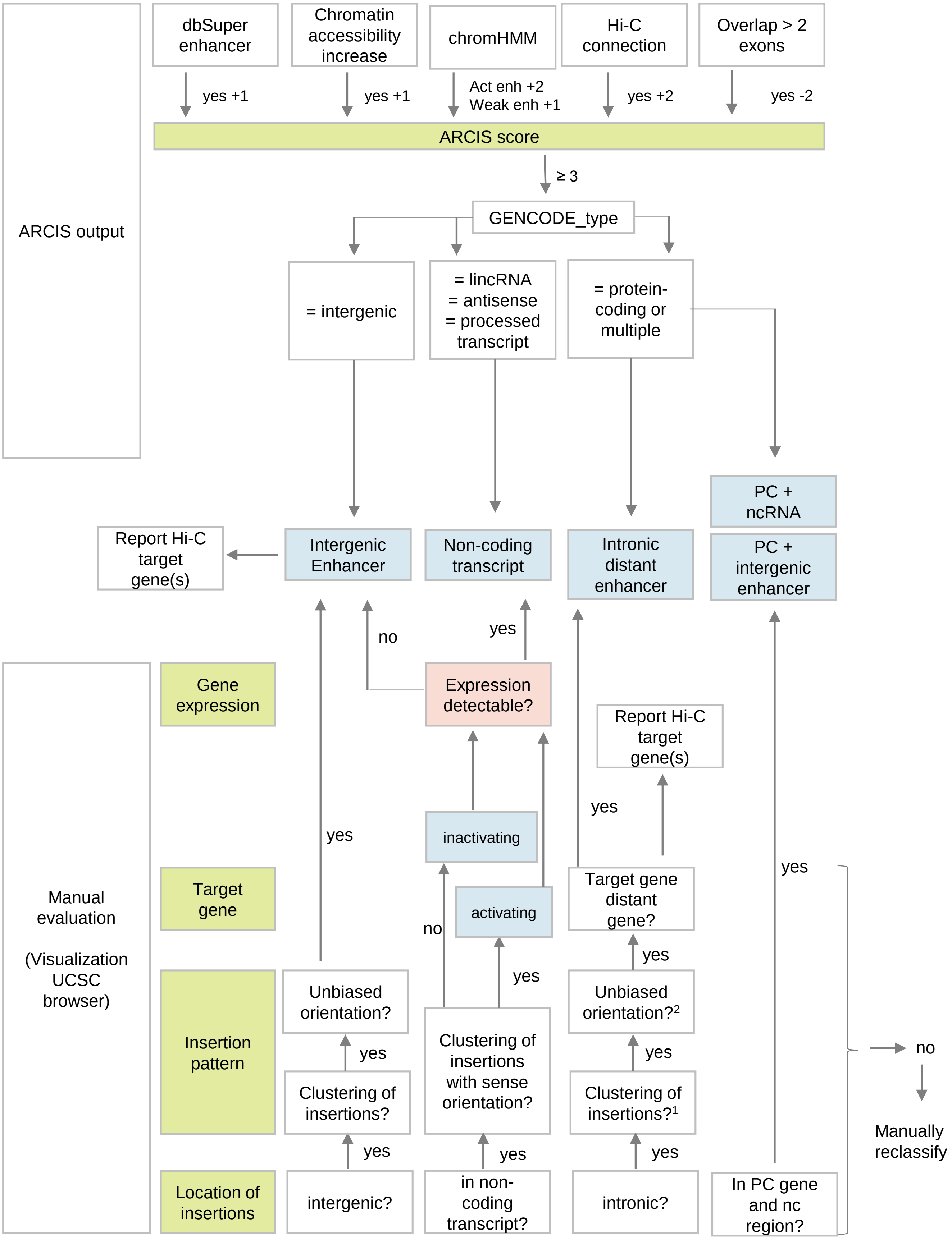

[1] no: inactivated PC gene
[2] no: activated PC gene

Supplementary Figure 6. Identification of putative regulatory regions based on ARCIS and manual evaluation. ARCIS calculates a score for each CIS region. The score is based on selected elements: overlap with an annotated super-enhancer (dbSuper) or increasing chromatin accessibility in T cell development (from Hu et al) was scored with +1, a weak and active enhancer (chromHMM) was scored with +1 and +2, respectively, any reported Hi-C connection was scored with +2. To avoid that inactivated PC genes are reported as intronic enhancer, the overlap with more than two exons was penalized with -2. The sum represents the final ARCIS score. If the ARCIS score is greater than or equal to 3, the CIS is putatively harboring a regulatory region. CIS can be ranked based on the score. To report the type of the element, the GENCODE M24 annotation type is used. According to the type, the CIS gets annotated as intergenic enhancer, as non-coding transcript, which is potentially overlapping with an enhancer or as protein-coding transcript, which might additionally harbor an intragenic or a close intergenic RE. The category 'non-coding' is defined by the overlap with an annotated nPC transcript, but does not exclude the presence of an additional enhancer element. In contrast, the annotation as enhancer element cannot exclude the presence of so far not annotated nPC transcripts. Assessing the expression of identified nPC-transcripts as shown in Supplementary Figure 5d represents further implication for the nPC transcript to play a functional role (also explained below).
The manual evaluation was based on the visualization of different datasets (Supplementary Table 5) in the UCSC genome browser. Here, we inspected the exact position of the insertions, their orientation and clustering within the CIS. In a first step we assessed whether or not the majority of the insertions overlaps with an intergenic/intronic region, non-coding transcript or with multiple types. For intergenic enhancers, we next investigated the clustering of the insertions, a characteristic for insertion peaks in REs. To exclude that this insertion peak is activating a transcript, we assessed the orientation of the insertions. If the orientation is unbiased, it is highly unlikely that these insertions activate a transcript. If any of these steps leads to a 'no' answer meaning that either insertions are not clustered or the orientation is biased, the CIS needs to be manually reclassified to the better matching category. If all steps were answered with 'yes', the target gene of the RE is reported based on the visible Hi-C connection. An optional step (red color) includes the investigation if the reported target gene is expressed in T cell development (based on RNAseq data from Hu et al.). A target gene expressed at any stage in T cell development, represents a highly interesting candidate. For intronic REs, the steps are comparable. If insertions are not clustered, the PC gene most likely gets inactivated by the transposon insertions, classifying this CIS as PC (marked with 1). If the insertions are clustered, but show strong orientation bias, the transposon might activate the PC gene (based on availability of a transcript with an ATG in exon 2 or higher). These CISs were classified as activating PC (marked with 2). If all steps were answered with 'yes', it needs to be assessed if the target gene is a distant gene (not connected to own promoter). If so, the target gene is reported. An optional step (red color) includes the investigation if the CIS gene (not the target gene) is expressed in T cell development. For genes not expressed, an inactivating function of the transposon insertions is highly unlikely, increasing the confidence to classify this CIS as intronic regulatory element. For non-coding transcripts, we differentiate into potentially activating or rather inactivating patterns based on insertion clustering and orientation. An optional step (red color) includes the investigation if the non-coding transcript is expressed in T cell development. Of note, nPC transcripts are expressed at low levels compared to PC genes. Identifying a transcript with detectable expression therefore increases the confidence that the nPC transcript is relevant for T cell biology. If insertions overlap with multiple transcripts (PC and nPC) or with a PC transcript and the intergenic area, these CISs were classified as 'PC + ncRNA' or 'PC + intergenic enhancer', respectively. Ambiguity in the ARCIS output often cannot be resolved by manual annotation. Here, functional validation is necessary.
PC, protein-coding; nPC, non-protein-coding; CIS, common insertion site.
Related to Figure 2, Table S7/8.

Figure S7

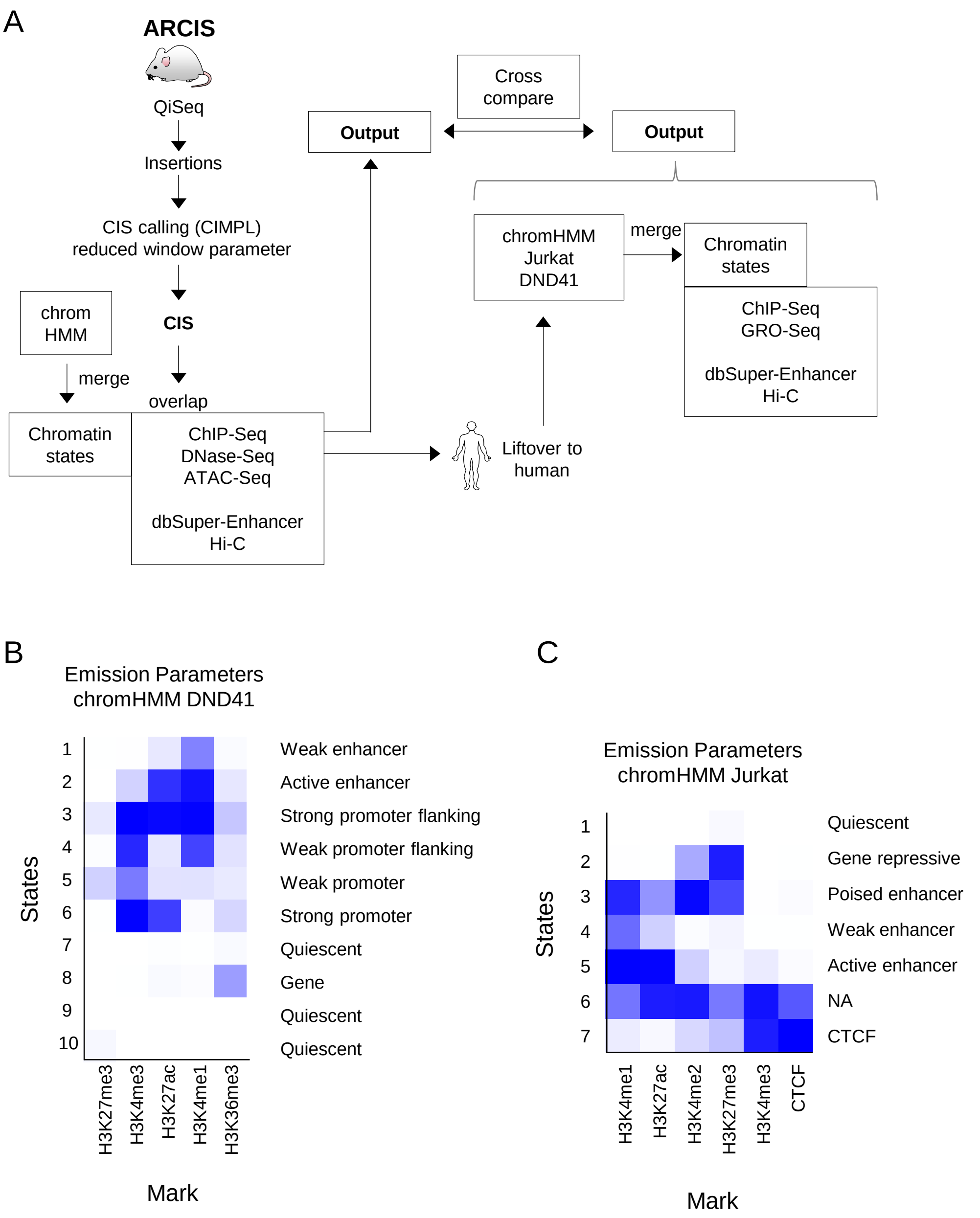

Supplementary Figure 7. Human ARCIS. **A,** Scheme showing workflow for the annotation of CISs after lift-over to the human genome. ChromHMM was performed for the T-ALL cell lines Jurkat and DND41 and publicly available ChIP-Seq, dbSuper-Enhancer and HiC data was used for annotation. Additionally, GRO-Seq data of the Jurkat and 2 T-ALL patients generated in this study was integrated. The output of the human ARCIS was cross-compared to the murine output (results shown in Figure 2d). **B,** ChromHMM Emission parameters and state annotation for the T-ALL cell line DND41. **C,** ChromHMM Emission parameters and state annotation for the T-ALL cell line Jurkat. Active and weak enhancer regions were used for CIS annotation. Related to Figure 2, Table S6/9.

Figure S8

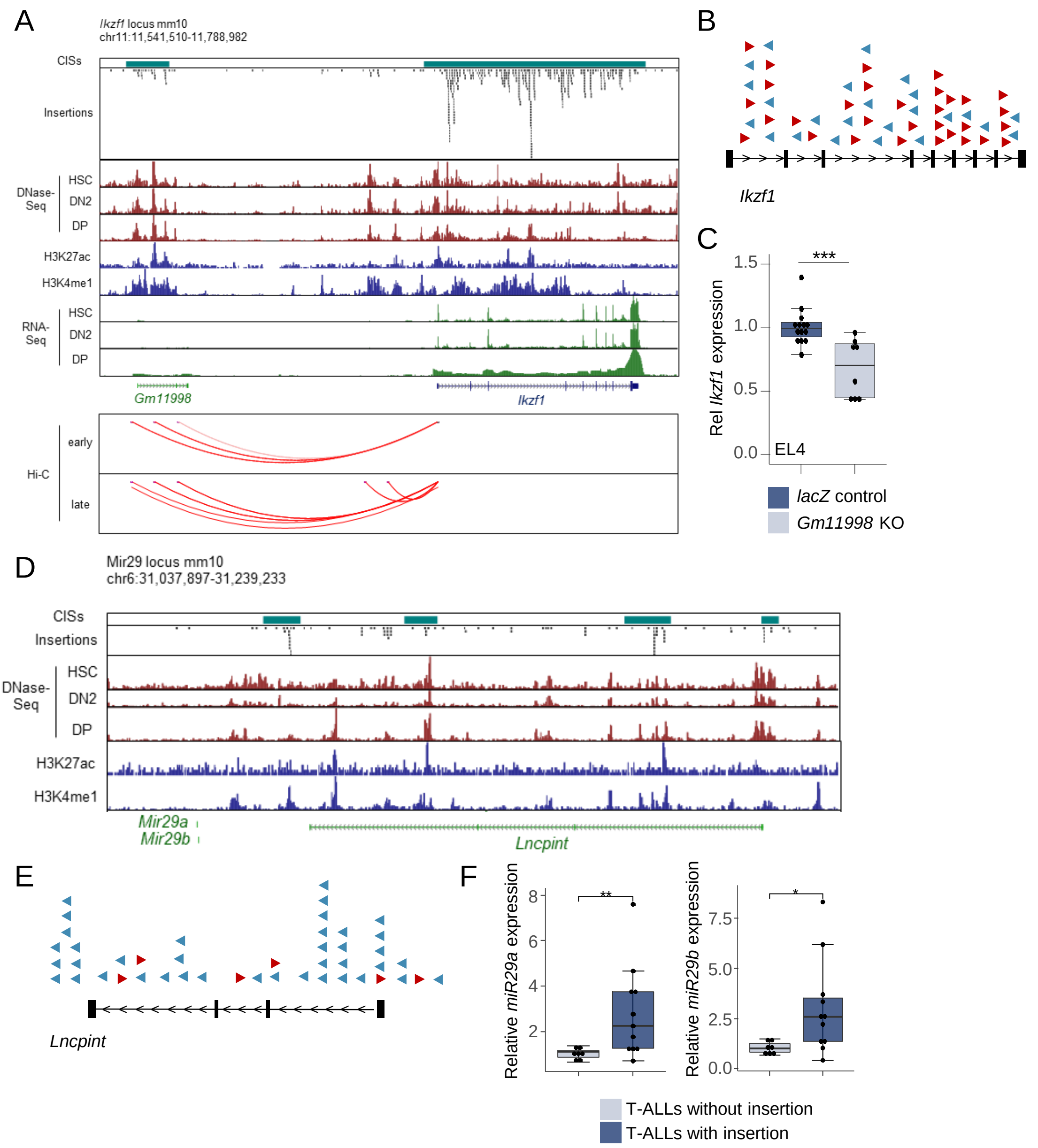

Supplementary Figure 8. Functional validation of non-coding CISs. **A,** Insertions and CISs in the murine *Ikzf1* gene locus. H3K27ac and H3K4me1 tracks from double-positive T cells, as well as DNase-Seq and Hi-C data from different stages of T cell evolution are shown below (early: HSC-DN2a, late: DN2b-DN3) (publicly available data as listed in Supplementary Table 5). An intergenic CIS region overlapping *Gm11998 also* overlaps with active chromatin and shows a Hi-C link to the *Ikzf1* promoter. **B,** Insertion pattern in *Gm11998*. Sense- and antisense-oriented insertions are depicted in different colors. **C,** *Ikzf1* expression in clones with CRISPR/Cas9-based knockout of the *Gm11998* lincRNA in the T cell lymphoblastic EL4 cell line (KO n = 8, 0/8 homozygous, ctrl n = 14). A region of ~18 kb was knocked out and electroporated cells were single cell sorted. Each dot represents relative *Ikzf1* gene expression in a single cell-derived clone normalized to *Gapdh* expression. **D,** Murine *Lncpint/miR29* gene locus showing several CISs with insertion orientations in sense direction (promoter directed to miR29a/b). **E,** Insertions pattern in the *Lncpint/miR29* locus. Sense- and antisense-oriented insertions are depicted in different colors. **D+E)** We observed four CISs in a 150 kb region upstream of the microRNA mir29a/b, of which three are overlapping with the lncRNA Lncpint. In all CISs, transposon insertions were sense-oriented, reflecting strong selective pressure for effects mediated by the transposon’s unidirectional MSCV promoter. The pattern and location of all CISs is compatible with effects supporting over-expression of mir29a, whilst overexpression of full-length Lncpint is only supported by one of the four CISs. We therefore hypothesized that miR29a/b is the target of these CISs. **F,** By performing a microRNA specific qPCR, we indeed found increased mir29a/b expression in samples with related *PiggyBac* insertions as compared to samples without insertions. Whilst such pronounced effects can be readily detected even in bulk tissue, we noted that this type of activating CIS driven by the transposon’s strong MSCV promoter is rare in the non-protein coding genome. *miR29a/b* expression in clones with and without sense-directed insertion in the *Lncpint* locus (with insertion n = 11, without insertion n = 7). Specific Taqman assays were used for quantification by qPCR. Expression was normalized to miR16. *P < 0.05, **P < 0.01, ***P < 0.001, Wilcoxon test. CIS, common insertion site; HSC, hematopoietic stem cells; DN2, double negative stage 2; DP, double positive stage; Rel, relative. Related to Figure 3.

Figure S9

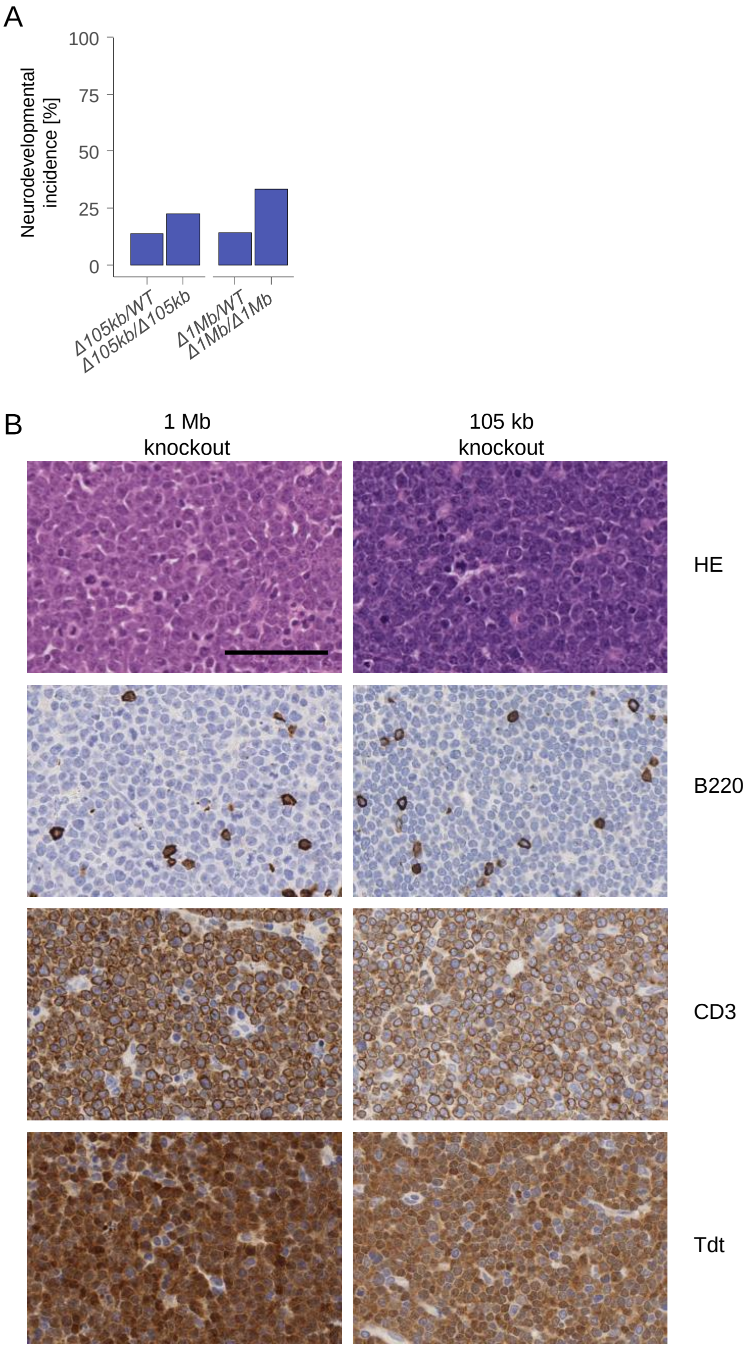

Supplementary Figure 9. Survival, phenotype and histopathological classification of intergenic knockout mice. **A,** Incidence of neurodevelopmental phenotype in all cohorts. Neurodevelopmental phenotypes include extremely small mice, mice with tremor-like symptoms or convulsions. **B,** Immunohistochemical characterization of mouse T cell leukemias/lymphomas. Microscopic images of a representative case of each cohort classified as T-ALL/T-LBL. Tumors are negative for B220 (B cell marker), show strong expression of CD3 (T cell marker) and Tdt (early T cell marker). Scale bar: 50 µm. Related to Figure 4, Table S12.

Figure S10

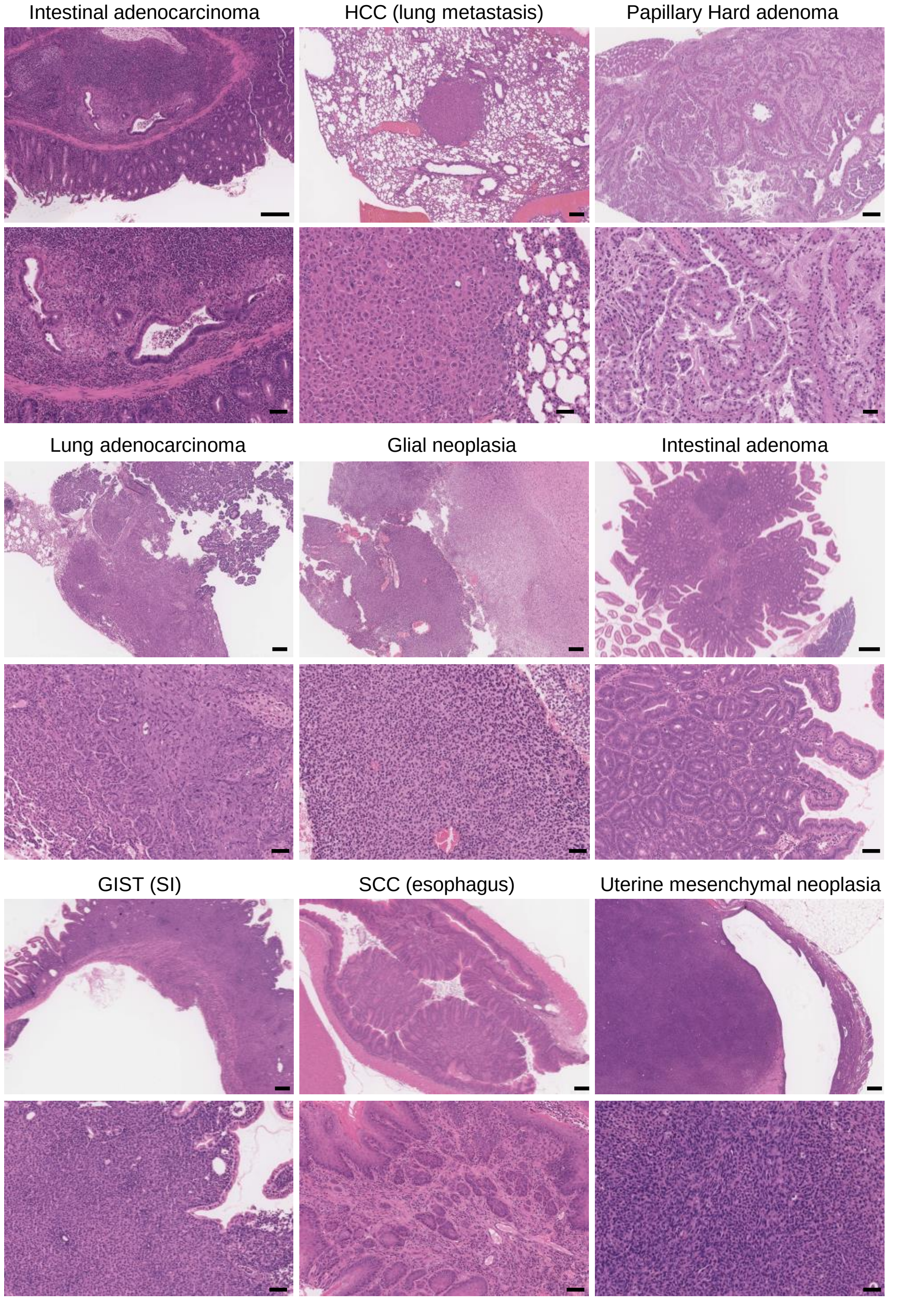

Supplementary Figure 10. Histopathological classification of solid tumors developed in intergenic knockout mice. Microscopic images show hematoxylin and eosin stainings of representative solid tumor cases from Δ105 kb and Δ1 Mb intergenic knockout mice. Only the lung adenocarcinoma was found in mice with the Δ1 Mb knockout, other solid tumors are from the Δ105 kb cohort. Two different magnifications are shown for each case. HCC, Hepatocellular carcinoma; Hard, Harderian gland; SCC, Squamous cell carcinoma; GIST, Gastrointestinal stromal tumor; SI, small intestine. Scale bars: upper panel 200 µm, lower panel 50 µm. Related to Figure 4, Table S12.

Figure S11

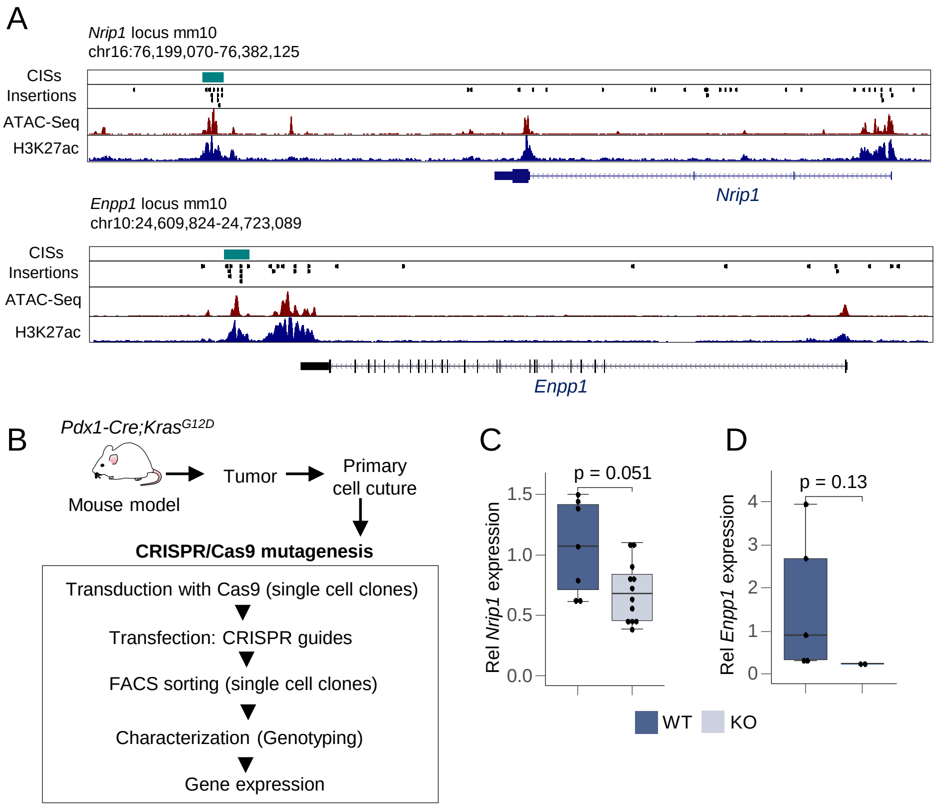

Supplementary Figure 11. Functional validation of regulatory CISs in pancreatic cancer. **A,** Insertions and CISs in the murine *Nrip1* (upper panel) and *Enpp1* (lower panel) gene locus. ATAC-Seq and H3K27ac tracks from a murine PDAC cell line are shown. **B,** Scheme showing functional validation workflow. Primary pancreatic ductal adenocarcinoma cell lines (PDAC) were isolated from mice as described in Müller et al., 2018. Stable Cas9 expressing cells were established by transducing a Cas9 expressing vector and single cell generation. Cas9-clones were further transfected with guide RNAs targeting the intergenic region of interest. As this single guide vector contains a GFP, cells were single-cell FACS sorted and expanded. Genetic knockout was characterized by PCR genotyping. Heterozygous and homozygous knockout clones were pooled (KO) and compared to wildtype (WT) clones in target gene expression analysis. **C,** *Nrip1* expression in clones with CRISPR/Cas9-based knockout of the intergenic region ~145 kb downstream of *Nrip1* in C2a-derived murine PDAC cell line (KO n = 12, 5/12 homozygous, WT n = 7). A region of ~4.5 kb was knocked out and transfected cells were single cell sorted. Each dot represents relative *Nrip1* gene expression in a single cell-derived clone normalized to *Gapdh* expression. p value is shown as a result of a t-test. **D,** *Enpp1* expression in clones with CRISPR/Cas9-based knockout of the intergenic region 80 kb downstream of *Enpp1* in C1-derived murine PDAC cell line (KO n = 2, 0/2 homozygous, WT n = 5). A region of ~3.5 kb was knocked out and transfected cells were single cell sorted. Each dot represents relative *Enpp1* gene expression in a single cell-derived clone normalized to *Gapdh* expression. p value is shown as a result of a t-test. Related to Figure 5, Table S13.

Figure S12

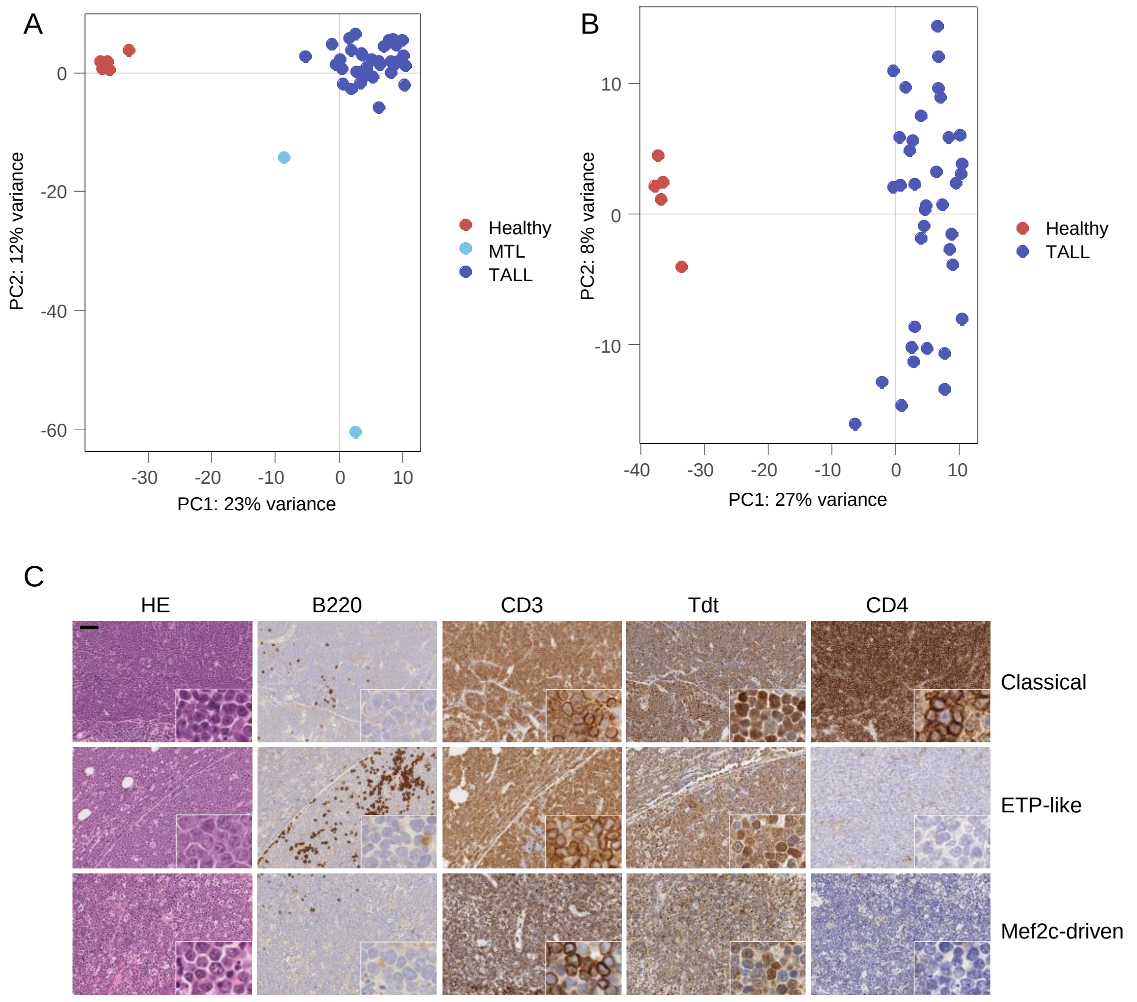

Supplementary Figure 12. Transcriptome- and IHC-based subtyping of T cell leukemias/lymphomas developed in Rosa26$^{PB/+}$;ATP2 mice and healthy controls. A, Mature T cell lymphomas (MTLs, light blue) cluster apart from T-ALL samples (dark blue) and healthy thymus (red). **B,** After removing MTL samples, T-ALL samples still cluster apart from healthy samples, but a variance in the PC2 can be detected. Only RNA isolated from thymus tissue was included in the analysis. Transcriptome-based subtyping confirms histological diagnoses, where MTLs did not show Tdt expression. **C,** Immunohistochemical characterization and sub-classification of T cell malignancies. CD4 positive T cell acute lymphoblastic leukemia/T cell lymphoblastic lymphoma (T-ALL/T-LBL) reflect the classical human T-ALL subtype. CD4 negative T-ALLs are immature pre- or pro-T ALLs. Scale bar, 50 µm. Related to Figure 6.

Figure S13

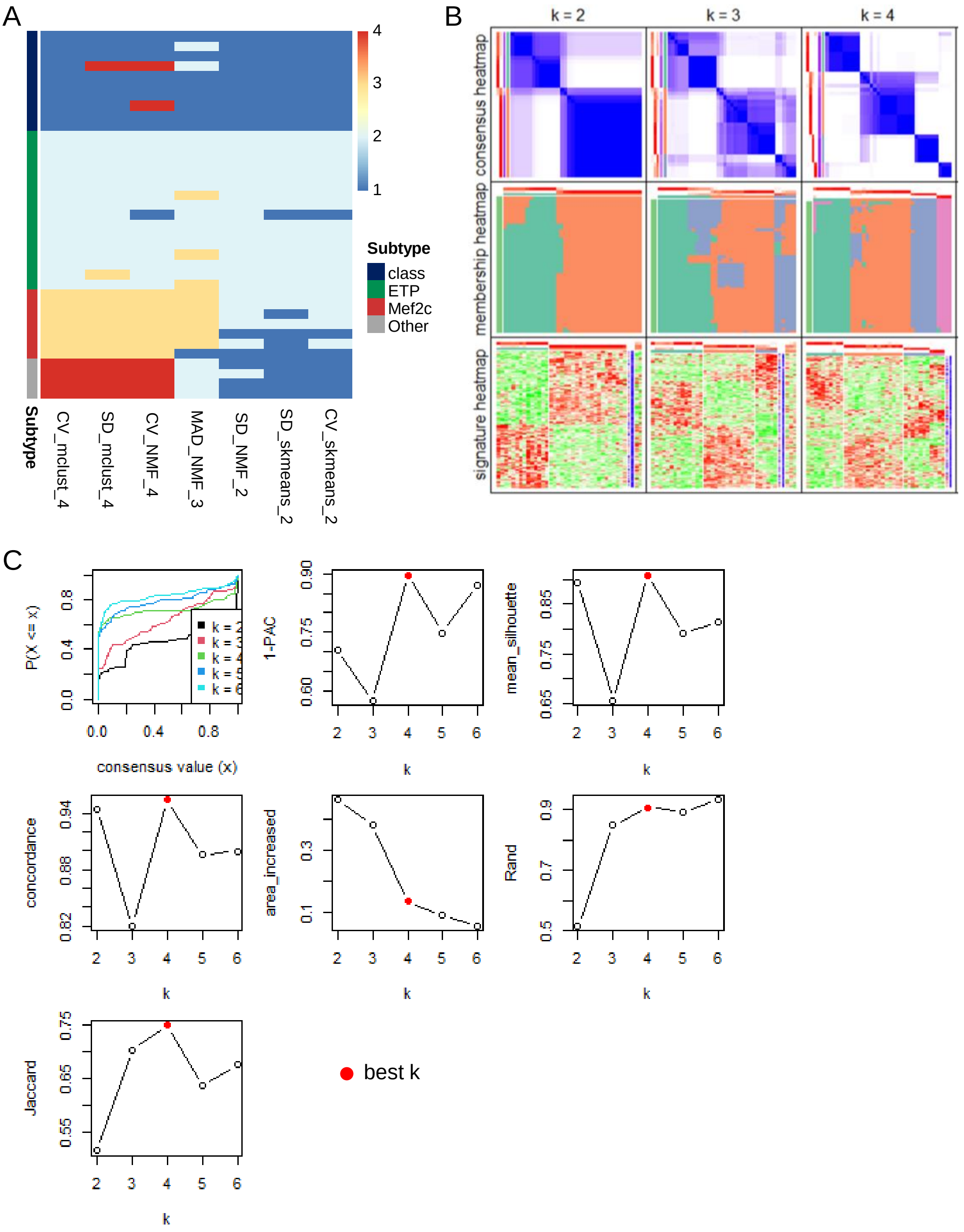

Supplementary Figure 13. Clustering of RNA-Seq data. The cola R package was used to compare multiple clustering approaches. **A,** Summary of subgroup assignments from highest ranked methods (full list available in Supplementary Table 14). The combination of top value method (SD, standard deviation; CV, coefficient of variance; MAD, median absolute deviation), clustering method (NMF, skmeans, mclust) and best k (2, 3, 4) is shown in the column names. Final subtype assignment is shown as column annotation. **B,** Consensus heatmap, membership heatmap and signature heatmap for the CV_mclust method for k=2, k=3 and k=4. **C,** Summary of statistical values for the CV_mclust method showing that k=4 is the best k. Related to Figure 6, Table S14.

Figure S14

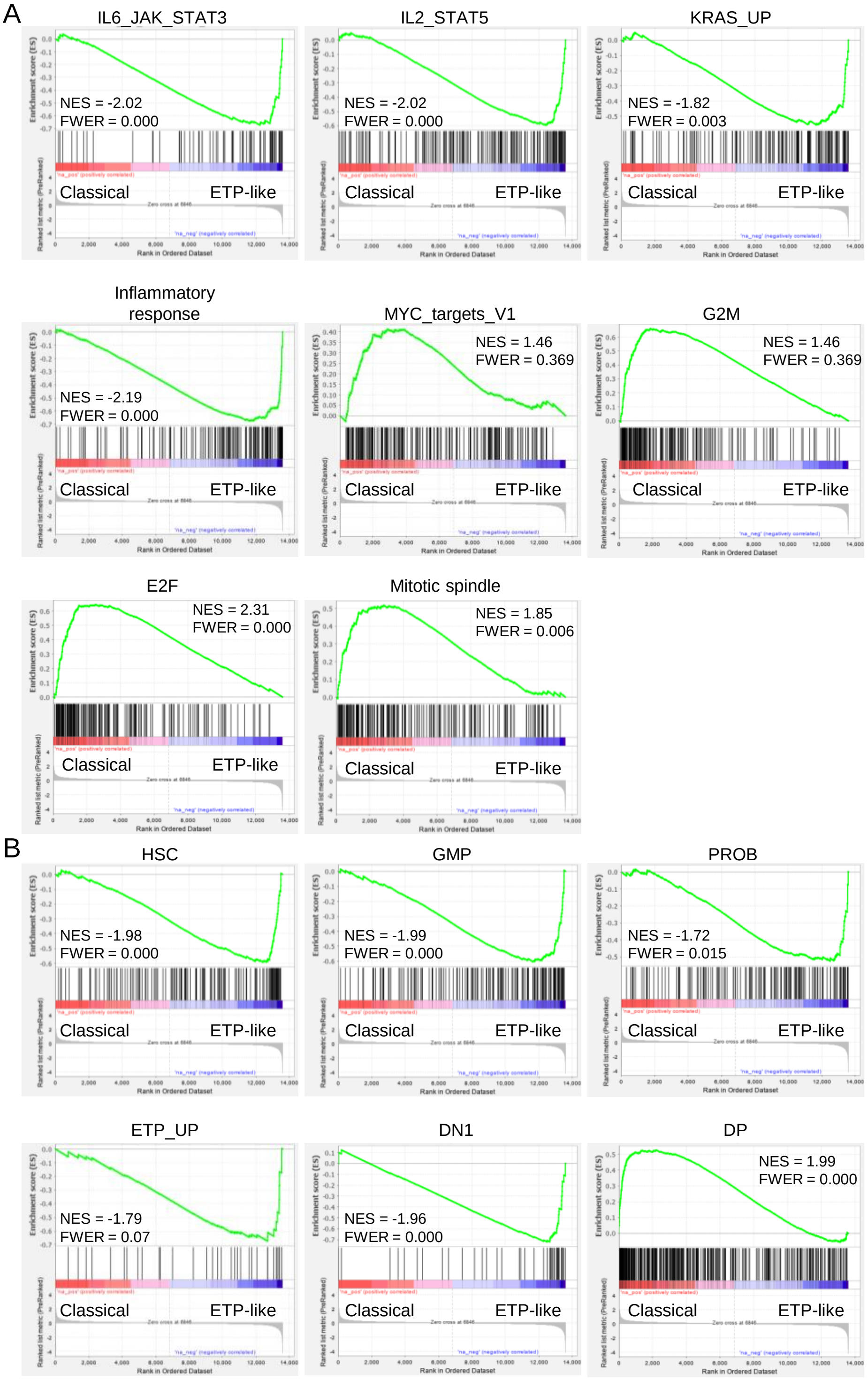

Supplementary Figure 14. Transcriptome-based differentiation of classical and ETP-like T-ALL subgroups. A, Gene set enrichment analysis (GSEA) using hallmark gene sets (h.all.v7.2.symbols.gmt) for pathway analysis between classical and ETP-like subgroups. **B,** GSEA using hematopoietic gene signatures (obtained from Laurenti et al. and Novershtern et al). Normalized enrichment score (NES) and FWER values are shown. Related to Figure 6, Table S15.

Figure S15

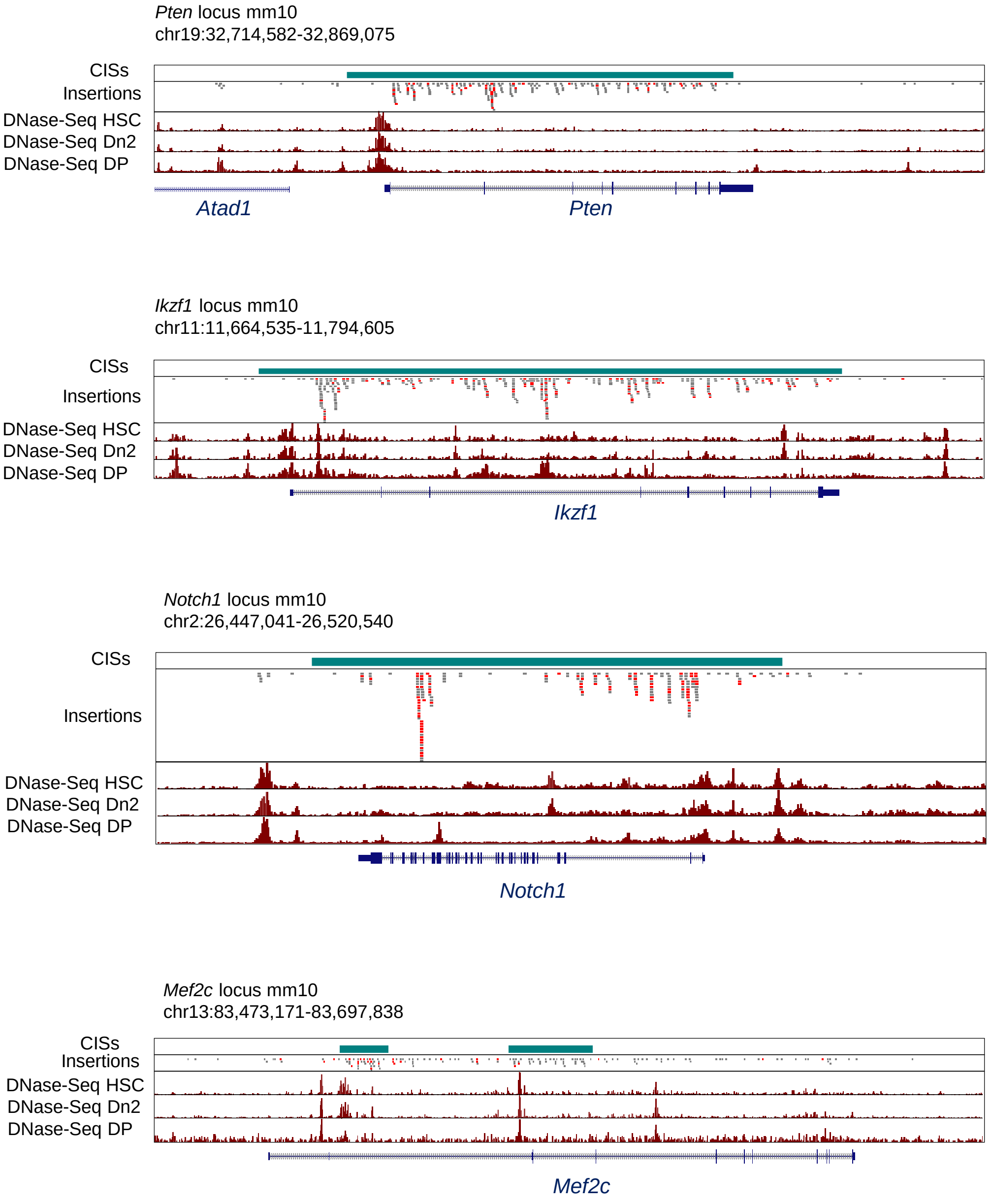

Supplementary Figure 15. Chromatin accessibility of major driver genes identified in the screen. UCSC Genome Browser image showing common insertion sites (CISs) and transposon insertions from *Rosa26PB/+;ATP2* mice. DNase-Seq data from different stages of T cell evolution are shown (publicly available data as listed in Supplementary Table 5). Protein-coding transcripts from the GENCODE gene annotation track are shown. High coverage insertions are shown in red, other insertions in grey. All T-ALL driver genes identified in the screen show a similar accessibility at their promoter in the course of T cell development. Insertion peaks are not primarily overlapping with peaks of open chromatin but show a broad distribution also in regions with less DNAse-Seq signal. Related to Figure 6.

Figure S16

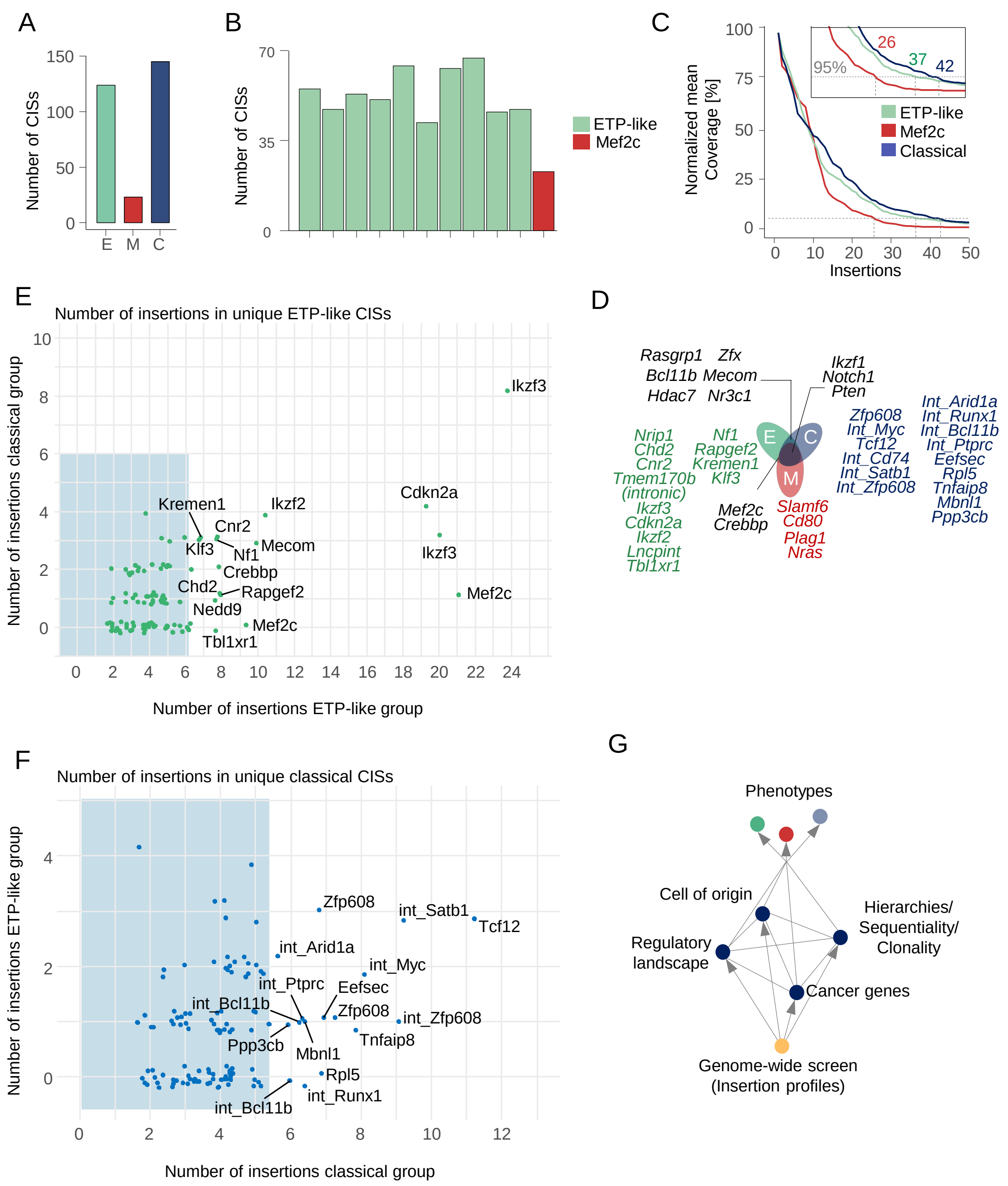

Supplementary Figure 16. Differences in common insertions sites between subgroups. A, Number of CISs identified in each T-ALL subgroup. Only CISs with at least two contributing insertions are considered. Number of unique insertions for each sample from ETP-like (n = 14), Mef2c-driven (n = 7) and classical (n = 8) T-ALL (*P < 0.05, Student's t test). **B,** Permutation test to identify number of CISs in ETP-like groups with same sample size as Mef2c-driven group (n = 7). Ten random selections of 7 samples of the ETP-like group (n = 14) were submitted to CIS analysis. CIS numbers are shown for the 10 permutation ETP samples and the Mef2c group. **C,** Normalized mean coverage of all samples in one subgroup is shown. Inlet depicts number of insertions contributing to 95% of the tumor. The Mef2c-driven subgroup is characterized by a lower number of insertions compared to the other groups. **D,** Shared and unique "top" CISs identified in the analyzed subgroups. Regulatory CISs are labelled with 'int' and 'intronic' for intergenic and intronic regulatory elements, respectively, and the putative target gene is reported. **E-F,** Comparison of CISs in ETP-like and classical subtype. Unique common insertion sites were selected by removing all CIS regions overlapping with a CIS region in the other group. Number of insertions in unique CIS regions were counted. Grey box marks region with CISs of low confidence. **E,** CIS regions unique to the ETP-like group are shown. CISs with more than 6 insertions are labelled with the respective gene overlapping the CIS region. **F,** CIS regions unique to the classical group are shown. CISs with more than 5 insertions are labelled with the respective gene overlapping the CIS region. Intergenic CISs are labelled with 'int' and the putative target gene. **G,** Integrative mapping of cellular and molecular determinants shaping tumor subtype evolution. Scheme showing the different layers of information that can be extracted from transposon insertion profiles to characterize the determinants of oncogenesis. The possibility to capture these determinants and their interplay as well as the related phenotypic outcomes in our model enables construction of multidimensional evolutionary maps. Related to Figure 6, Table S16-18.
