# Supplementary material for: In vivo interrogation of regulatory genomes reveals extensive quasi-insufficiency in cancer evolution
Source: Cell Genom. 2023 Mar 8;3(3):100276. doi: 10.1016/j.xgen.2023.100276 (PMC10025556; doi:10.1016/j.xgen.2023.100276)
Supplement: Document S2. Data S1–S5 [file mmc2.pdf]

Supplemental Data 1: Visualization of CISs from 'Intergenic Enhancer' group (related to list Figure 2E)

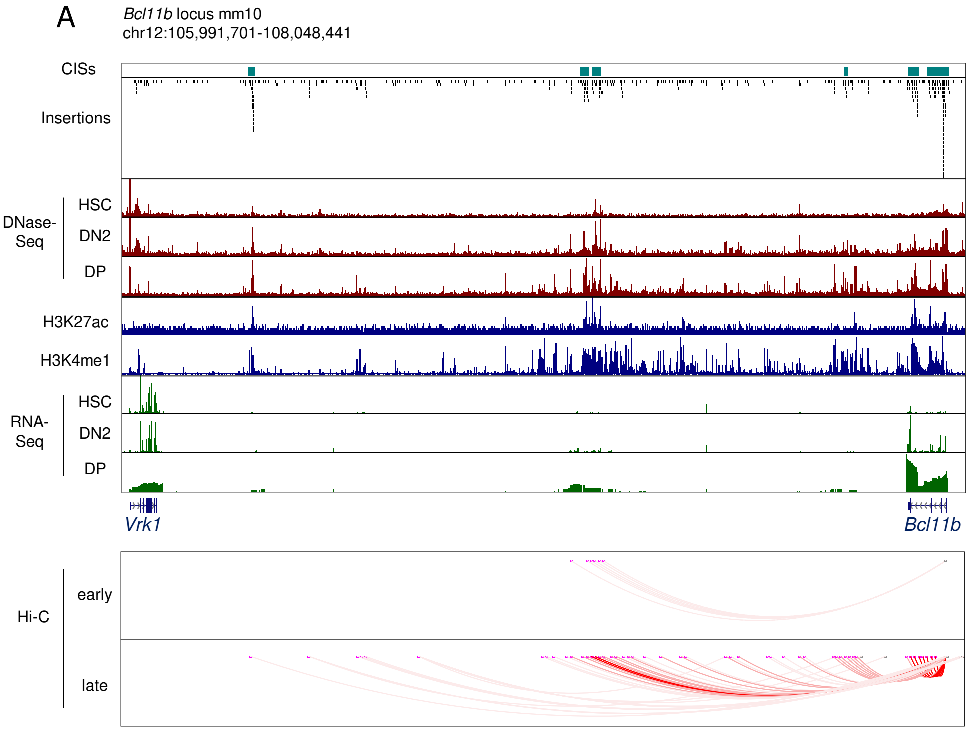

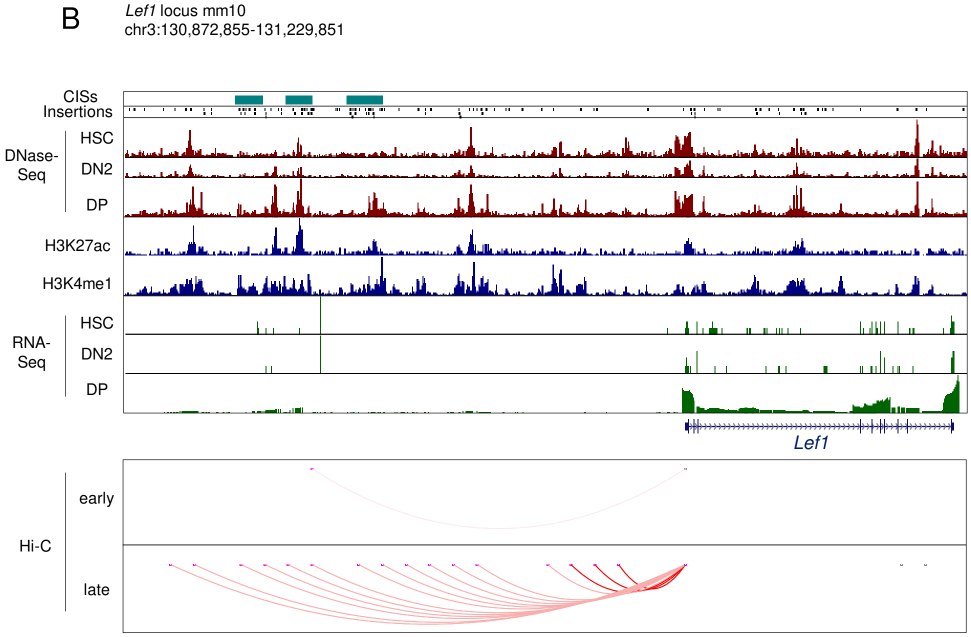

C

Hoxd cluster locus mm10
chr2:74,605,674-75,619,278

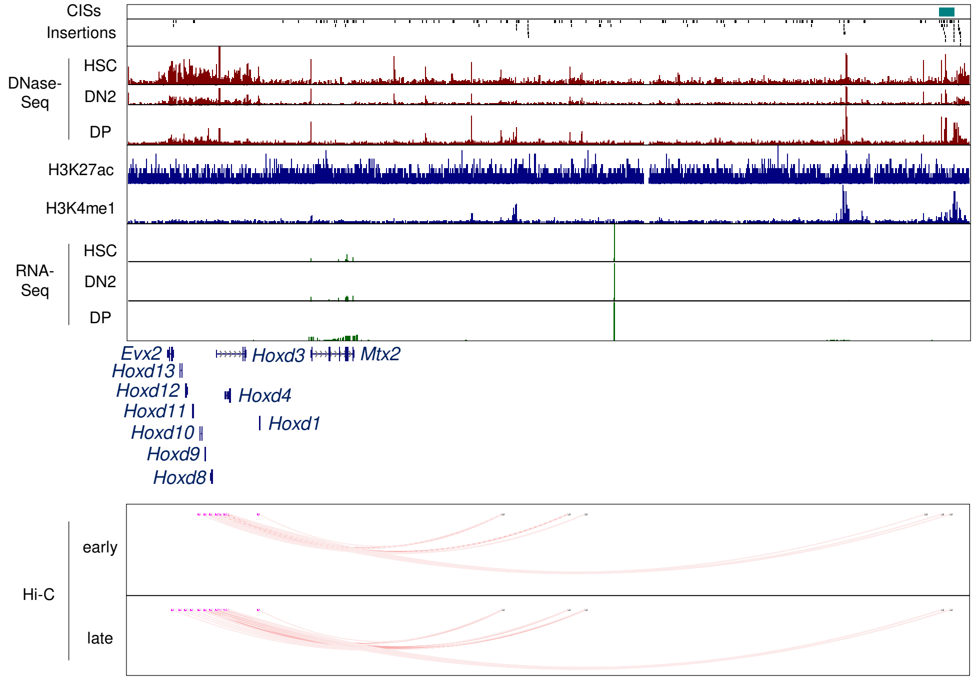

D

Cux1 locus mm10
chr5:136,271,704-136,635,922

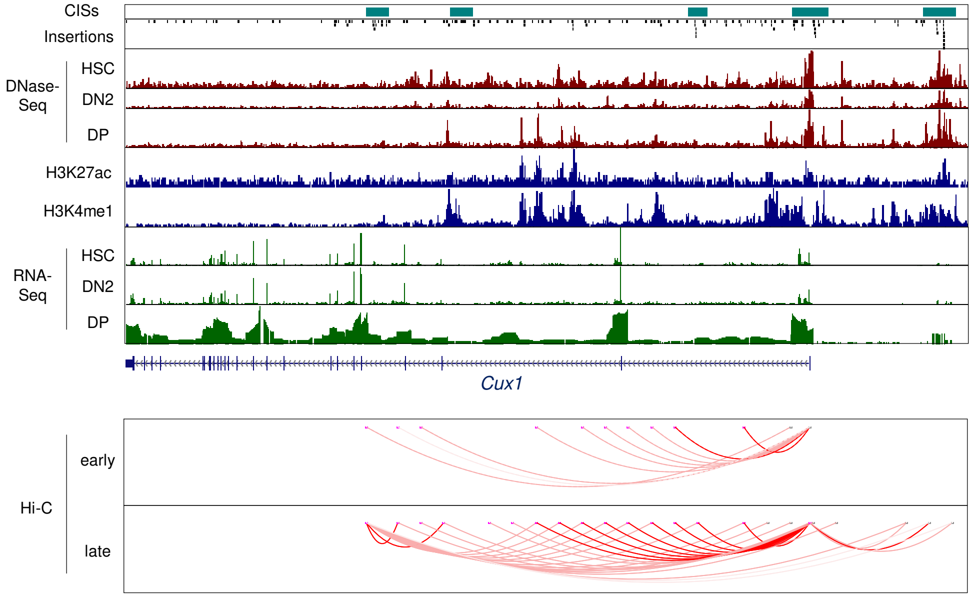

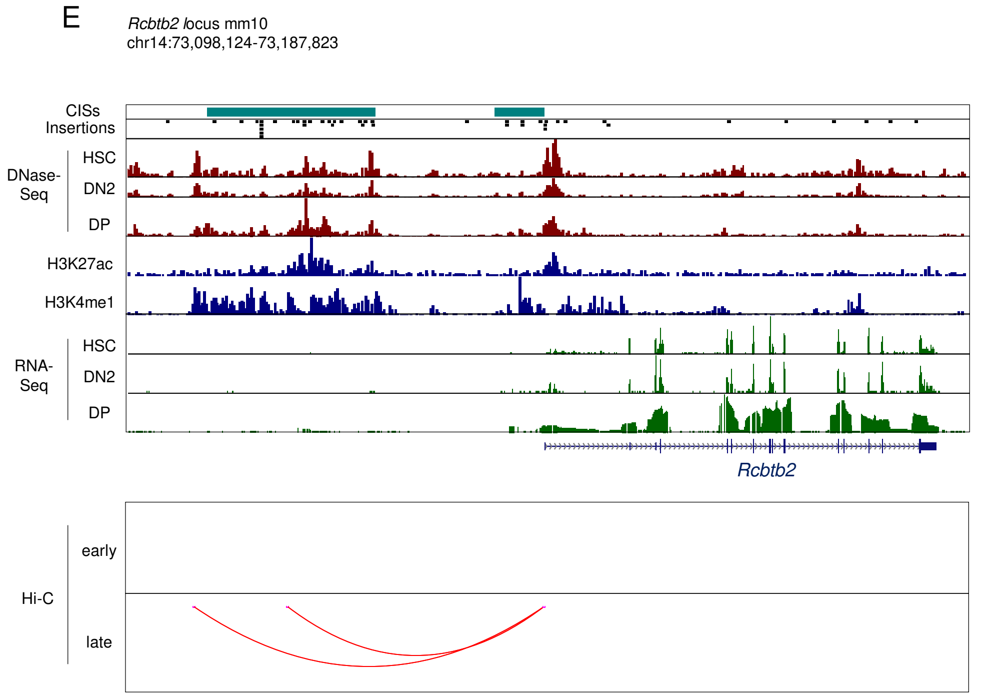
E
Rcbtb2 locus mm10
chr14:73,098,124-73,187,823
CISs
Insertions
DNase-Seq
HSC
DN2
DP
H3K27ac
H3K4me1
RNA-Seq
HSC
DN2
DP
Rcbtb2
Hi-C
early
late

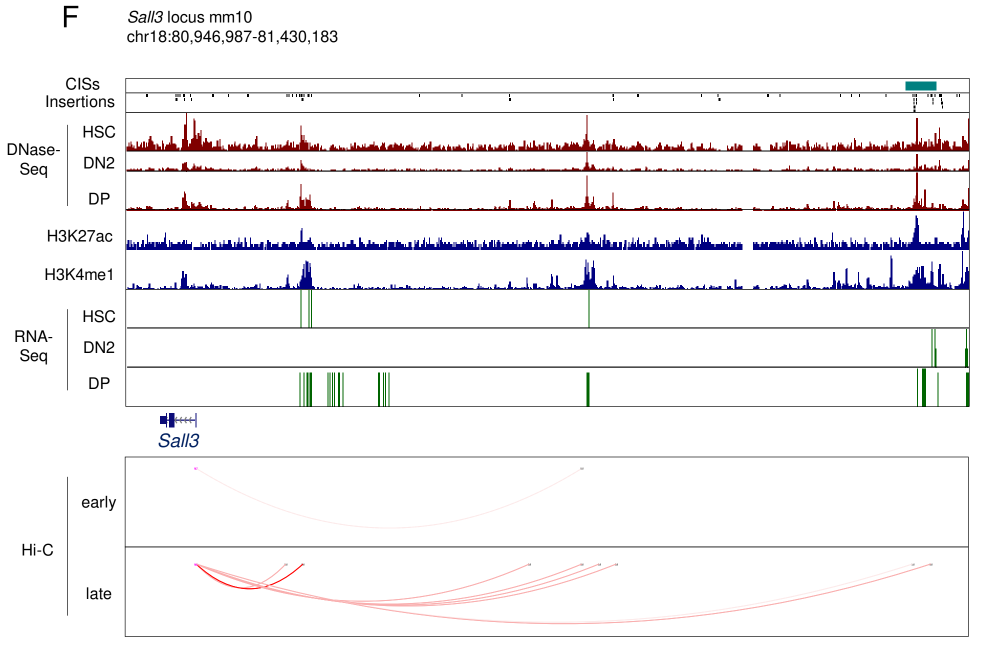
F
Sall3 locus mm10
chr18:80,946,987-81,430,183
CISs
Insertions
DNase-Seq
HSC
DN2
DP
H3K27ac
H3K4me1
RNA-Seq
HSC
DN2
DP
Sall3
Hi-C
early
late

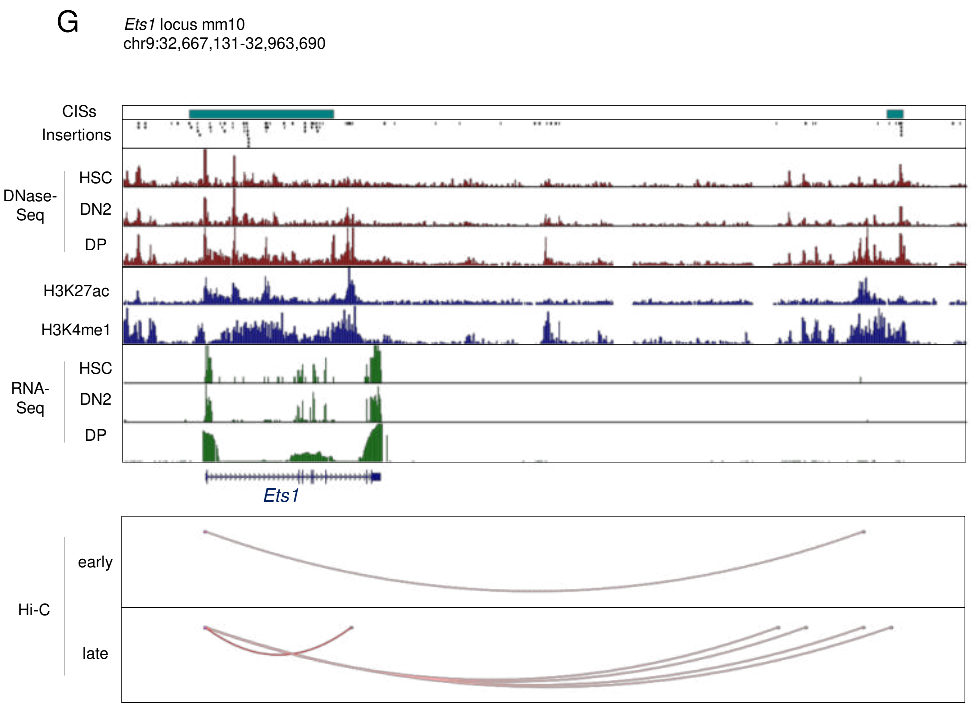
G
Ets1 locus mm10
chr9:32,667,131-32,963,690
CISs
Insertions
DNase-Seq
HSC
DN2
DP
H3K27ac
H3K4me1
RNA-Seq
HSC
DN2
DP
Ets1
Hi-C
early
late

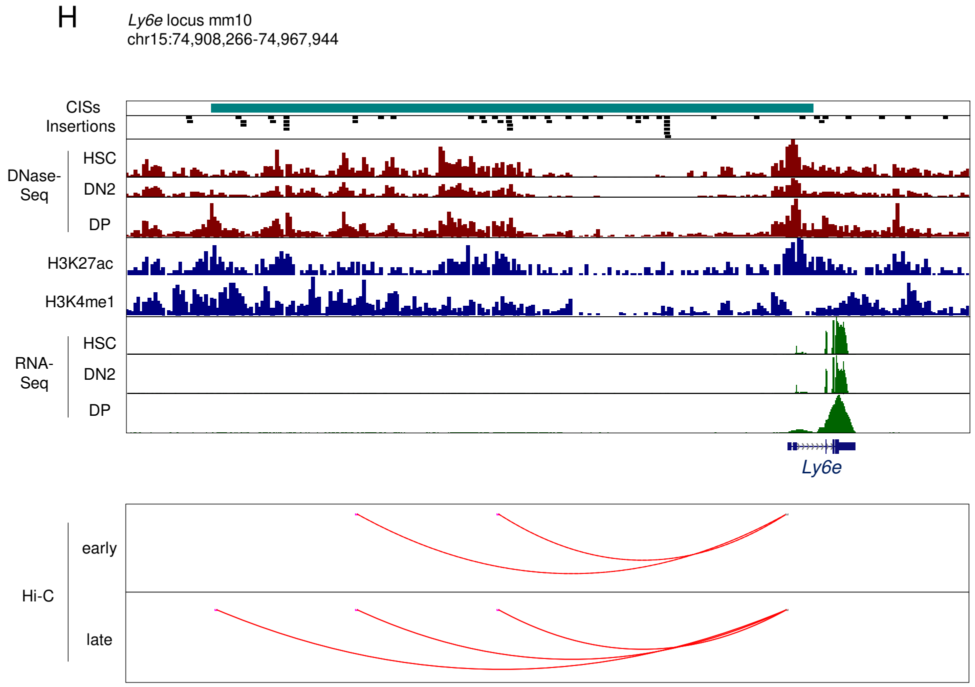
H
Ly6e locus mm10
chr15:74,908,266-74,967,944
CISs
Insertions
DNase-Seq
HSC
DN2
DP
H3K27ac
H3K4me1
RNA-Seq
HSC
DN2
DP
Ly6e
Hi-C
early
late

I

Rag2 locus mm10
chr2:101,530,890-101,669,435

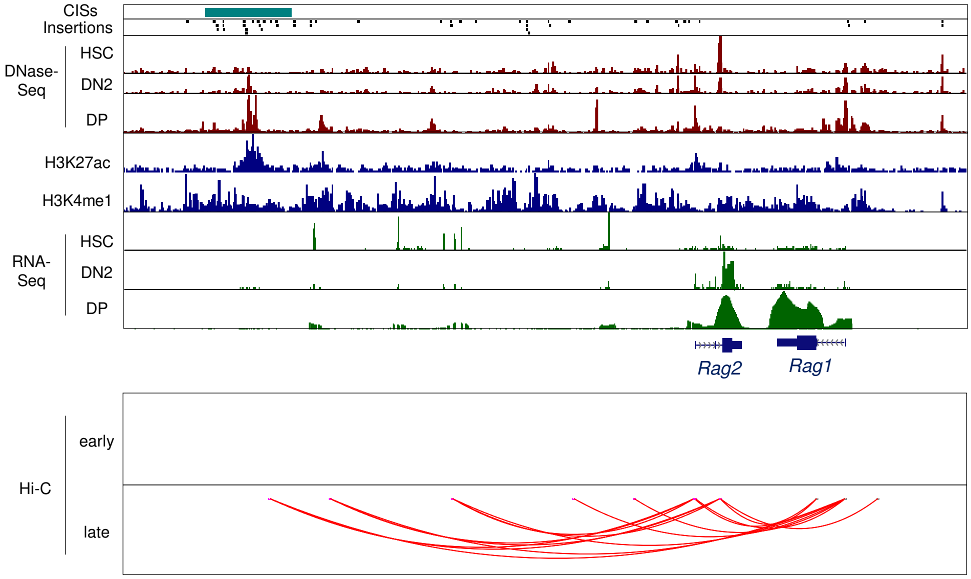

J

Satb1 locus mm10
chr17:51,707,947-52,487,539

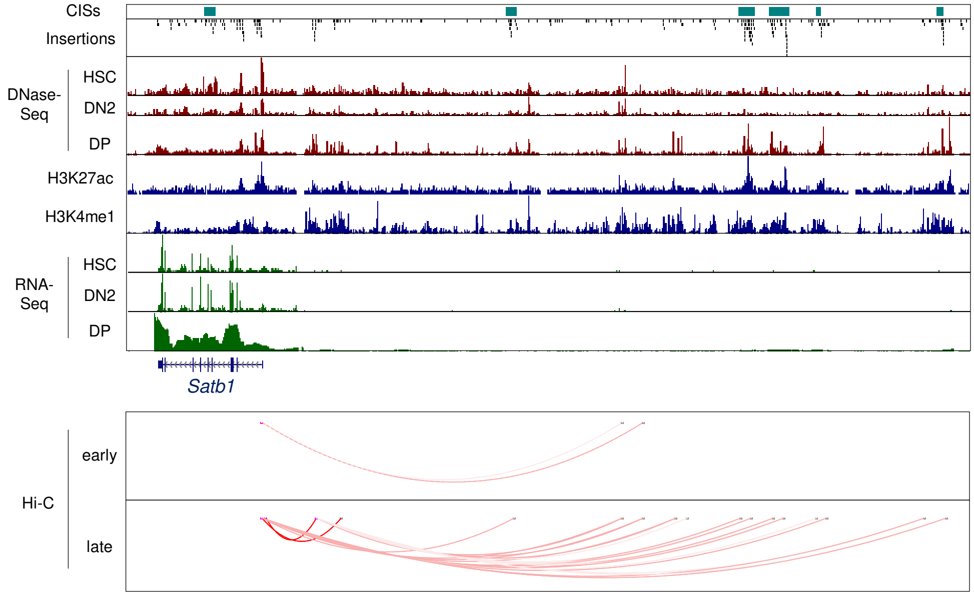

K

Tnfsf11 locus mm10
chr14:78,267,745-78,452,717

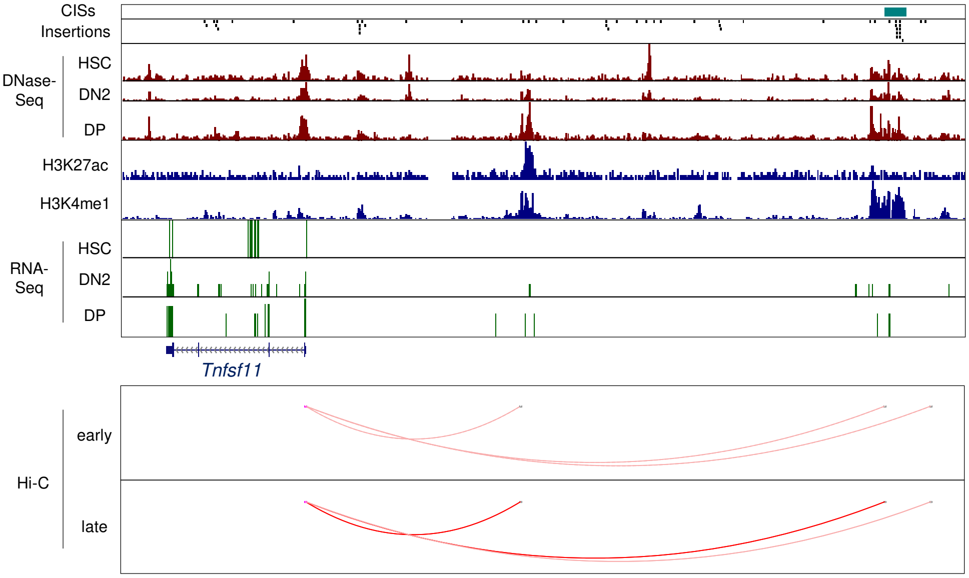

L

Runx1 locus mm10
chr16:92,564,243-93,330,778

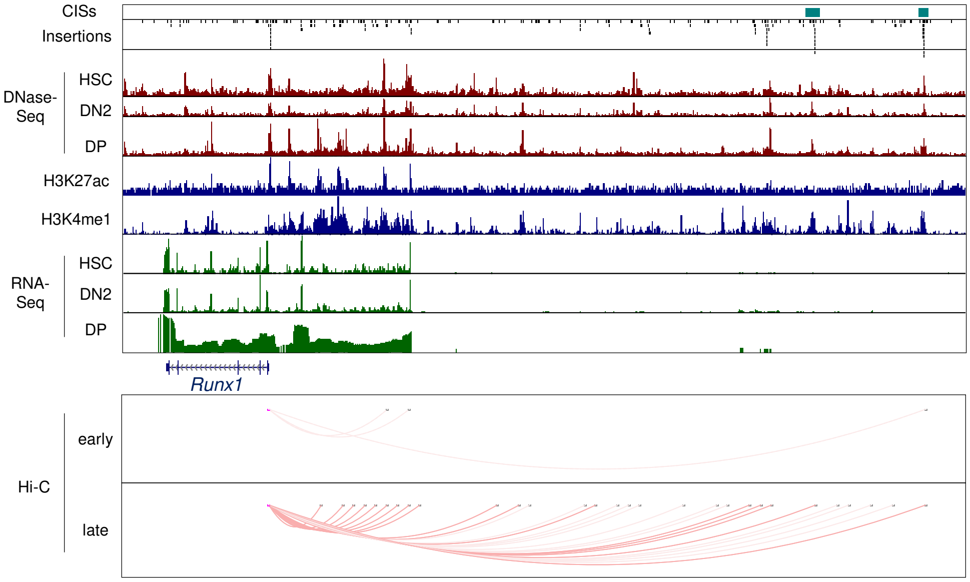

M

Tfrc locus mm10
chr16:32,510,134-32,637,214

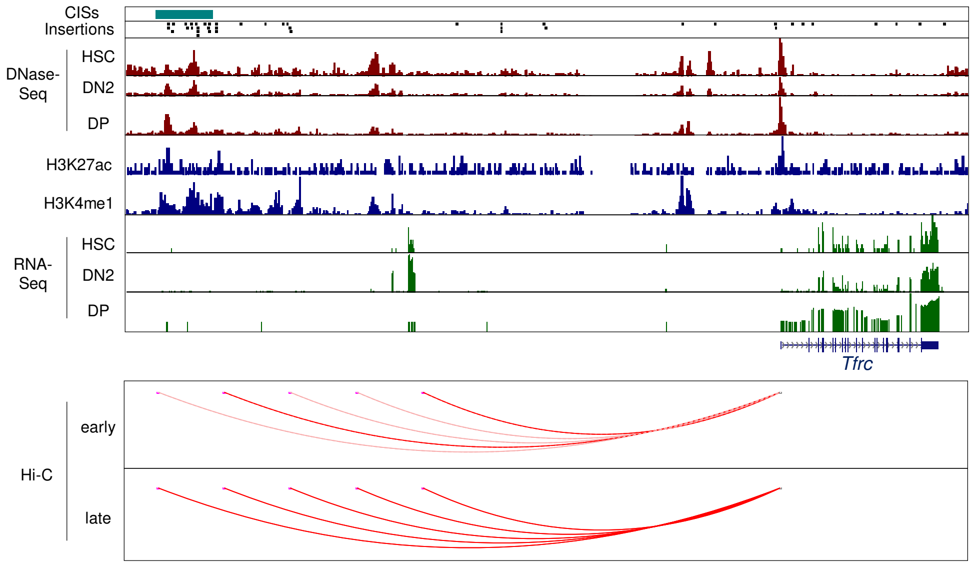

N

Ikzf2 locus mm10
chr1:69,522,878-69,796,999

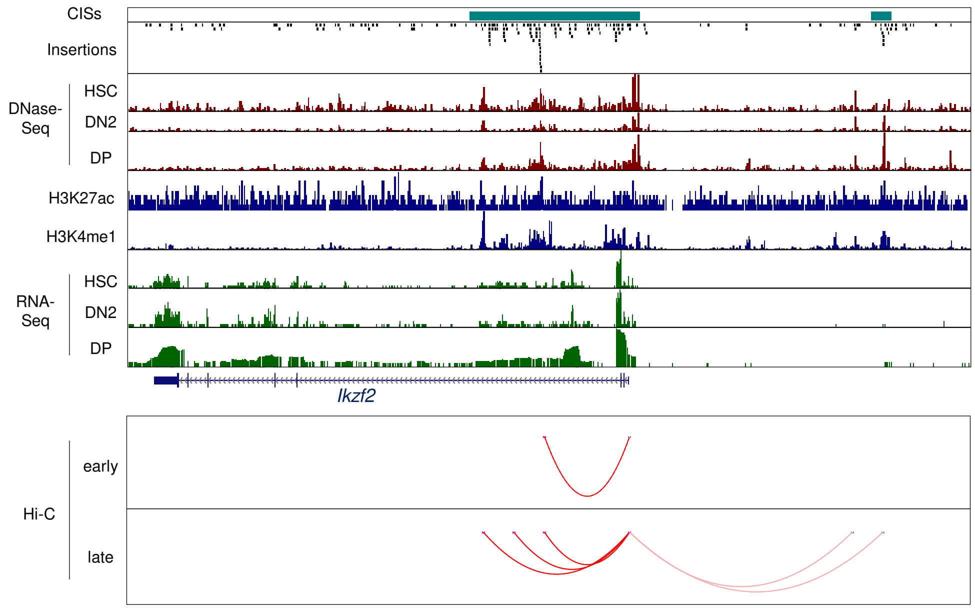

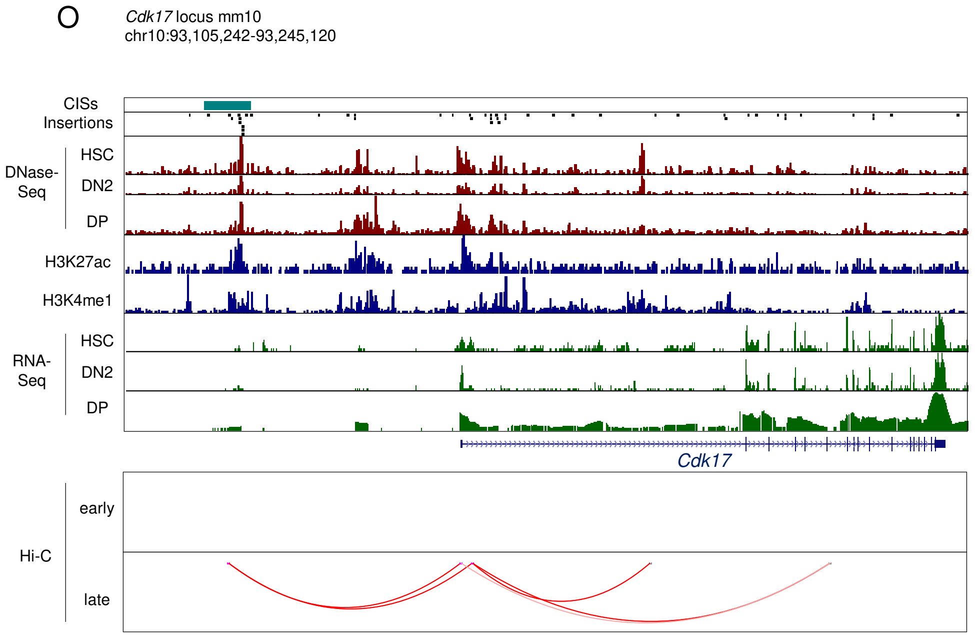
O
Cdk17 locus mm10
chr10:93,105,242-93,245,120
CISs
Insertions
DNase-Seq
HSC
DN2
DP
H3K27ac
H3K4me1
RNA-Seq
HSC
DN2
DP
Cdk17
Hi-C
early
late

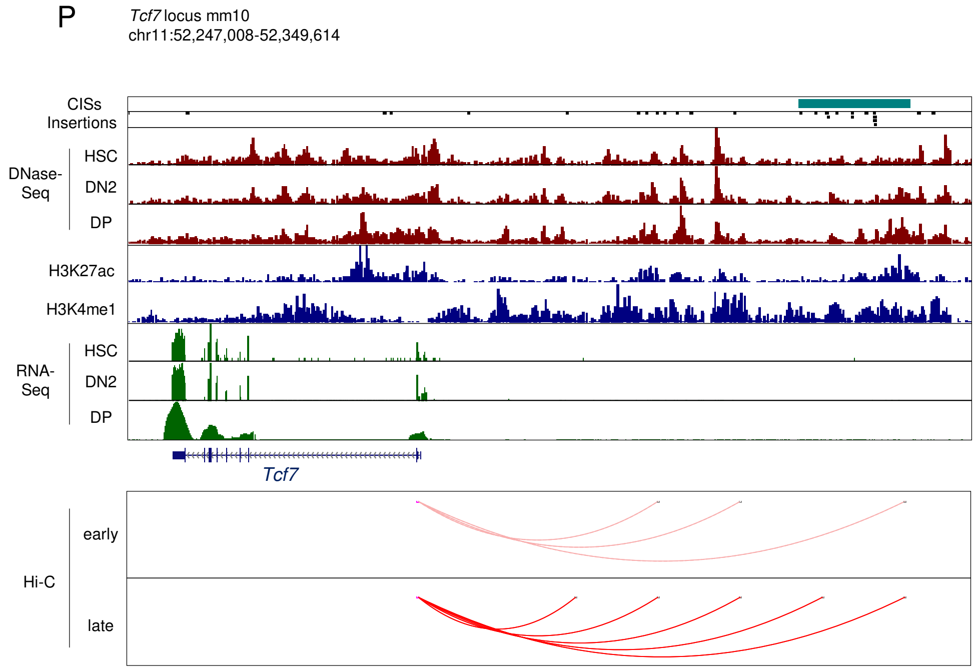
P
Tcf7 locus mm10
chr11:52,247,008-52,349,614
CISs
Insertions
DNase-Seq
HSC
DN2
DP
H3K27ac
H3K4me1
RNA-Seq
HSC
DN2
DP
Tcf7
Hi-C
early
late

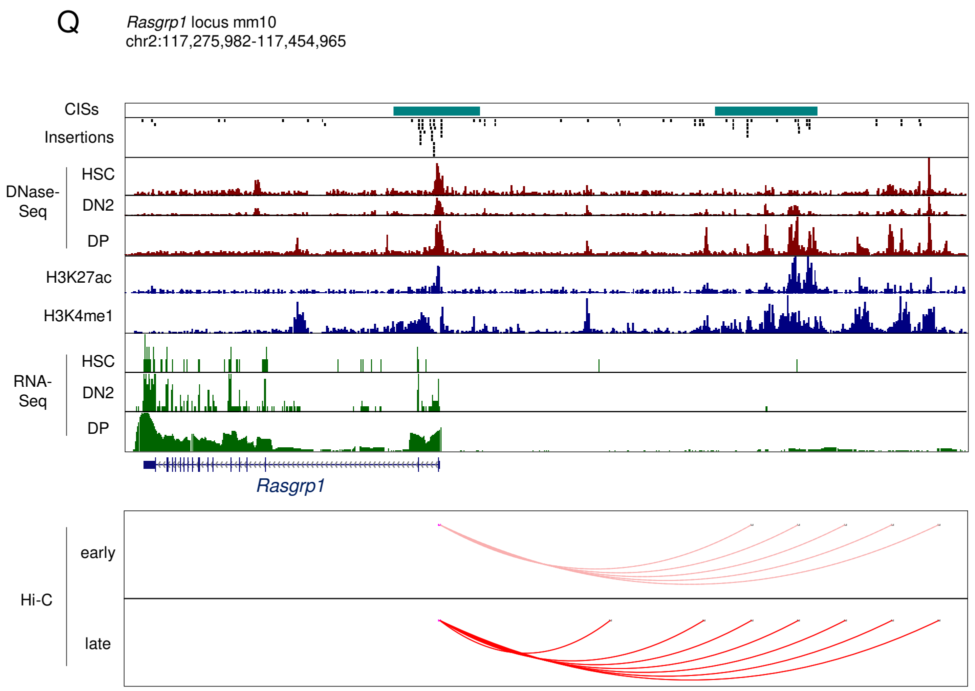

Supplementary Data 1. Visualization of identified regulatory CISs from the group 'Intergenic Enhancer'. A,-Q, UCSC Genome Browser image showing common insertion sites (CISs) and transposon insertions from Rosa26$^{PB/+}$;ATP2 mice. H3K27ac and H3K4me1 tracks from double-positive T cells, as well as DNase-Seq, RNA-Seq and Hi-C data from different stages of T cell evolution are shown (publicly available data as listed in Supplementary Table 5). Only protein-coding and CIS-overlapping transcripts from the GENCODE gene annotation track are shown. CISs identified in this group most likely contain an intergenic enhancer element. Related to Figure 2E, Supplementary Table 5.

Supplementary Data 2: Visualization of CISs from intergenic 'ncRNA plus Enhancer' group (related to list Figure 2E)

A

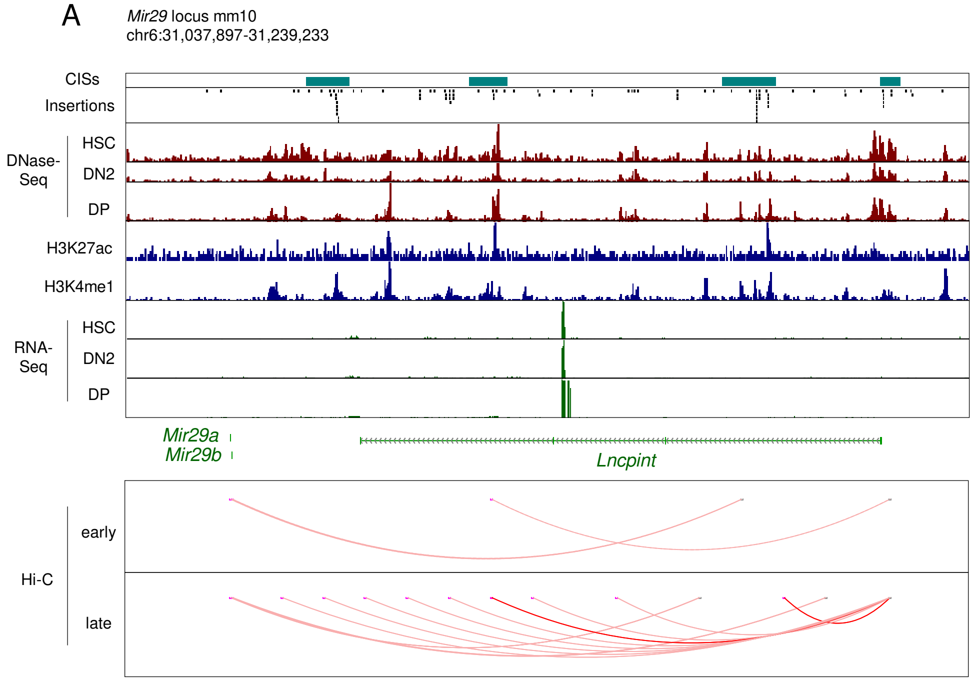

B

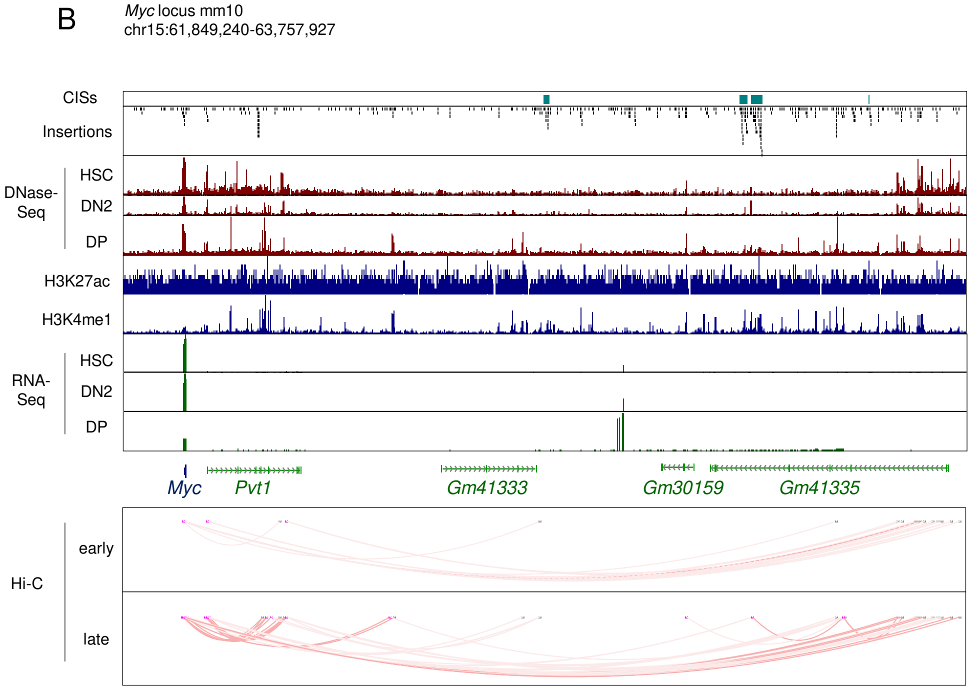

C

Myb locus mm10
chr10:21,081,054-21,260,609

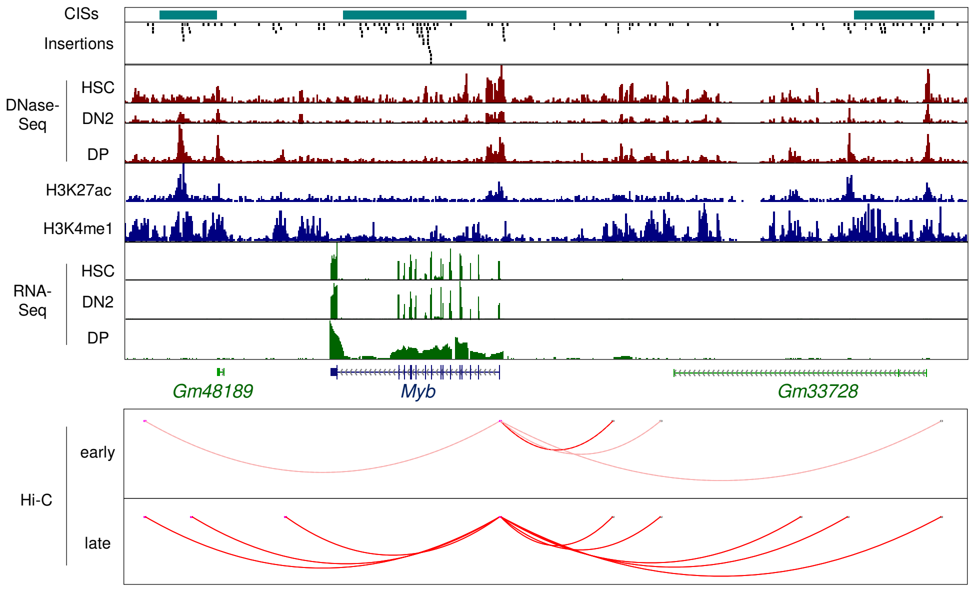

D

Ikzf1 locus mm10
chr11:11,541,510-11,788,982

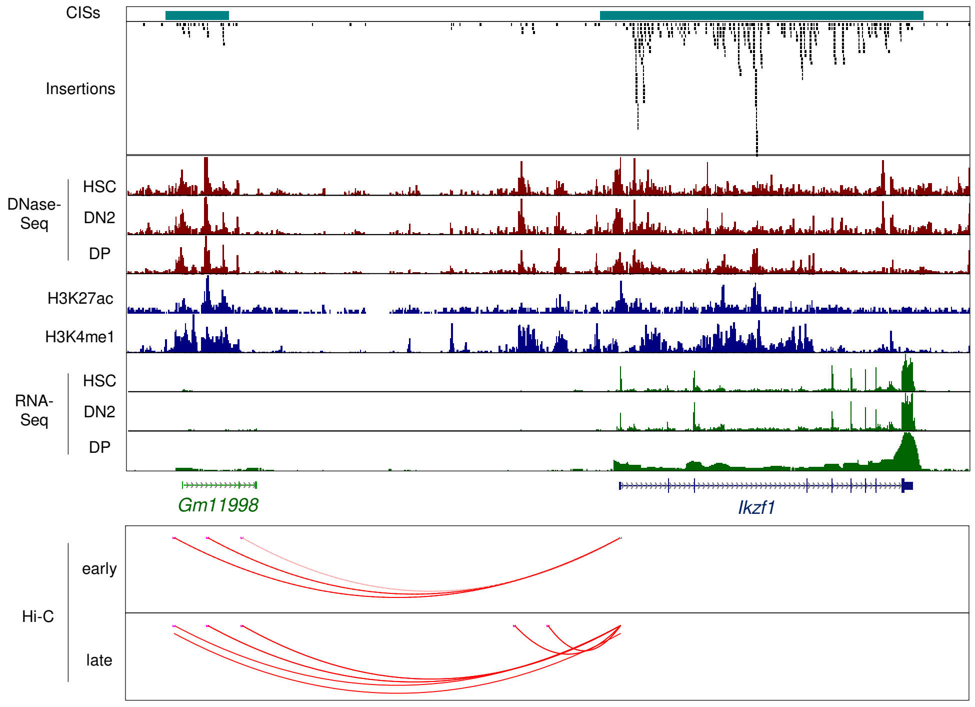

E

Ptprc locus mm10
chr1:137,640,507-138,183,374

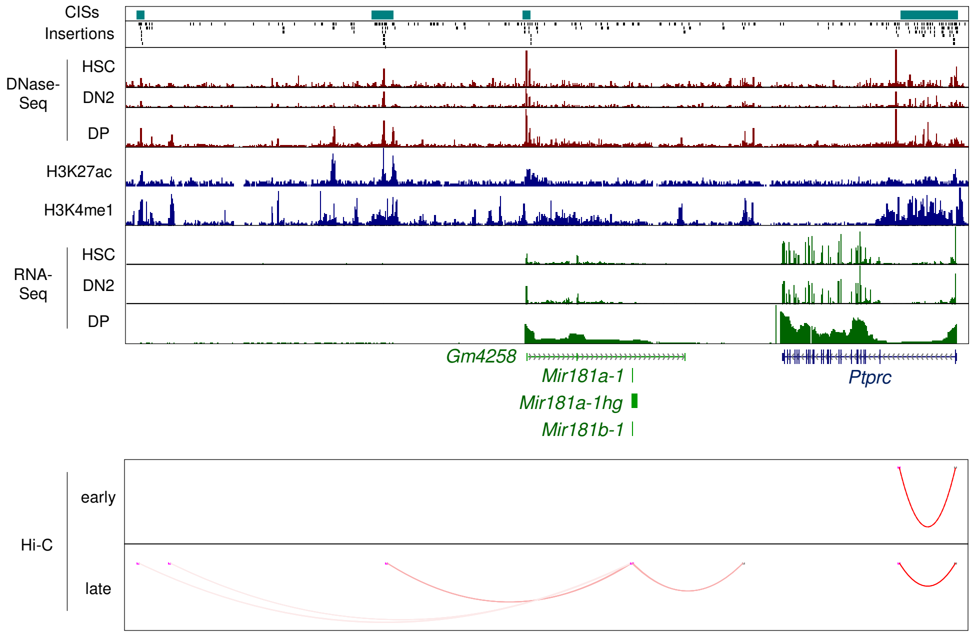

F

Mirlet7c-2 locus mm10
chr15:85,605,579-85,734,543

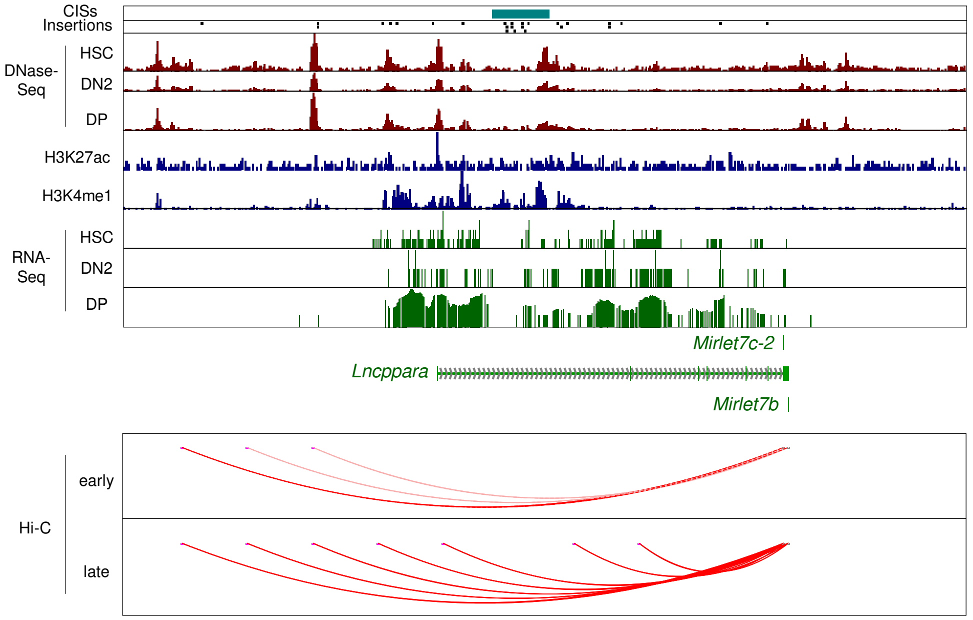

G

Il6st locus mm10
chr13:112,406,592-112,518,571

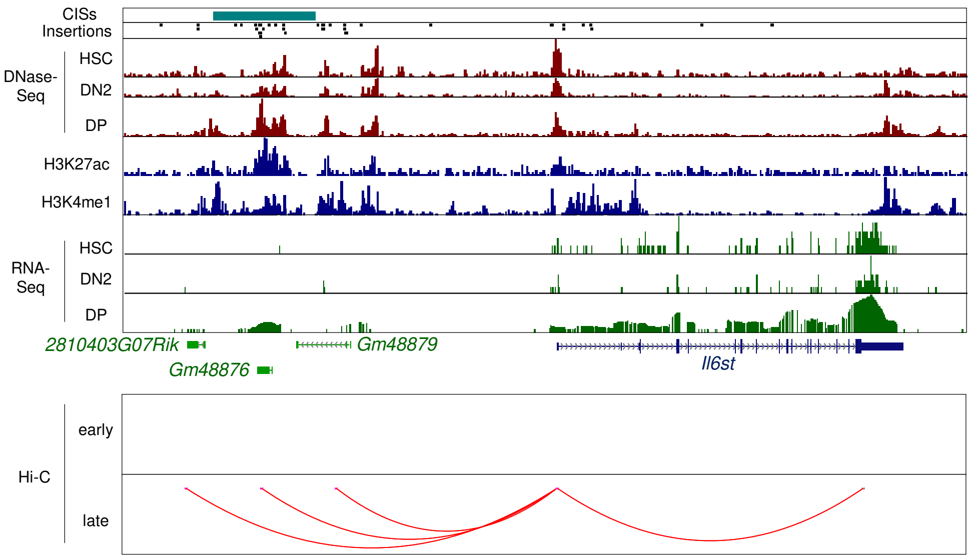

H

Kctd1 locus mm10
chr18:14,949,133-15,243,953

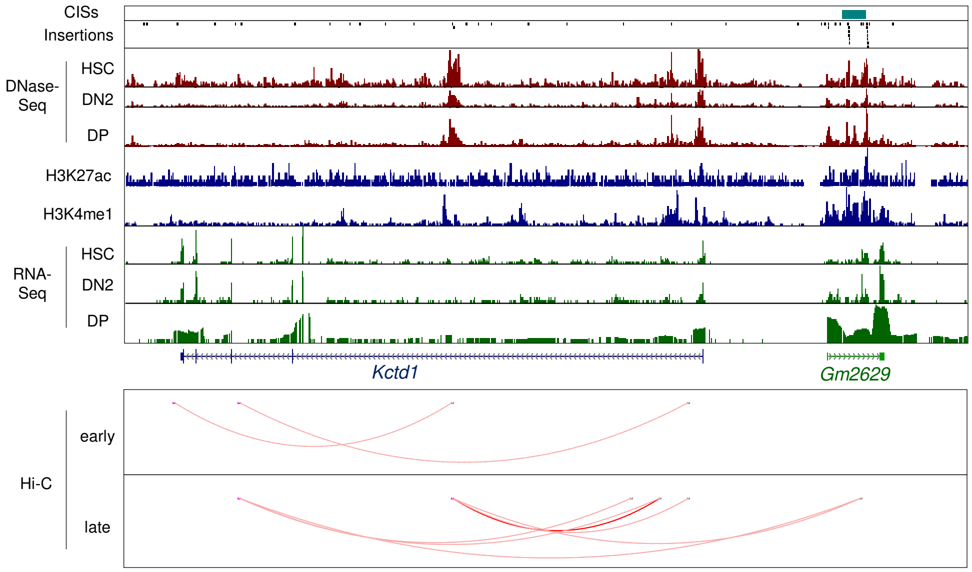

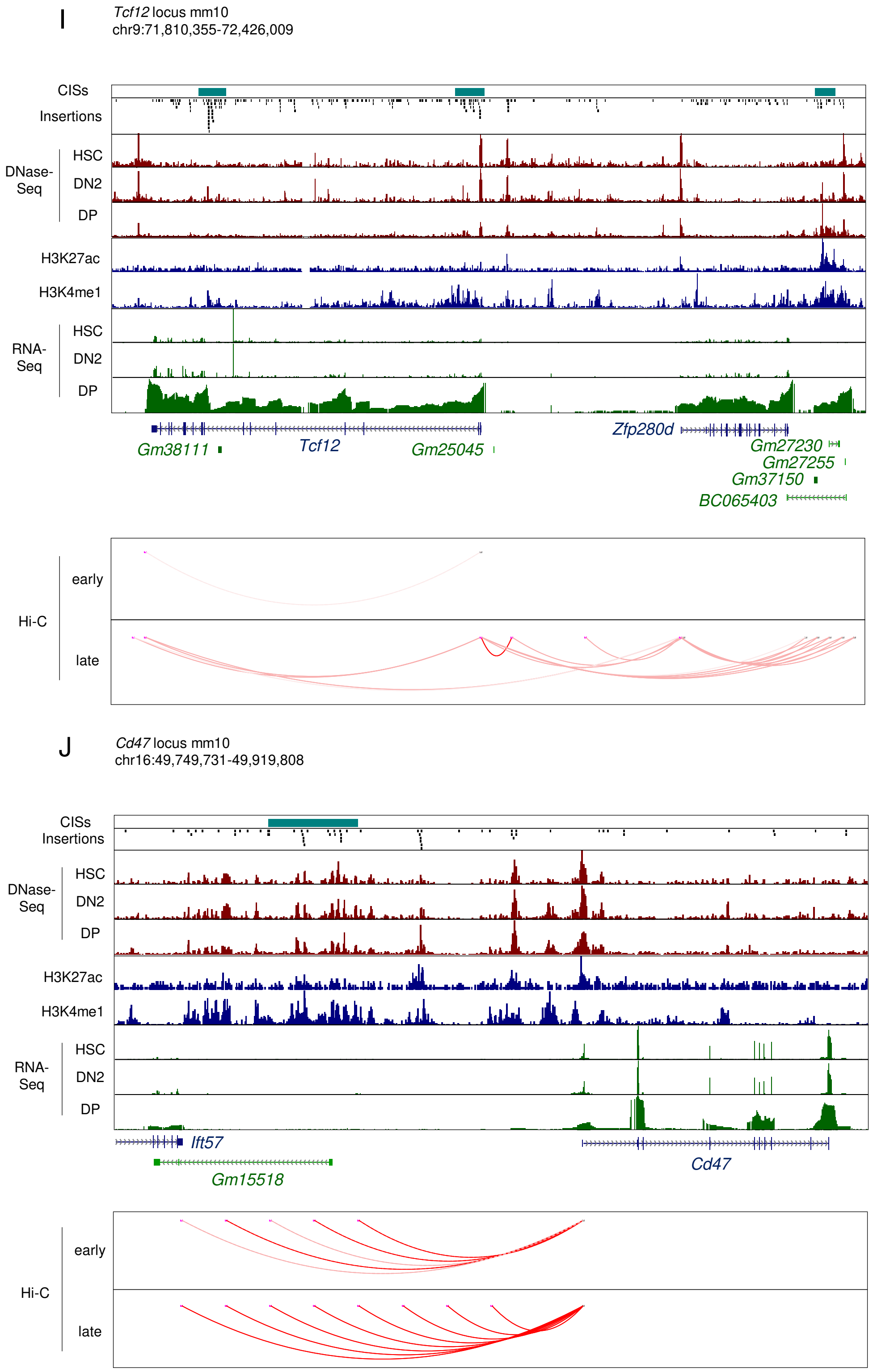
I
Tcf12 locus mm10
chr9:71,810,355-72,426,009
CISs
Insertions
DNase-Seq
HSC
DN2
DP
H3K27ac
H3K4me1
RNA-Seq
HSC
DN2
DP
Gm38111
Tcf12
Gm25045
Zfp280d
Gm27230
Gm27255
Gm37150
BC065403
Hi-C
early
late
J
Cd47 locus mm10
chr16:49,749,731-49,919,808
CISs
Insertions
DNase-Seq
HSC
DN2
DP
H3K27ac
H3K4me1
RNA-Seq
HSC
DN2
DP
Ift57
Gm15518
Cd47
Hi-C
early
late

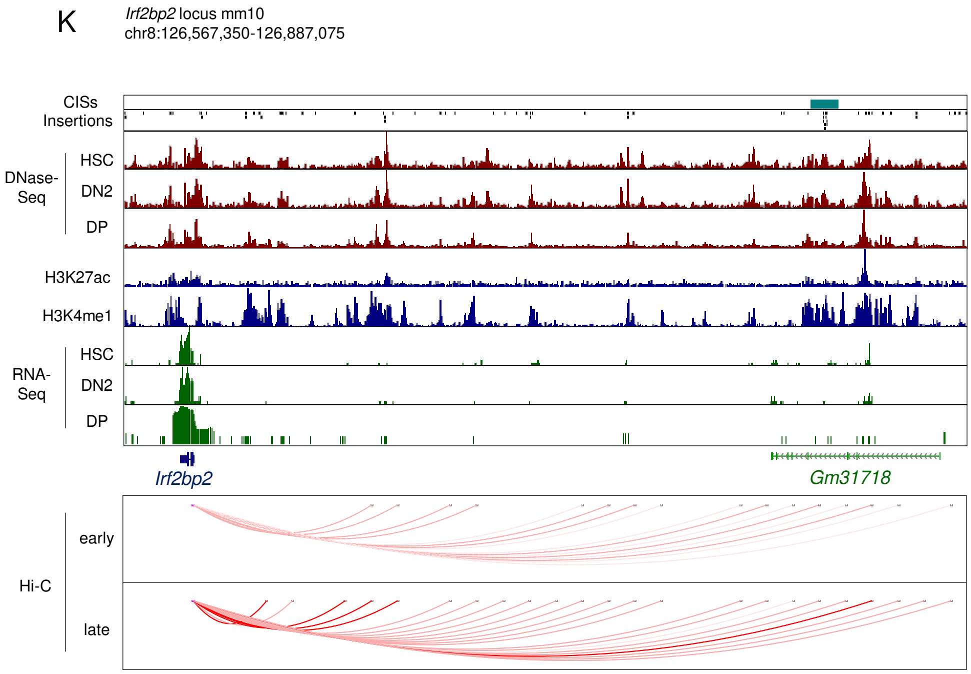

Supplementary Data 2. Visualization of identified regulatory CISs from the group 'intergenic ncRNA plus Enhancer'. A,-K, UCSC Genome Browser image showing common insertion sites (CISs) and transposon insertions from Rosa26$^{PB/+}$;ATP2 mice. H3K27ac and H3K4me1 tracks from double-positive T cells, as well as DNase-Seq, RNA-Seq and Hi-C data from different stages of T cell evolution are shown (publicly available data as listed in Supplementary Table 5). Protein-coding and non-protein-coding transcripts from the GENCODE gene annotation track are shown. CISs identified in this group overlap with a non-protein coding transcript and a putative regulatory element. Related to Figure 2E, Supplementary Table 5.

Supplementary Data 3: Visualization of CISs from ‘PC transcript plus ncRNA’ group (related to list Figure 2E)

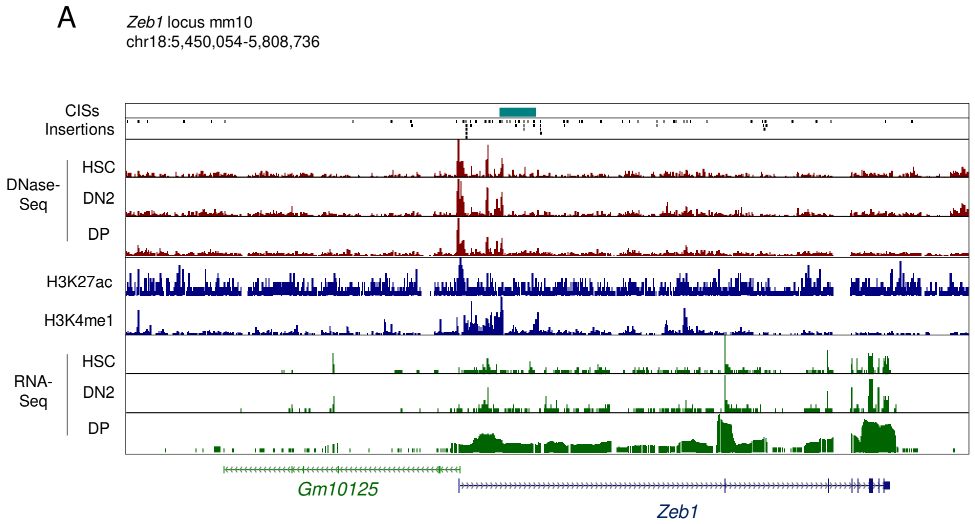

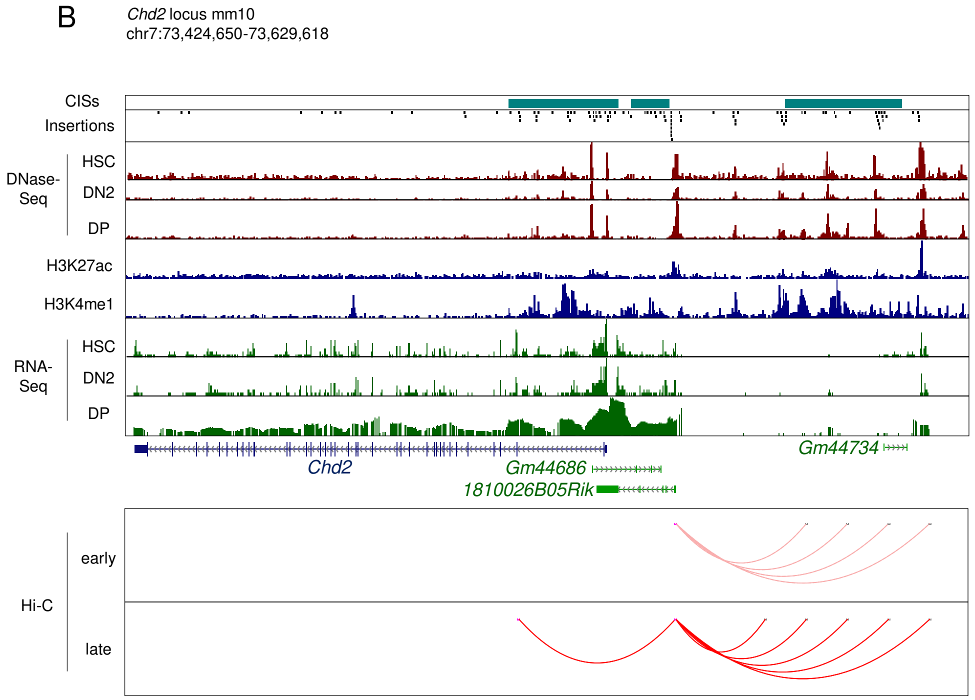

C

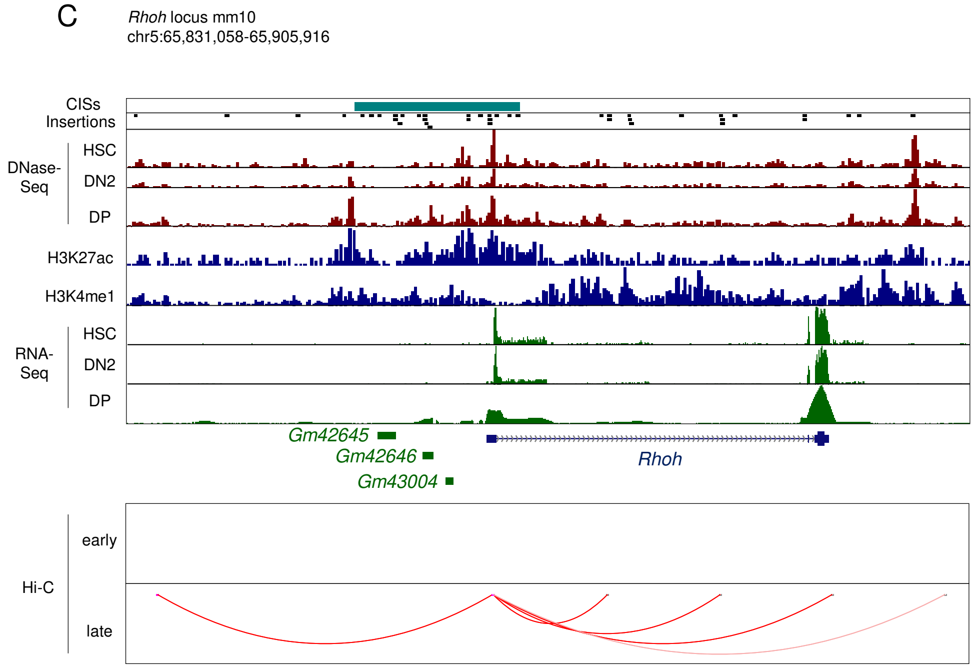

Rhoh locus mm10
chr5:65,831,058-65,905,916
CISs
Insertions
DNase-Seq
HSC
DN2
DP
H3K27ac
H3K4me1
RNA-Seq
HSC
DN2
DP
Gm42645
Gm42646
Gm43004
Rhoh
Hi-C
early
late

D

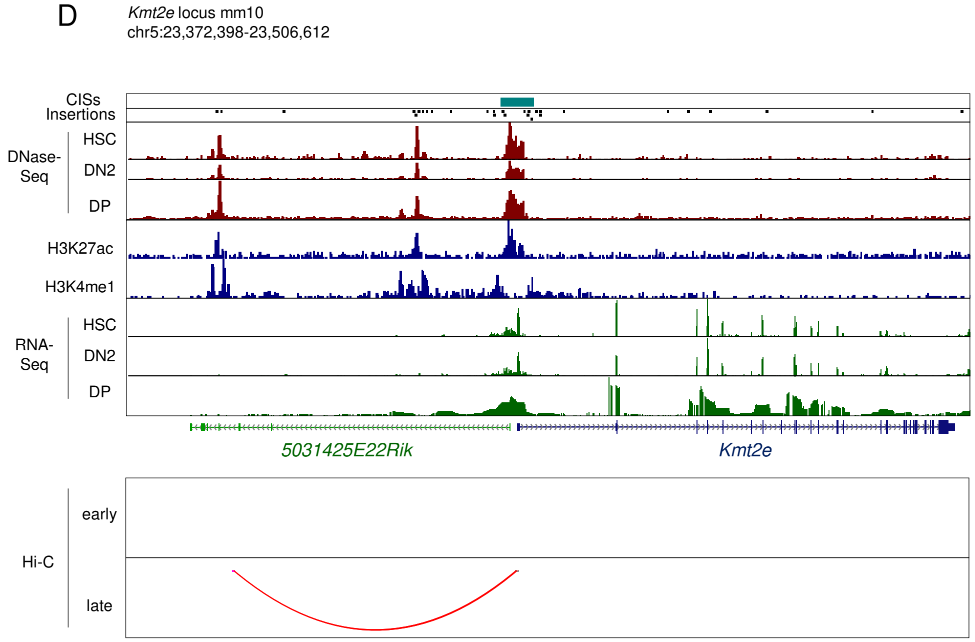

Kmt2e locus mm10
chr5:23,372,398-23,506,612
CISs
Insertions
DNase-Seq
HSC
DN2
DP
H3K27ac
H3K4me1
RNA-Seq
HSC
DN2
DP
5031425E22Rik
Kmt2e
Hi-C
early
late

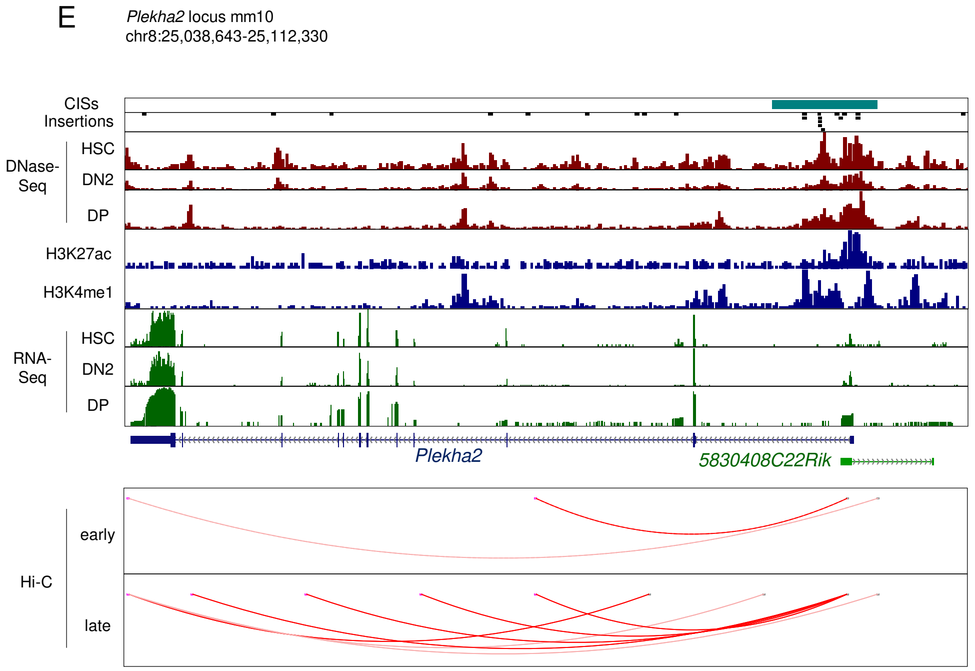

Supplementary Data 3. Visualization of identified regulatory CISs from the group 'PC transcript plus ncRNA'. A,-E, UCSC Genome Browser image showing common insertion sites (CISs) and transposon insertions from Rosa26$^{PB/+}$;ATP2 mice. H3K27ac and H3K4me1 tracks from double-positive T cells, as well as DNase-Seq, RNA-Seq and Hi-C data from different stages of T cell evolution are shown (publicly available data as listed in Supplementary Table 5). Protein-coding and non-protein-coding transcripts from the GENCODE gene annotation track are shown. CISs identified in this group overlap with a protein-coding and a non-protein coding transcript. Related to Figure 2E, Supplementary Table 5.

Supplementary Data 4: Visualization of CISs from ‘PC transcript plus Intergenic Enhancer’ group (related to list Figure 2E)

A

Sox4 locus mm10
chr13:28,933,942-28,969,570

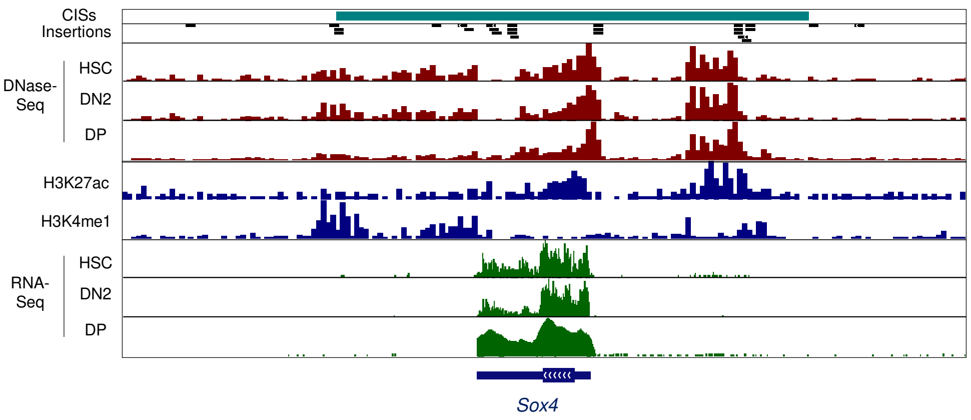

B

Gfi1 locus mm10
chr5:107,710,833-107,760,584

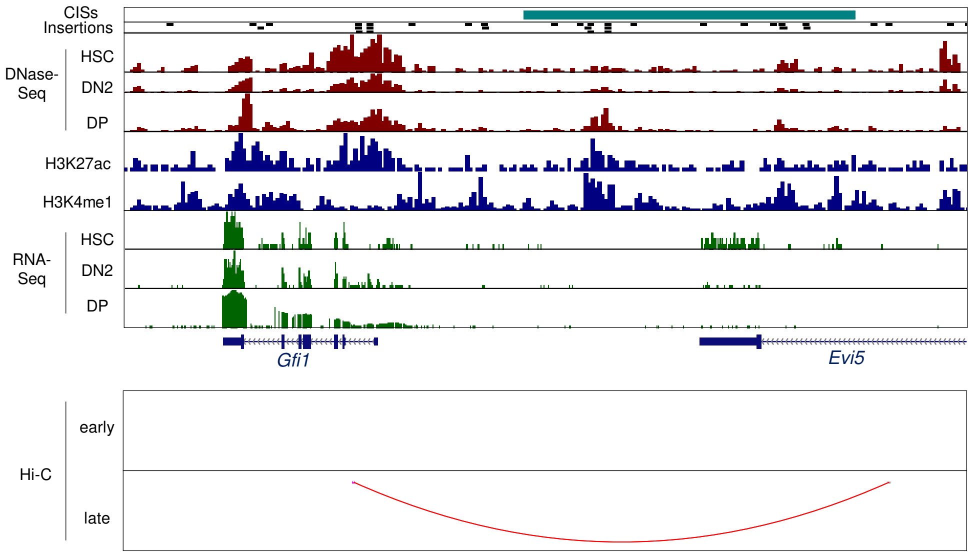

C

Dntt locus mm10
chr19:41,008,673-41,060,807

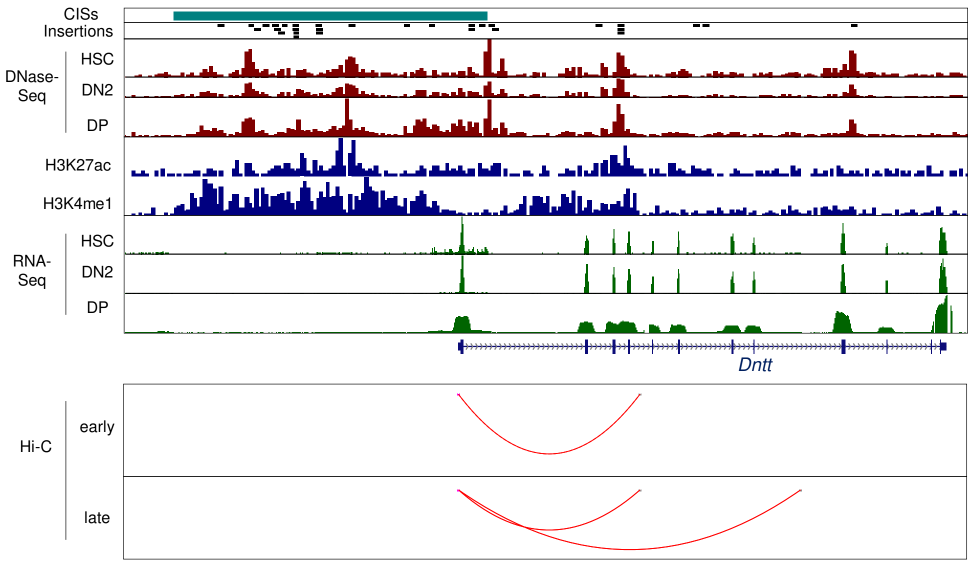

D

Trat1 locus mm10
chr16:48,721,430-48,803,899

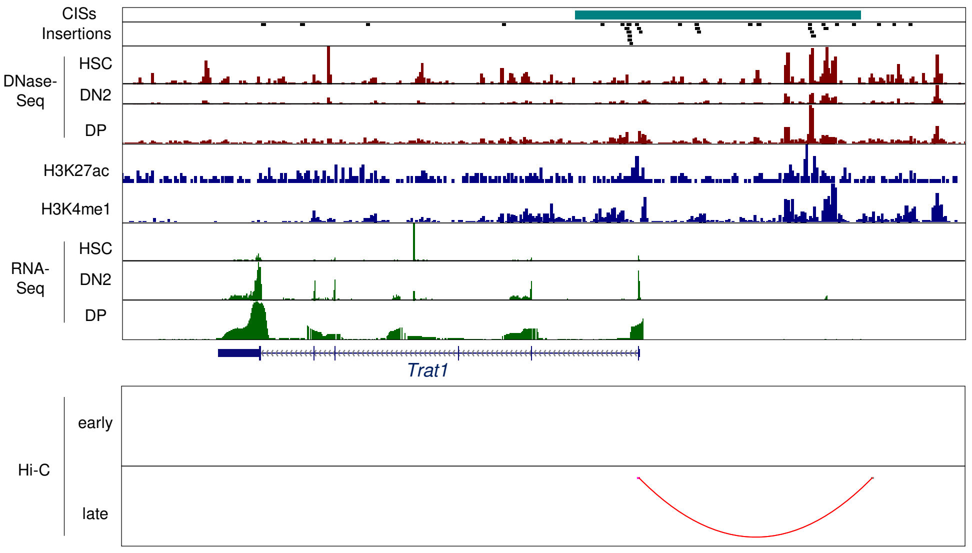

Supplementary Data 4. Visualization of identified regulatory CISs from the group 'PC transcript plus Intergenic Enhancer'. A,-D, UCSC Genome Browser image showing common insertion sites (CISs) and transposon insertions from Rosa26$^{PB/+}$;ATP2 mice. H3K27ac and H3K4me1 tracks from double-positive T cells, as well as DNase-Seq, RNA-Seq and Hi-C data from different stages of T cell evolution are shown (publicly available data as listed in Supplementary Table 5). Protein-coding and non-protein-coding transcripts from the GENCODE gene annotation track are shown. CISs identified in this group overlap with a protein-coding transcript and a putative intergenic enhancer. Related to Figure 2E, Supplementary Table 5.

Supplementary Data 5: Visualization of CISs from ‘PC transcript plus Distant intronic Enhancer’ group
(related to list Figure 2E)

A

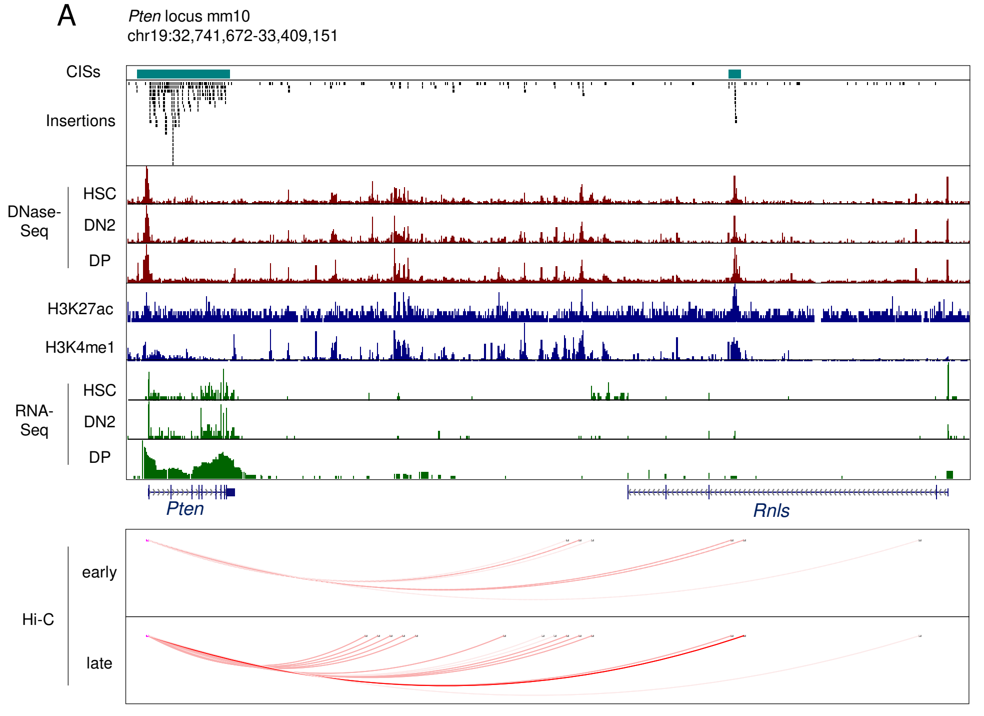

B

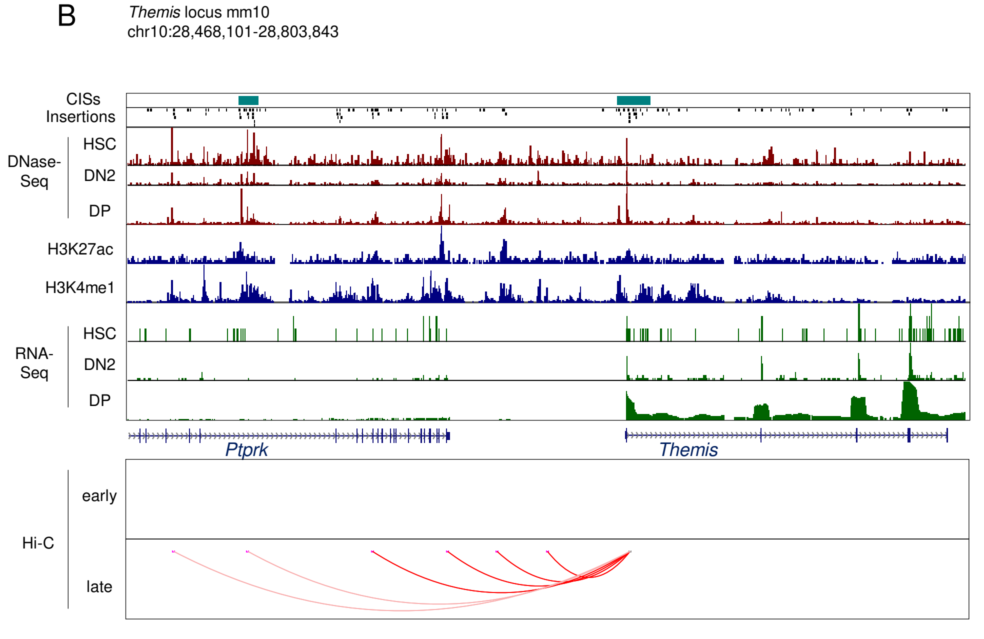

C

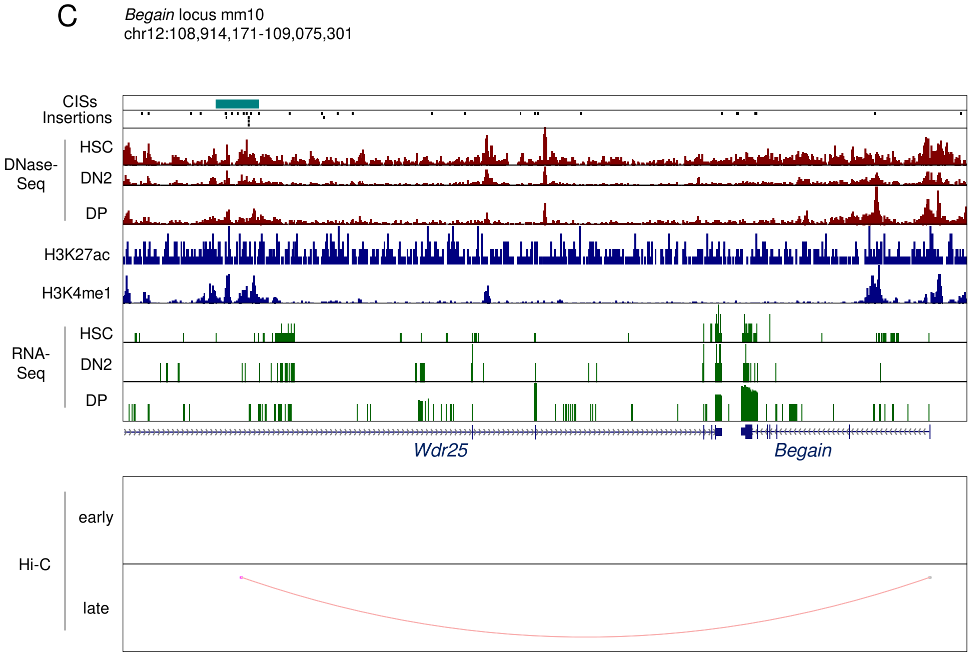
Begain locus mm10
chr12:108,914,171-109,075,301
CISs
Insertions
DNase-Seq
HSC
DN2
DP
H3K27ac
H3K4me1
RNA-Seq
HSC
DN2
DP
Wdr25
Begain
Hi-C
early
late

D

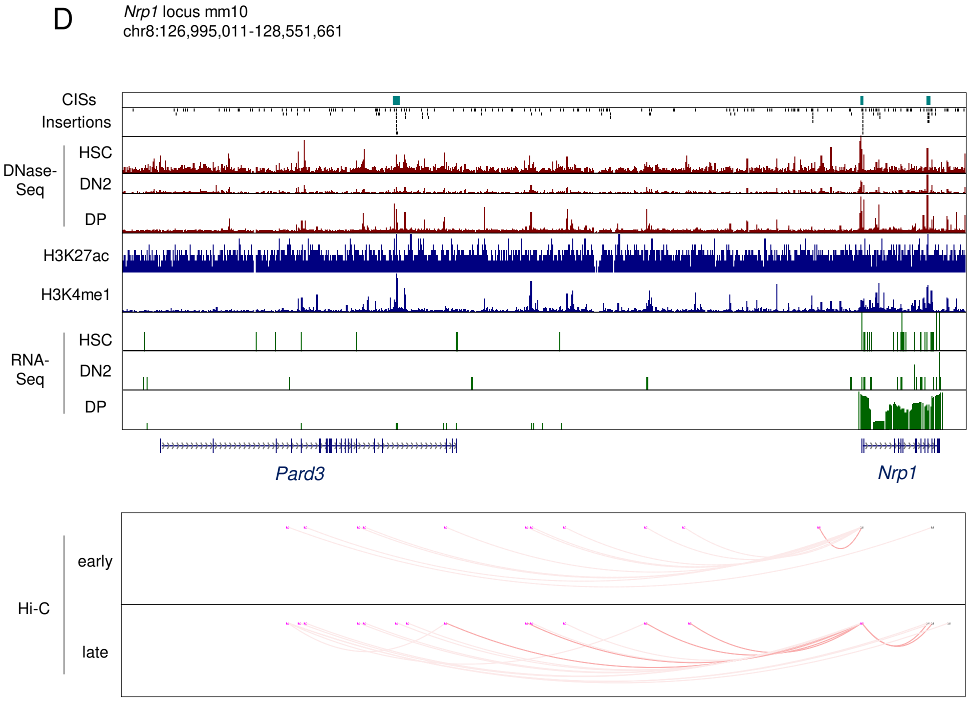
Nrp1 locus mm10
chr8:126,995,011-128,551,661
CISs
Insertions
DNase-Seq
HSC
DN2
DP
H3K27ac
H3K4me1
RNA-Seq
HSC
DN2
DP
Pard3
Nrp1
Hi-C
early
late

E *Cdk19* locus mm10
chr10:40,280,148-40,488,083

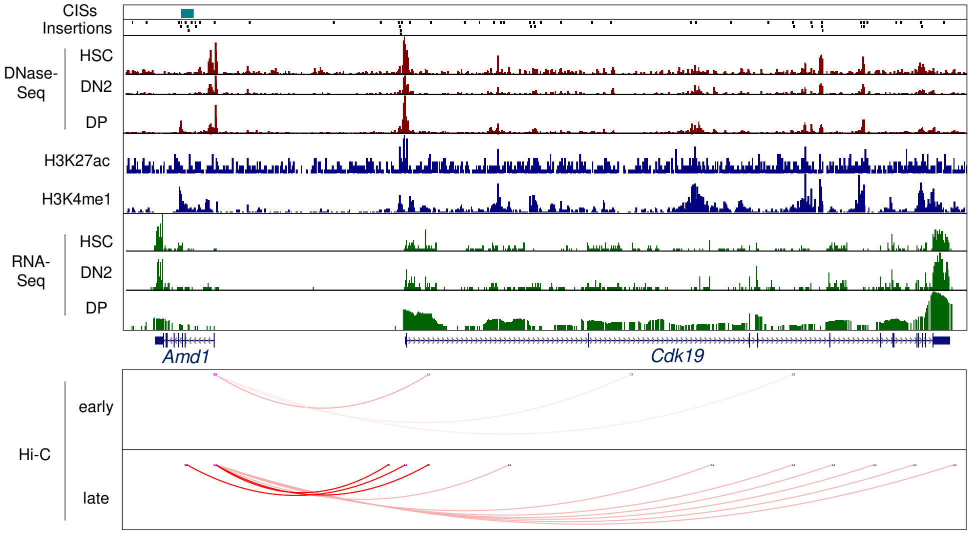

F *Iqgap2* locus mm10
chr13:95,758,235-95,998,464

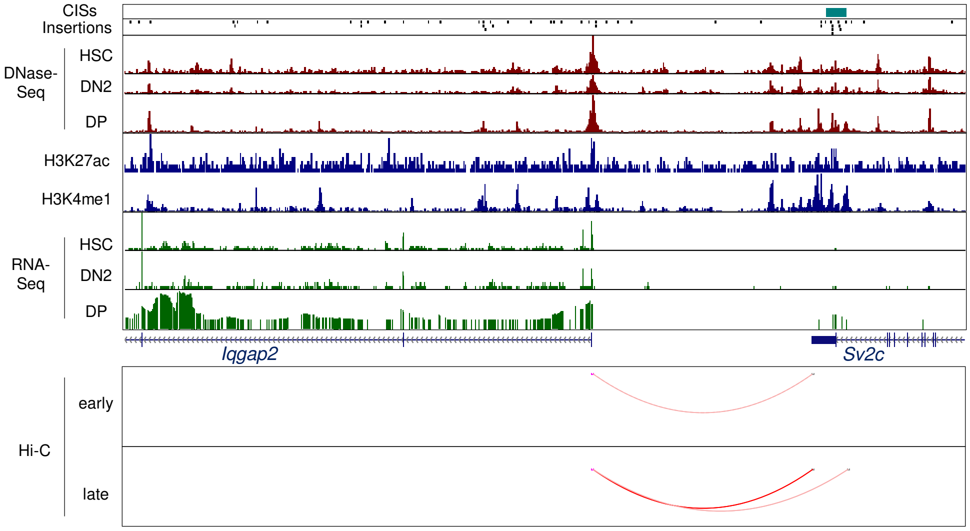

G

Plcxd2 locus mm10
chr16:45,947,383-46,132,367

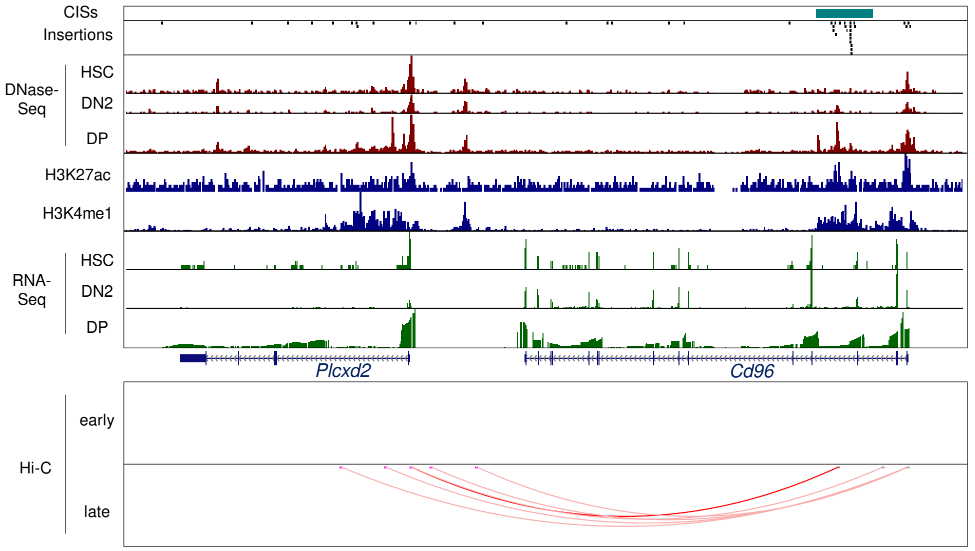

H

Foxo3 locus mm10
chr10:41,793,940-42,287,276

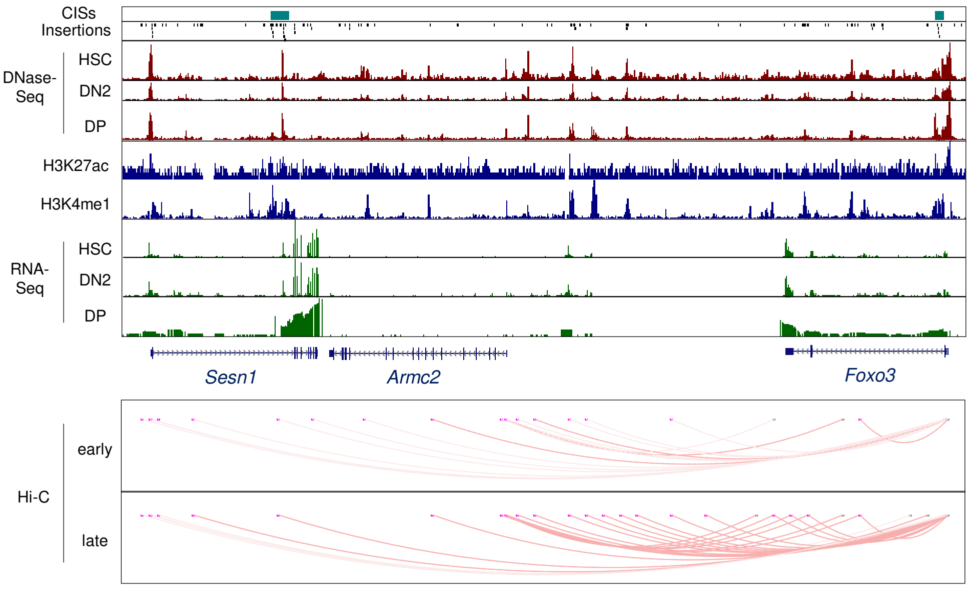

Supplementary Data 5. Visualization of identified regulatory CISs from the group 'PC transcript plus distant intronic Enhancer'. A,-H, UCSC Genome Browser image showing common insertion sites (CISs) and transposon insertions from Rosa26$^{PB/+}$;ATP2 mice. H3K27ac and H3K4me1 tracks from double-positive T cells, as well as DNase-Seq, RNA-Seq and Hi-C data from different stages of T cell evolution are shown (publicly available data as listed in Supplementary Table 5). Protein-coding and non-protein-coding transcripts from the GENCODE gene annotation track are shown. CISs identified in this group overlap with a protein-coding transcript and most likely contain an intronic enhancer element regulating a distant gene. Related to Figure 2E, Supplementary Table 5.

Supplementary References

1. Bailey, M.H., Tokheim, C., Porta-Pardo, E., Sengupta, S., Bertrand, D., Weerasinghe, A., . . . Ding, L. Comprehensive Characterization of Cancer Driver Genes and Mutations. Cell 173, 371-385.e18 (2018).
2. Reddy, A., Zhang, J., Davis, N.S., Moffitt, A.B., Love, C.L., Waldrop, A., . . . Dave, S.S. Genetic and Functional Drivers of Diffuse Large B Cell Lymphoma. Cell 171, 481-494.e15 (2017).
3. Tyner, J.W., Tognon, C.E., Bottomly, D., Wilmot, B., Kurtz, S.E., Savage, S.L., . . . Druker, B.J. Functional genomic landscape of acute myeloid leukaemia. Nature 562, 526-531 (2018).
4. Zhang, J., McCastlain, K., Yoshihara, H., Xu, B., Chang, Y., Churchman, M.L., . . . Mullighan, C.G. Deregulation of DUX4 and ERG in acute lymphoblastic leukemia. Nat Genet 48, 1481-1489 (2016).
5. Pan-cancer analysis of whole genomes. Nature 578, 82-93 (2020).
6. Nguyen, B., Fong, C., Luthra, A., Smith, S.A., DiNatale, R.G., Nandakumar, S., . . . Schultz, N. Genomic characterization of metastatic patterns from prospective clinical sequencing of 25,000 patients. Cell 185, 563-575.e11 (2022).
7. Ing-Simmons, E., Seitan, V.C., Faure, A.J., Flicek, P., Carroll, T., Dekker, J., . . . Merkenschlager, M. Spatial enhancer clustering and regulation of enhancer-proximal genes by cohesin. Genome Res 25, 504-13 (2015).
8. Wei, G., Abraham, B.J., Yagi, R., Jothi, R., Cui, K., Sharma, S., . . . Zhao, K. Genome-wide analyses of transcription factor GATA3-mediated gene regulation in distinct T cell types. Immunity 35, 299-311 (2011).
9. Sidoli, S., Lopes, M., Lund, P.J., Goldman, N., Fasolino, M., Coradin, M., . . . Garcia, B.A. A mass spectrometry-based assay using metabolic labeling to rapidly monitor chromatin accessibility of modified histone proteins. Sci Rep 9, 13613 (2019).
10. Yue, F., Cheng, Y., Breschi, A., Vierstra, J., Wu, W., Ryba, T., . . . Ren, B. A comparative encyclopedia of DNA elements in the mouse genome. Nature 515, 355-64 (2014).
11. Lara-Astiaso, D., Weiner, A., Lorenzo-Vivas, E., Zaretsky, I., Jaitin, D.A., David, E., . . . Amit, I. Immunogenetics. Chromatin state dynamics during blood formation. Science 345, 943-9 (2014).
12. Shih, H.Y., Sciumè, G., Mikami, Y., Guo, L., Sun, H.W., Brooks, S.R., . . . O'Shea, J.J. Developmental Acquisition of Regulomes Underlies Innate Lymphoid Cell Functionality. Cell 165, 1120-1133 (2016).
13. Yoshida, H., Lareau, C.A., Ramirez, R.N., Rose, S.A., Maier, B., Wroblewska, A., . . . Benoist, C. The cis-Regulatory Atlas of the Mouse Immune System. Cell 176, 897-912.e20 (2019).
14. Leong, W.Z., Tan, S.H., Ngoc, P.C.T., Amanda, S., Yam, A.W.Y., Liau, W.S., . . . Sanda, T. ARID5B as a critical downstream target of the TAL1 complex that activates the oncogenic transcriptional program and promotes T-cell leukemogenesis. Genes Dev 31, 2343-2360 (2017).
15. Knoechel, B., Roderick, J.E., Williamson, K.E., Zhu, J., Lohr, J.G., Cotton, M.J., . . . Bernstein, B.E. An epigenetic mechanism of resistance to targeted therapy in T cell acute lymphoblastic leukemia. Nat Genet 46, 364-70 (2014).
16. Michel, B.C., D'Avino, A.R., Cassel, S.H., Mashtalir, N., McKenzie, Z.M., McBride, M.J., . . . Kadoch, C. A non-canonical SWI/SNF complex is a synthetic lethal target in cancers driven by BAF complex perturbation. Nat Cell Biol 20, 1410-1420 (2018).
17. Kwiatkowski, N., Zhang, T., Rahl, P.B., Abraham, B.J., Reddy, J., Ficarro, S.B., . . . Gray, N.S. Targeting transcription regulation in cancer with a covalent CDK7 inhibitor. Nature 511, 616-20 (2014).
18. Manser, M., Sater, M.R., Schmid, C.D., Noreen, F., Murbach, M., Kuster, N., . . . Schär, P. ELF-MF exposure affects the robustness of epigenetic programming during granulopoiesis. Sci Rep 7, 43345 (2017).
19. Lucic, B., Chen, H.C., Kuzman, M., Zorita, E., Wegner, J., Minneker, V., . . . Lusic, M. Spatially clustered loci with multiple enhancers are frequent targets of HIV-1 integration. Nat Commun 10, 4059 (2019).
