# Supplementary material for: In vivo interrogation of regulatory genomes reveals extensive quasi-insufficiency in cancer evolution
Source: Cell Genom. 2023 Mar 8;3(3):100276. doi: 10.1016/j.xgen.2023.100276 (PMC10025556; doi:10.1016/j.xgen.2023.100276)
Supplement: Document S3. Article plus supplemental information [file mmc22.pdf]

Cell Genomics

Article

# *In vivo* interrogation of regulatory genomes reveals extensive quasi-insufficiency in cancer evolution

## Graphical abstract

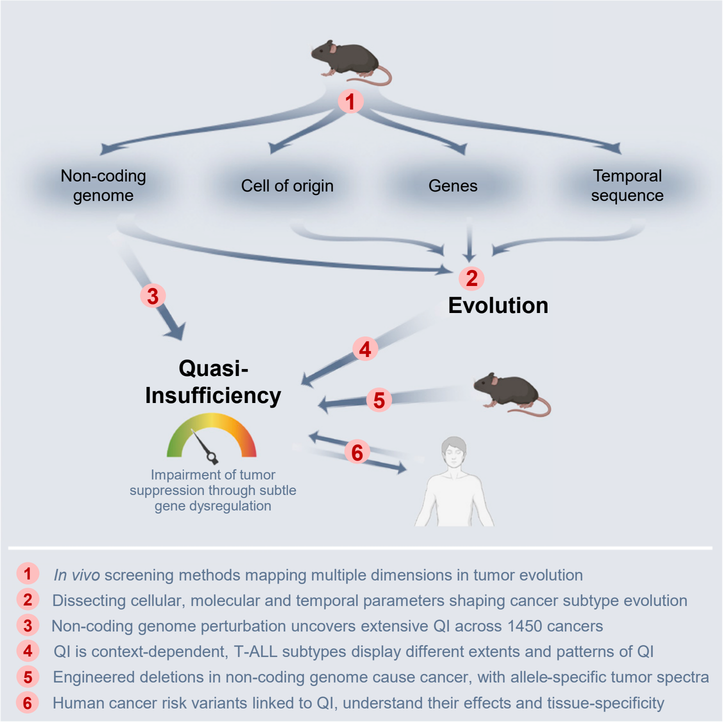


## Highlights

- Methods mapping cellular, molecular, and temporal parameters in cancer evolution
- Non-coding genome perturbation reveals extensive quasi-insufficiency in cancer
- Quasi-insufficiency is context dependent: T-ALL subtype-specific extents/patterns
- Gene desert deletion series causes cancer in mice with allele-specific tumor spectra

## Authors

Anja Fischer, Robert Lersch, Niklas de Andrade Krätzig, ..., Allan Bradley, Lena Rad, Roland Rad

## Correspondence

roland.rad@tum.de

## In brief

Fischer et al. report screening methods interrogating the non-coding genome in mice, which uncovered widespread roles of subtle gene dysregulations in cancer evolution. They show that such quasi-insufficiency is extensive across entities. The discoveries give insights into the impairment of tumor suppression by human cancer risk variants and the tissue-specific effects of non-coding mutations.

Fischer et al., 2023, Cell Genomics *3*, 100276
March 8, 2023 © 2023 The Authors.
https://doi.org/10.1016/j.xgen.2023.100276

Check for updates

CellPress

Cell Genomics

CellPress
OPEN ACCESS

Article

# *In vivo* interrogation of regulatory genomes reveals extensive quasi-insufficiency in cancer evolution

Anja Fischer,[1,2,3,21] Robert Lersch,[1,2,21] Niklas de Andrade Krätzig,[1,2] Alexander Strong,[4] Mathias J. Friedrich,[1,2,5] Julia Weber,[1,2] Thomas Engleitner,[1,2] Rupert Öllinger,[1,2] Hsi-Yu Yen,[3,6] Ursula Kohlhofer,[7] Irene Gonzalez-Menendez,[7] David Sailer,[1,2] Liz Kogan,[1,2] Mari Lahnalampi,[8] Saara Laukkanen,[9] Thorsten Kaltenbacher,[1,2] Christine Klement,[1,2] Majdaddin Rezaei,[1,2] Tim Ammon,[2,10] Juan J. Montero,[1,2] Günter Schneider,[5,11] Julia Mayerle,[12] Mathias Heikenwälder,[13,14] Marc Schmidt-Supprian,[2,3,10] Leticia Quintanilla-Martinez,[7] Katja Steiger,[3,6] Pentao Liu,[4,15] Juan Cadiñanos,[16] George S. Vassiliou,[4,17,18] Dieter Saur,[2,5,19] Olli Lohi,[9] Merja Heinäniemi,[8] Nathalie Conte,[4,22] Allan Bradley,[4,20,22] Lena Rad,[2,19,22] and Roland Rad[1,2,3,5,22,23,*]

[1]Institute of Molecular Oncology and Functional Genomics, School of Medicine, Technische Universität München, 81675 Munich, Germany
[2]Center for Translational Cancer Research (TranslaTUM), School of Medicine, Technische Universität München, 81675 Munich, Germany
[3]German Cancer Consortium (DKTK), Heidelberg, Germany
[4]The Wellcome Trust Sanger Institute, Genome Campus, Hinxton, Cambridge, UK
[5]Department of Medicine II, Klinikum rechts der Isar, School of Medicine, Technische Universität München, 81675 Munich, Germany
[6]Comparative Experimental Pathology, School of Medicine, Technische Universität München, 81675 Munich, Germany
[7]Institute of Pathology and Comprehensive Cancer Center, Eberhard Karls Universität Tübingen, 72076 Tübingen, Germany
[8]Institute of Biomedicine, School of Medicine, University of Eastern Finland, Kuopio, Finland
[9]Faculty of Medicine and Health Technology, Tampere Center for Child, Adolescent and Maternal Health Research and Tays Cancer Center, Tampere University, Tampere, Finland
[10]Institute of Experimental Hematology, TUM School of Medicine, Technical University of Munich, 81675 Munich, Germany
[11]Department of General, Visceral and Pediatric Surgery, University Medical Center Göttingen, 37075 Göttingen, Germany
[12]Medical Department II, University Hospital, LMU Munich, Munich, Germany
[13]German Cancer Research Center (DKFZ), 69120 Heidelberg, Germany
[14]Division of Chronic Inflammation and Cancer, German Cancer Research Center (DKFZ), 69120 Heidelberg, Germany
[15]Li Ka Shing Faculty of Medicine, Stem Cell and Regenerative Medicine Consortium, School of Biomedical Sciences, University of Hong Kong, Hong Kong, China
[16]Instituto de Medicina Oncológica y Molecular de Asturias (IMOMA), 33193 Oviedo, Spain
[17]Wellcome Trust-MRC Stem Cell Institute, Cambridge Biomedical Campus, University of Cambridge, Cambridge CB2 0XY, UK
[18]Department of Haematology, Cambridge University Hospitals NHS Trust, Cambridge CB2 0PT, UK
[19]Institute for Experimental Cancer Therapy, School of Medicine, Technische Universität München, 81675 Munich, Germany
[20]Cambridge Institute of Therapeutic Immunology & Infectious Disease (CITIID), University of Cambridge, Puddicombe Way, Cambridge CB2 0AW, UK
[21]These authors contributed equally
[22]These authors contributed equally
[23]Lead contact
*Correspondence: roland.rad@tum.de
https://doi.org/10.1016/j.xgen.2023.100276

## SUMMARY

In contrast to mono- or biallelic loss of tumor-suppressor function, effects of discrete gene dysregulations, as caused by non-coding (epi)genome alterations, are poorly understood. Here, by perturbing the regulatory genome in mice, we uncover pervasive roles of subtle gene expression variation in cancer evolution. Genome-wide screens characterizing 1,450 tumors revealed that such quasi-insufficiency is extensive across entities and displays diverse context dependencies, such as distinct cell-of-origin associations in T-ALL subtypes. We compile catalogs of non-coding regions linked to quasi-insufficiency, show their enrichment with human cancer risk variants, and provide functional insights by engineering regulatory alterations in mice. As such, kilo-/megabase deletions in a *Bcl11b*-linked non-coding region triggered aggressive malignancies, with allele-specific tumor spectra reflecting gradual gene dysregulations through modular and cell-type-specific enhancer activities. Our study constitutes a first survey toward a systems-level understanding of quasi-insufficiency in cancer and gives multifaceted insights into tumor evolution and the tissue-specific effects of non-coding mutations.

Check for updates


This is an open access article under the CC BY license (http://creativecommons.org/licenses/by/4.0/).

## INTRODUCTION

Cancer evolution is driven by altered cellular signaling states, resulting from structural genome changes or dysregulated gene expression.[1,2] Early work on hereditary cancer syndromes, such as retinoblastoma,[3,4] linked oncogenesis to biallelic loss of tumor suppressor genes (TSGs). Experimental proof for this two-hit hypothesis was provided by the first TSG knockout mice,[4–6] while subsequent studies uncovered that, for some TSGs, inactivation of one allele is sufficient to promote oncogenesis.[7,8] This phenomenon, referred to as haploinsufficiency, can be obligate and often displays context dependencies.[9–12] Even more pronounced TSG dosage sensitivity became evident in studies analyzing hypomorphic *Pten* alleles, which showed that very minor variation of gene expression can lead to impaired tumor suppression—a state termed TSG quasi-insufficiency.[13,14] In analogy to TSGs, transformation induced by oncogenes often relies on an optimal dosage, which varies depending on the cellular or co-mutational context.[10,15–20] While this dosage-dependent continuum model of cancer gene function is documented for few genes,[14] scalable methods to systematically map and causally connect subtle gene dysregulations with cancer development in organisms are largely missing.

Subtle dysregulation affects thousands of genes in a cell and can result from interference with regulatory elements (REs). The protein-coding exome is 50 times smaller than the non-protein-coding (nPC) genomic space, of which a considerable part is thought to constitute regulatory sequence.[21] During oncogenesis, the regulatory genome undergoes extensive changes, either through structural alterations (such as somatic mutations or copy-number variation) or adaptive processes (such as global chromatin remodeling through cell-intrinsic and -extrinsic triggers).[21,22] However, functional annotation of cancer-causing non-coding regulatory alterations, their combinatorial effects, and cell-type-specific functions remains a major challenge.[23] Likewise, while up to 90% of the genome is transcribed[24] (of which only a smaller part encodes for mRNAs) global functional interrogation of non-coding RNAs (ncRNAs) in cancer is in its infancy.

Genomic alterations in the nPC cancer genome are frequent, but their functional relevance is largely unexplored.[21] T-ALL is a prominent example for a disease characterized by low numbers of mutations in PC sequence (on average 6 per tumor),[25] but almost 1,000 in the nPC genome.[26] The effects of these non-coding mutations are not understood, barring few examples,[27–31] but could indicate a possible role of quasi-insufficiency in T-ALL evolution. Human T-ALL is a heterogeneous disease. The latest WHO classification added early T cell precursor ALL (ETP-ALL, which develops from immature T cells) as a biologically distinct—but in itself heterogeneous—sub-entity with poor prognosis.[32–34] The molecular principles shaping sequential evolution of different T-ALL subtypes is, however, not well understood.

The use of transposon systems for insertional mutagenesis in mice[35–37] made important contributions to the census of cancer genes.[20] Such screens proved particularly powerful in the discovery of drivers that are typically not mutated in human cancer but dysregulated by other means—and are hence difficult to identify by genome-sequencing approaches. Transposon insertions can also affect REs,[20,38] thereby likely causing subtle gene dysregulations. Here, we exploited insertional mutagenesis for systematic functional interrogation of the regulatory genome. We developed screening and analytical approaches, which allowed us to perform genome-wide surveys for quasi-insufficiency in solid and hematopoietic cancers. We also devise forward-directed screening approaches to interrogate *in vivo* cancer evolution. Using T-ALL as a model, these screens show how combinatorial codes of molecular, cellular, and temporal parameters dictate tumor subtype evolution, and highlight extensive quasi-insufficiency, which displays marked context dependencies, including cell-of-origin associations.

## RESULTS

### *In vivo* interrogation of the coding and non-coding genome using T-ALL as a model entity

We previously developed *PiggyBac* screening systems for gene discovery in mice.[20,37–39] We now set out to develop methods for systematic exploration of the nPC genome. Whole-body mutagenesis using the *PiggyBac* transposase and ATP2 type transposons induces tumorigenesis in the B, T, or myeloid lineage[37] (Figure S1). To allow subtype-specific analyses, we generated a large cohort (n = 256) of *Rosa26*$^{PB/+}$*;ATP2* mice, which we monitored for cancer development (Figure S1; Table S1). Tumors were characterized using immunohistochemistry and T cell (acute) lymphoblastic lymphoma/leukemia (T-LBL/T-ALL; hereafter referred to as T-ALL; n = 51) was used as a model to investigate quasi-insufficiency in cancer (Figures S1C and S2A).

Quantitative insertion site sequencing (QiSeq)[40] of all T cell tumors revealed 170,075 non-redundant transposon integrations (Figure S2B). To map genomic regions affected by transposon insertions more significantly than expected by chance, we performed statistical analyses based on Gaussian kernel convolution (GKC).[41] Using CIMPL (common insertion site mapping platform), we identified 1,062 common insertion sites (CISs), of which 994 CISs were found in at least 10% of samples.

Figure 1A displays the top 50 CIS genes, including: (1) known T-ALL drivers (such as *Notch1*, *Pten*, or *Bcl11b*),[42] (2) genes that have not been linked to T-ALL before, but to other hematologic malignancies (e.g., *Cux1*, *Mecom*, *Crebbp*), and (3) genes that have not yet been associated with hematopoietic cancers so far. Although the latter are typically poorly studied, some have been linked to signaling (*Sh3kbp1*, *Sipa1l1*) or immune functions (*Slamf6*, *Ly6e*, *Mgat5*). Moreover, we found that several of these genes are strongly regulated during T cell development (*Gfra1*, *Nck2*, *Prim2*, *Serbp1*, *Fam169b*) (Figure S3), indicating a function in the T cell lineage. The full list of CISs and information on known association to human cancer is provided in Tables S2 and S3.

To examine the suitability of our screening system for interrogation of the nPC genome, we first assessed general characteristics of *PiggyBac* transposition. By examining the global distribution of insertions we found that nearly half are located in intergenic regions (Figure 1B). This is comparable with our hematopoietic screens performed with *Sleeping Beauty* (55% of insertions), a transposon system that does not have insertion

CellPress
OPEN ACCESS

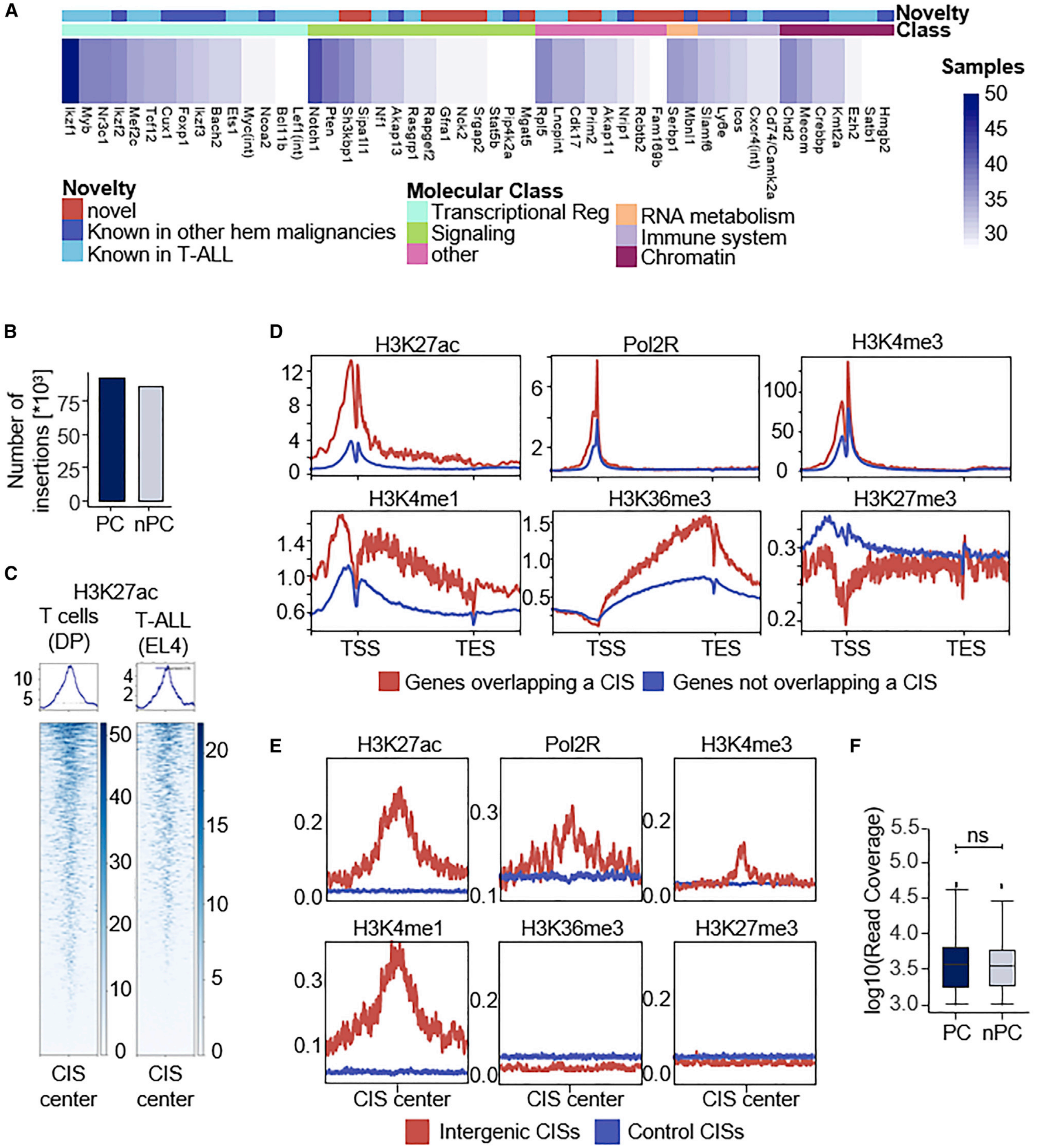


**Figure 1. A genome-wide *PiggyBac* transposon screen interrogating the coding and non-coding genome in T-ALL**

(A) Top 50 CISs classified by molecular category (as in Liu et al.[42]) and novelty. The heatmap indicates the number of samples with insertions in the respective CIS.

(B) Number of unique insertions in the protein-coding and non-protein-coding genome. Protein-coding includes exonic and intronic sequence.

(C) Profile heatmap plot showing overlap of CIS regions (n = 1,062) with H3K27ac peaks in T cells (DP stage[43]) and the lymphoblastic T cell line EL4.[44] 10 kb in both directions around the CIS center are shown.

(D) Profile plots of thymus ChIP-seq data at genes with (n = 914, red) and without (n = 20,935, blue) CIS overlap. A region 2 kb upstream of the transcriptional start site (TSS) and 2 kb downstream of the transcriptional end site (TES) is shown.

*(legend continued on next page)*

biases toward intragenic insertions. This indicates that selection rather than integration preference is the source of non-coding CISs, thus supporting the functional relevance of regulatory regions in tumorigenesis.

Next, we compared *PiggyBac* insertion profiles with epigenetic features in the T cell lineage and investigated differences between the PC (the sum of exonic and intronic sequence; approximately 25% of the genome) and nPC genome. The overlay of all CIS regions with H3K27ac enhancer histone marks in healthy and malignant T cells revealed enrichment of active chromatin in CISs (Figure 1C). Looking specifically at the PC genome, we found—as expected—a substantial accumulation of active chromatin marks and depletion of repressive marks at CIS-overlapping genes (Figure 1D) compared with genes not overlapping with CISs. Notably, the enrichment of active chromatin marks in CISs is also true for the nPC genome (Figures 1E and S4). Thus, beyond its preference for transcribed genes,[45–48] *PiggyBac* has a general propensity for active chromatin, supporting its application to perturb cancer-relevant REs.

Finally, because cancer driver insertions are more likely to support clonal outgrowth than passenger insertions, we examined sequencing read coverage profiles of genic and intergenic insertions. We found no major differences between these groups (Figure 1F), suggesting comparable functional relevance of genic and regulatory CISs.

## Annotation of epigenetic features in nPC CISs

For GKC statistics, commonly used scale parameters to identify protein-coding CISs range between 30 and 240 kb. Because the average size of REs (1.5 kb) is smaller than of PC genes (8 kb), we speculated that the scale parameter needs to be adjusted. Systematic comparison of different CIS window sizes used for GKC analyses indeed revealed that reducing the scale parameter to 5k increases the sensitivity of regulatory CIS discovery (Figures 2A, 2B, and S5; Table S4).

To annotate functional properties of intergenic CISs, we developed ARCIS (annotation pipeline for regulatory common insertion sites) (Figures 2C, S5, and S6; see STAR Methods for details), which we used to overlap CIS regions with epigenetic data from different T cell developmental stages or T-ALL (chromatin accessibility, histone modifications, and information on 3D organization,[43] Tables S5 and S6). The ARCIS output supports fast explorative analyses by allowing one to (1) rank CISs according to their regulatory potential (RE score), (2) sort for an RE category of interest, and (3) search for an RE target gene of interest (Tables S7 and S8). We also developed rules for final RE assignment based on manual inspection of ARCIS output data (Figure S6). Overall, the analyses created a catalog of cancer-relevant REs in T-ALL. Specific results are shown for the 45 high-scoring REs in Figure 2E, which indicates for each CIS the related RE category and target gene/transcript. Data S1–S5 provide detailed visualizations of all related genomic regions.

To explore the human relevance of identified REs, we examined the regulatory activity of CIS syntenic human regions. To this end, we performed lift-over of mouse CIS coordinates to the human genome followed by annotation of a range of epigenomic human data (Figures 2C, 2D, and S7; Table S9). Subsequent cross-species analyses revealed that the syntenic human regions of mouse regulatory CISs display high concordance in their regulatory activity (Figure 2D).

## Perturbation of regulatory CISs causes subtle dysregulations of target gene expression

We first examined *inter*genic REs and assigned potentially linked genes (Figure 2E; Data S1; Table S8). Beyond known T-ALL drivers (such as *Runx1*, *Lef1*, *Bcl11b*, and *Rasgrp1*), this list includes genes with a role in T cell biology (*Satb1* and *Rag2*) as well as developmental genes for which a function in T cells has not been described before (such as *Sall3* and the *Hoxd* cluster). In principle, transposon insertions in regulatory regions can positively or negatively affect expression of target genes. Possible mechanisms include disruption of transcription factor binding sites, interference with 3D chromosomal conformation, and topology-associated domain structure (see Table S3 for details on the putative cancer relevance of target genes).

We next examined *intra*genic (intronic) REs, which are difficult to identify in screens, as common analytical approaches assign CISs primarily to overlapping genes. We therefore exploited 3D connectivity data[43] to assign intronic REs to their putative distant target genes. These analyses identified 30 CISs categorized as intronic REs (Data S5). Their main characteristics are: (1) clustered insertion peak in a narrow intronic area, (2) unbiased transposon orientation, (3) Hi-C connection to a distant gene, and (4) often absent CIS gene expression in the relevant tissue. Examples of genes regulated by newly identified REs include *Pten* (a known T-ALL tumor suppressor[49,50]), *Themis* and *Nrp1* (not implicated in T-ALL so far, but in T cell biology[51,52]), or *Txn1* and *Iqgap2* (not studied in T cells so far).

The validity of the screen is exemplified by a narrow intronic CIS region in *Rnls*, which has a Hi-C connection to the ~400 kb distant *Pten* promoter (Figure 3A) and was recently described as a *Pten* enhancer.[53] Using global run-on sequencing (GRO-seq), we examined the relevance of this RE in human T-ALL patient data (Figure 3B) and found cell-type-specific enhancer activity, with enhancer RNA signal peaks being present in T-ALL patients but not in HEK293T cells. Accordingly, CRISPR-Cas9-based deletion of the 7–8 kb RE region led to a stronger decrease of *PTEN* expression in human and murine T cells (34% and 24% reduction) than in HEK293 cells (15% reduction) (Figure 3C).

We identified CISs affecting 54 nPC transcripts (Figure 2E; Data S2; Table S8). More than 70% of these ncRNAs are expressed during T cell development (Figure S5D). Several are in proximity to known T-ALL genes, such as *Myb*, *Myc*, and *Ptprc*.[54–56] Others are potential regulators of transcription factors and signaling genes, such as *Fam126a*, *Il6st*, and

(E) Profile plots of thymus ChIP-seq data at intergenic CIS regions (n = 227, red) and control CISs regions (blue, see STAR Methods). A region of 10 kb upstream and 10 kb downstream of the CIS center is shown.

(F) Read coverage of protein-coding and non-protein-coding insertions (insertions >1,000 reads included; p = 0.45, Wilcoxon test). hem, hematological; PC, protein coding; nPC, non-protein coding; DP, double positive; CIS, common insertion site.

CellPress
OPEN ACCESS

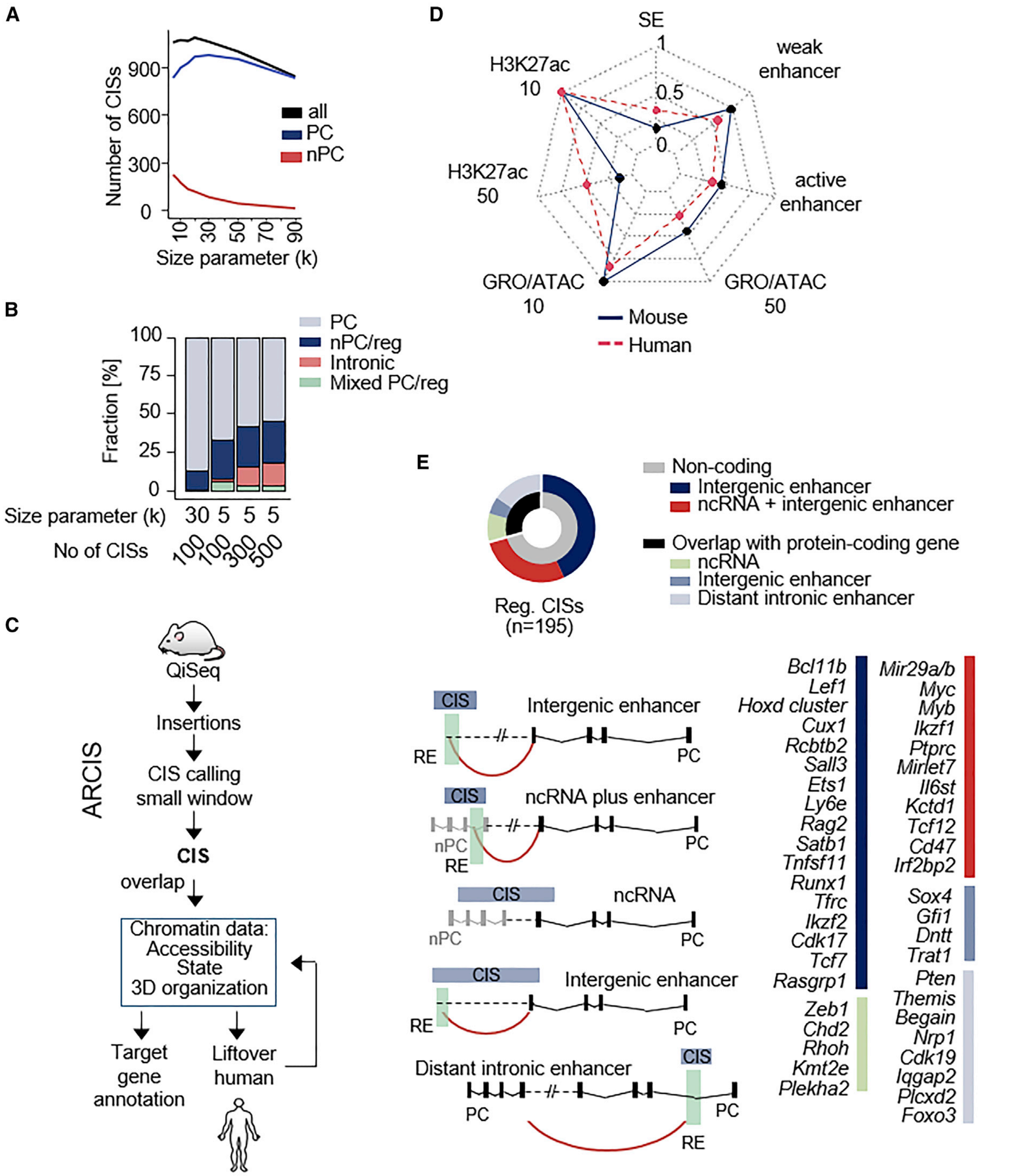


**Figure 2. New methods support systematic identification and annotation of regulatory CISs**

(A) Number of CISs resulting from CIMPL analysis using different size parameters (5–90k).

(B) Percentage of regulatory CISs dependent on the size parameter used and number of CISs analyzed (top 100, 300, or 500).

(C) Schematic representation of the ARCIS framework to annotate putative regulatory elements in CIS regions.

(D) Radar chart showing percentage of epigenomic features overlapping with mouse CISs and CIS syntenic human regions. The annotation of weak and active enhancers were derived from chromHMM models, of super-enhancers (SE) from dbSuper. For H3K27ac ChIP-seq, GRO-seq, and ATAC-seq different read cutoffs (either 10 or 50 reads) were used.

(E) Representation of different CIS categories. Schemes for all categories are shown. Red lines indicate HiC connections. All CIS regions above an arbitrary set threshold (found in at least 7 samples, n = 537) were annotated. 195 regulatory CISs were identified using the ARCIS framework and manually verified to identify high-confidence regions for each category. The potential target gene of each regulatory region is listed on the right for the top 45 regulatory CISs. A detailed description of the analytical procedure can be found in Figure S6. PC, protein coding; nPC, non-protein coding; reg, regulatory; RE, regulatory element.

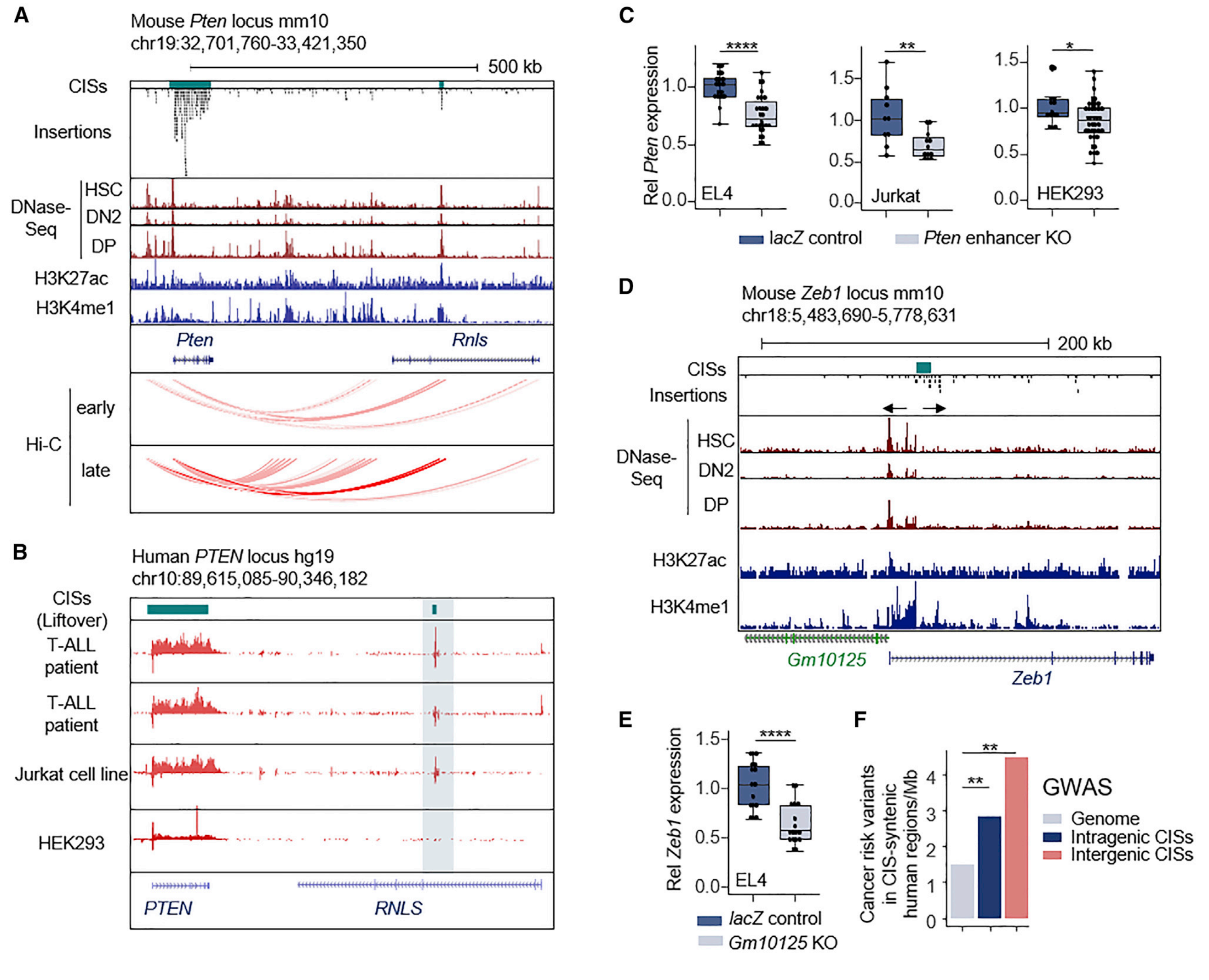


**Figure 3. Functional validation of regulatory CISs**

(A) Insertions and CISs in the murine *Pten* and *Rnls* gene locus. H3K27ac and H3K4me1 tracks from double-positive T cells, as well as DNase-seq and Hi-C data from different stages of T cell evolution (early, HSC-DN2a; late, DN2b-DN3) are shown below (publicly available data as listed in Table S5). The intronic CIS region in the *Rnls* gene shows overlap with active chromatin and a Hi-C link to the *Pten* promoter.

(B) Human *PTEN* locus. Indicated are the CIS-syntenic human regions (top, green) and GRO-seq tracks (red) of two T-ALL patients, the Jurkat and HEK293 cell lines. The syntenic region of the narrow regulatory CISs shows a typical bidirectional enhancer RNA GRO-seq signal peak in T-ALL patients and in Jurkat cells. Of note, *RNLS* is not expressed in T-ALL, supporting the notion that the CIS target is not *Rnls* itself, but its intronic RE.

(C) *Pten* expression in clones with/without CRISPR-Cas9-based knockout of the potential *Pten* enhancer (~7 kb) located in the *Rnls* gene. Each dot represents relative *Pten* gene expression in a single-cell-derived clone normalized to *Gapdh* expression. Experiments are shown for cell lines EL4 (KO n = 26, 8/26 homozygous, ctrl n = 18), Jurkat (human T-ALL; KO n = 12, 0/12 homozygous, ctrl n = 10), and HEK293 (KO n = 38, 6/38 homozygous, ctrl n = 14).

(D) Murine chr18 region encompassing *Zeb1* and the *Zeb1* antisense transcript *Gm10125*. Arrows indicate the orientation of insertions peaks.

(E) *Zeb1* expression in clones with/without CRISPR-Cas9-based knockout of *Gm10125* exons 2 and 3 (~2 kb) in EL4 cells (KO n = 17, 9/17 homozygous, ctrl n = 16; deletion boundaries 8 kb upstream of the *Zeb1* promoter). Each dot represents relative *Zeb1* gene expression in a single-cell-derived clone normalized to *Gapdh* expression. *$p < 0.05$, **$p < 0.01$, ***$p < 0.001$, ****$p < 0.0001$, Wilcoxon test.

(F) Number of cancer-associated GWAS variants in CIS-syntenic human regions. Cancer-risk variants were filtered from the NHGRI-EBI GWAS catalog and pruned for linkage disequilibrium. Variants were overlapped with CIS syntenic human coordinates. The sum of all CIS sizes (width) was used for statistical calculation. **$p < 0.01$, $\chi^2$ test. CIS, common insertion site; HSCs, hematopoietic stem cells; DN2, double-negative stage 2; DP, double-positive stage; Rel, relative. EL4, mouse T lymphoblastic cell line; Jurkat, human T-ALL cell line; HEK293, immortalized human embryonic kidney cells.

*Kctd1*, which have so far not been implicated in T-ALL (Data S2). We performed detailed studies on a CIS overlapping with *Zeb1*, which was annotated as "PC transcript plus ncRNA" by ARCIS. Manual inspection of insertion patterns revealed two peaks with opposite transposon orientations, predicted to activate either *Zeb1* or the *Zeb1* antisense transcript *Gm10125* (Figure 3D). Human *ZEB1-AS* RNA can activate *ZEB1* expression through recruitment of H3K4 methyltransferases.[57] Accordingly, we

observed decreased *Zeb1* expression (39% reduction) upon heterozygous *Zeb1*-AS deletion in mouse EL4 cells (Figure 3E). Thus, the functional outcome of both insertion clusters is induction of *Zeb1* expression. Of note, *Zeb1* was shown to have tumor-suppressive function in T cells: *Zeb1* knockout mice develop mature (classical) T-ALL.[58] In our screen, however, *Zeb1* insertions were (1) enriched in immature T-ALL and (2) predicted to be oncogenic, as in AML.[59] These results therefore suggest a dual role of *Zeb1* in T-ALL, depending on cellular context (before or after T cell commitment).

We also validated regulatory CISs affecting other loci, including *Ikzf1* (Figures S8A–S8C) or *Lncpnt/mir29* (see details in Figures S8D–S8F). The leukemia-associated transcription factor *Ikzf1* was marked by two CISs (Figure S8A). One CIS overlaps with *Ikzf1* itself and displays the expected gene inactivation-type insertion pattern, consistent with the known tumor-suppressive function of *Ikzf1* (Figure S8B). The second CIS overlaps with a region 100 kb upstream of *Ikzf1* harboring enhancer sequence as well as a lncRNA (*Gm11998*), which is subject to regulation during T cell development (Figure S8A). Deleting this region in T-ALL cell lines caused subtle but significant effects on *Ikzf1* expression (32% reduction, Figure S8C), confirming the predictions of the screen.

Taken together, these data show that nPC insertions are functional and exert subtle effects on target gene expression. Their large number suggests extensive quasi-insufficiency in T-ALL.

## nPC CISs are enriched with human cancer risk variants

Cancer risk variants identified in human genome-wide association studies (GWASs) frequently affect non-coding sequence, suggesting subtle gene regulatory effects. To explore a possible link between putative human and mouse regulatory alterations, we intersected human GWAS and our regulatory CIS lists. We indeed found that regulatory CIS targets (n = 149 genes) were highly significantly enriched for GWAS-associated human cancer variants ($p = 0.001$, pan-cancer variants; $p = 2.98 \times 10^{-6}$ hematopoietic cancer variants; $\chi^2$ test; Table S10).

In an orthogonal approach we performed lift-over of mouse CISs coordinates to the human genome and used the syntenic human regions to analyze their overlap with cancer-associated GWAS variants (Table S11). To exclude SNPs located on the same haplotype block, the list of GWAS variants was pruned for linkage disequilibrium using LDlink.[60] We found an enrichment of pan-cancer GWAS risk variants in human genomic regions syntenic to mouse CISs (3.09 variants per Mb) as compared with their overall frequency in the human genome (1.48 pan-cancer GWAS risk variants per Mb; $p = 2.3 \times 10^{-5}$, $\chi^2$ test; Table S11). This enrichment was more pronounced for human regions syntenic to mouse intergenic CISs (4.48 variants per Mb; $p = 0.0029$; $\chi^2$ test) as compared with regions syntenic to intragenic CISs (2.82 variants per Mb; $p = 0.001$; $\chi^2$ test, Figure 3F). These results support the human relevance of the screens.

## Gene desert deletions in mice drive oncogenesis through subtle regulatory effects

Statistically, it is extremely unlikely for transposon insertions to occur on both alleles of a gene or regulatory region in the same cell. We therefore assume that interference with RE function in our screen is largely mono-allelic. This suggests that even very subtle interference with gene regulation can promote malignant transformation. To date there is, however, little evidence that this assumption holds true in mouse cancer models.

The most common pediatric T-ALL translocation is t(5;14)(q35;q32), fusing a gene desert (a genomic region without protein-coding genes) downstream of *BCL11B* to *TLX3* (20%–25% pediatric, 5% adult cases), or more rarely to *NKX2-5* or *ZEB2*.[61] Thereby, hijacking of *BCL11B* REs leads to overexpression of these translocation partners, which has been shown to be oncogenic.[62,63] However, it is unclear whether mono-allelic enhancer de-commissioning in itself is sufficient to induce tumors in organisms (for example, through reduced *BCL11B* expression). To address this question, we first explored the syntenic mouse region in our screen, which revealed several CISs in the gene desert downstream of *Bcl11b*, suggesting that interference with *Bcl11b* enhancers can indeed in itself be oncogenic (mice do not have translocations).

The translocation breakpoints in human T-ALL are almost exclusively located downstream *BCL11B*. This ~1 Mb region displays regulatory activity,[64,65] which we confirmed at high resolution by GRO-seq in human T-ALL (Figure 4A). The syntenic mouse regulatory region was marked by several independent CISs (Figure 4B). Moreover, there are multiple physical interactions of CIS-marked putative REs with the *Bcl11b* promoter in the T cell lineage (Figure 4B).

Guided by the human translocation coordinates (and the mouse CISs locations), we engineered two mouse models with kilo- to megabase-scale germline deletions ($Bcl11b^{\Delta 105kb}$, n = 148 and $Bcl11b^{\Delta 1Mb}$, n = 49) in the gene desert with regulatory activity downstream of *Bcl11b* (Figure 4B). We found that not only biallelic deletion but also heterozygous knockout mice displayed reduced *Bcl11b* expression in healthy tissues, although the effects were very subtle for the smaller mutant $Bcl11b^{\Delta 105kb}$ (Figure 4C). In both cohorts, animals started to develop signs of sickness at a young age (Table S12). A subset of animals developed symptoms reminiscent of neurodevelopmental phenotypes (Figure S9A), such as tremor, consistent with a function of *Bcl11b* in brain development.[66]

The second major phenotype was cancer. Overall, 22%–45% of animals developed tumors (Figure 4D), while none of the animals in the wild-type cohort (n = 21) developed cancer. These numbers even underestimate the oncogenic effect of the knockouts, considering that a large subset of animals had to be sacrificed at a young age because of neurodevelopmental phenotypes. The tumor spectrum comprised hematologic cancers, including T and B cell malignancies as well as a range of solid cancers (Figures 4E, S9B, and S10; Table S12). Of note, although all genotypes displayed highly penetrant cancer phenotypes, tumor onset differed substantially between groups: median tumor-related survival was lowest in $Bcl11b^{\Delta 1Mb/\Delta 1Mb}$ mice (195 days), followed by $Bcl11b^{\Delta 105kb/\Delta 105kb}$ (338 days), $Bcl11b^{\Delta 1Mb/WT}$ (466 days), and $Bcl11b^{\Delta 105kb/WT}$ (640 days) mice (Figure 4F).

We next examined whether individual genotypes give rise to different cancer phenotypes and found strongly biased representation for T-ALL (Figures 4E and 4G). $Bcl11b^{\Delta 1Mb/\Delta 1Mb}$ mice

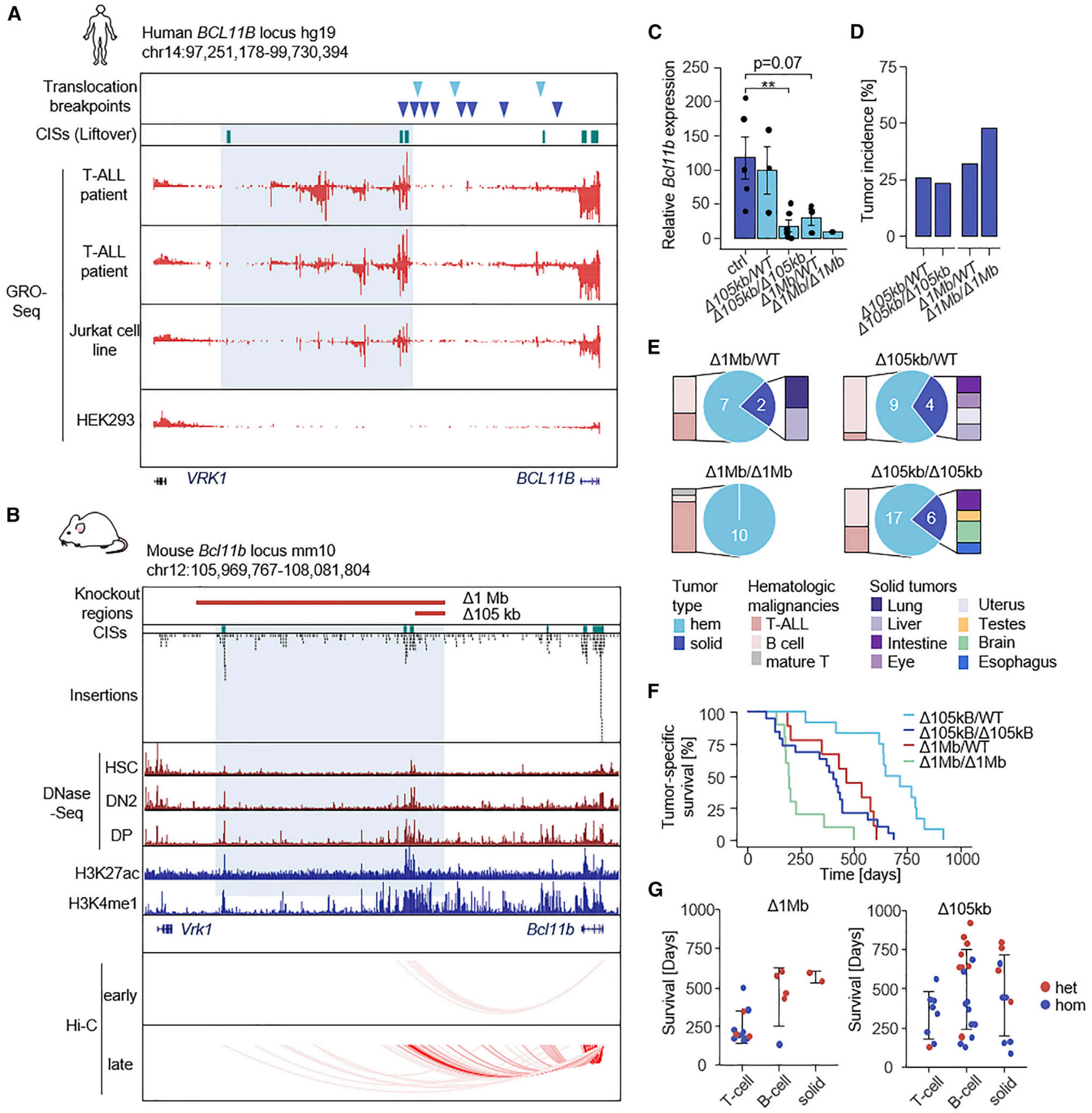

**Figure 4. Allelic series of "gene desert" deletions in mice display gradual gene dysregulations and differential cancer phenotypes**

(A) Human *BCL11B* locus. Indicated are translocation breakpoints detected in T-ALL patients (dark blue) and cell lines (light blue), GRO-seq tracks of two T-ALL patients, the Jurkat and HEK293 cell lines. A region with putative high regulatory activity is highlighted. CIS-syntenic regions are indicated in green.

(B) Mouse *Bcl11b* locus. Engineered intergenic germline deletions (as informed by CIS location and human translocation breakpoints) are indicated by red bars. H3K27ac and H3K4me1 tracks from double-positive T cells, as well as DNase-seq and Hi-C data from different stages of T cell evolution (early, HSC-DN2a; late, DN2b-DN3) are shown (publicly available data as listed in Table S5).

(C) *Bcl11b* expression in thymi of healthy (no tumor) wild-type and knockout mice (ctrl, n = 5; 105 kb-het, n = 3; 105 kb-hom, n = 6; 1 Mb-het, n = 3; 1 Mb-hom, n = 1). qPCR was performed in duplicate and gene expression was normalized to *Gapdh.* Data are presented as mean ± SEM (**p < 0.01, Wilcoxon test).

(D) Incidence of tumors in 105 kb (36/148) and 1 Mb (19/49) knockout mice.

(E) Tumor spectra of 105 kb and 1 Mb knockout mice.

(F) Cancer-specific survival of 1 Mb and 105 kb knockout mice.

(G) Tumor-type-specific survival of 1 Mb and 105 kb knockout mice. Error bars represent SD. CIS, common insertion site; HSC, hematopoietic stem cell; DN, double-negative stage; DP, double-positive stage; WT, wild type; hem, hematopoietic; het, heterozygous; hom, homozygous; ctrl, control.

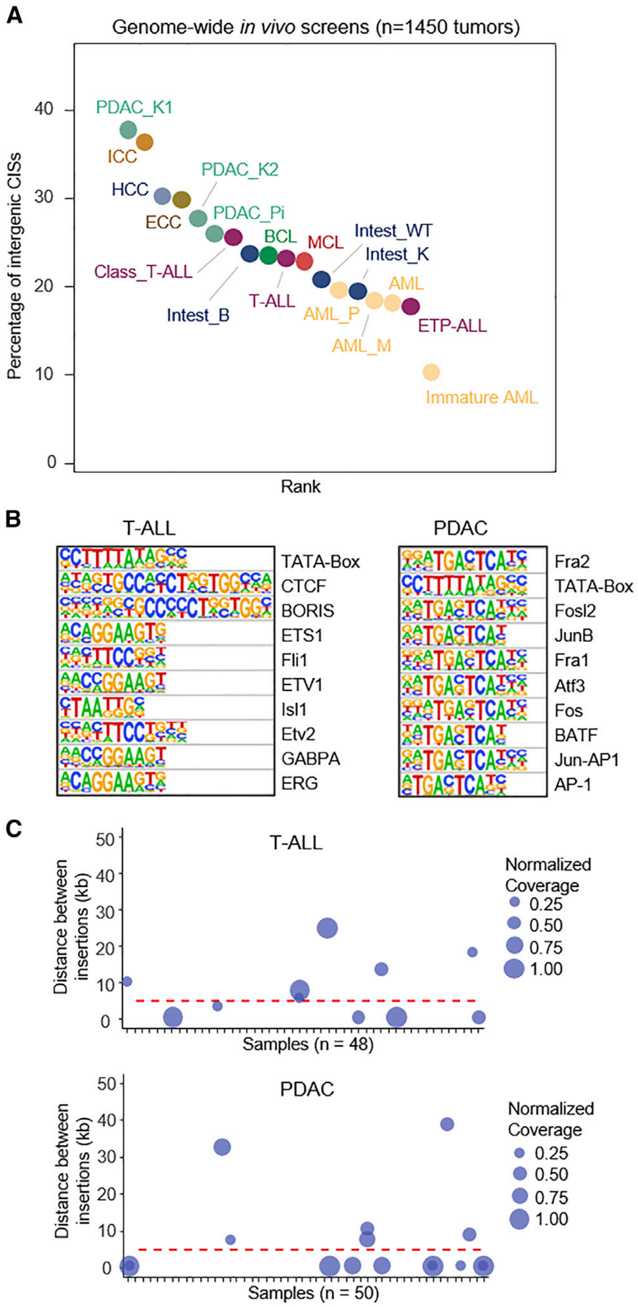


**Figure 5. Genome-wide screens reveal pervasive roles of subtle gene dysregulation across entities**

(A) Fraction of intergenic (regulatory) CISs in genome-wide *PiggyBac in vivo* screens conducted in different organs (1,450 tumors from 15 different screens). Screens were performed using either whole-body or tissue-specific activation of transposition using various Cre driver lines. CIS analyses were performed using a reduced scale parameter to identify intergenic regulatory regions (see STAR Methods). PDAC, pancreatic ductal adenocarcinoma; HCC, hepatocellular carcinoma; ECC, extrahepatic cholangiocarcinoma; ICC, intrahepatic cholangiocarcinoma; BCL, B cell lymphoma; MCL, mantle cell lymphoma; AML, acute myeloid leukemia; T-ALL, T cell acute lymphoblastic leukemia; B, screen performed in a Braf mutant context; K, Kras mutant context; WT, wild type; Pi, Pi3k mutant context.

(B) Transcription factor motif enrichment in regions of intergenic transposon insertions in two exemplary cancer types (T-ALL, PDAC). Homer analysis for known motifs was performed for a 200 bp region flanking intergenic insertions (T-ALL, n = 56,320; PDAC, 57,291). The top 10 motifs are shown (p value range T-ALL: 1e-267 to 1e-87; PDAC, 1e-322 to 1e-259).

(C) Distance of high-coverage intergenic insertions in tumor tissue (T-ALL) and primary cell cultures (PDAC). For each sample (n = 48 T-ALL; n = 50 PDAC), high-coverage ($\geq$1,000 reads) intergenic insertions (n = 328 T-ALL; n = 253 PDAC) were selected and the distance between these insertions was calculated. Distance in a range of 0–50 kb is shown, and normalized coverage for each insertion is indicated by the size of the circle. Two insertions within a distance of 50 kb in the same cancer were observed in few cases only. These likely reflect independent local hopping events on the same allele (in different cell clones) rather than biallelic insertions in the same cell.

developed no solid tumors, but almost exclusively T cell malignancies (9/10). The difference to other genotypes (11/45 T-ALL) is highly significant (p = 0.0002, Fisher's exact test) and also holds true in age-matched analyses (animals younger than 500 days: T-ALL in 8/9 vs. 10/27 mice, p = 0.0089, Fisher's exact test). At the other end of the genotype spectrum, we found that *Bcl11b*$^{\Delta 105kb/WT}$ animals did not develop T-ALL. Almost all cancers (12/13) were other than T-ALL (p = 0.013, Fisher's exact test) (Figure 4G). Thus, deletions of regulatory DNA on an otherwise wild-type background are not only sufficient to induce striking cancer phenotypes, but their nature (position, size) and dosage (hetero- or homozygosity) also profoundly affect the outcome (tumor type and frequency), suggesting additive effects and enhancer modularity. Overall, these data support a model in which even subtle gene dysregulation can significantly contribute to oncogenesis.

## Genome-wide screens reveal extensive quasi-insufficiency across entities

To explore whether subtle gene dysregulation is of broad relevance beyond T-ALL, we examined 1,450 cancers across 15 *PiggyBac* insertional mutagenesis screens, including 8 different cancer types and their subentities (Table S13). We found that 10%–38% of CISs in these screens are located in intergenic regions without an overlap to a PC gene (Figure 5A), suggesting broad relevance of subtle gene dysregulation in oncogenesis.

To examine the distribution of functional traits in non-coding CISs, we performed transcription factor motif analyses, which uncovered cancer-type-specific enrichment profiles (Figure 5B). For example, T-ALL non-coding CISs were enriched with binding motifs for ETS transcription factors (Ets1, Fli1, Erg), which have well-described roles in T cell leukemogenesis. In contrast, nPC CISs in the pancreatic screen displayed motif enrichment for transcription factors that have known functions in pancreatic acinar cell de-diferrentiation and transformation (such as Fra1/2 and Fos/Junb/Ap1.

To explore the allelic status of insertions, we manually inspected all 581 high-coverage nPC insertions in the two screens. We found only 22 cases where two insertions were within a distance of 50 kb in the same cancer (Figure 5C). These few insertions likely reflect independent hits on the same allele (in different cell clones) occurring through local transposon hopping, which is commonly observed and orders of magnitude more likely to occur than insertions on the homologous chromosome. Thus,

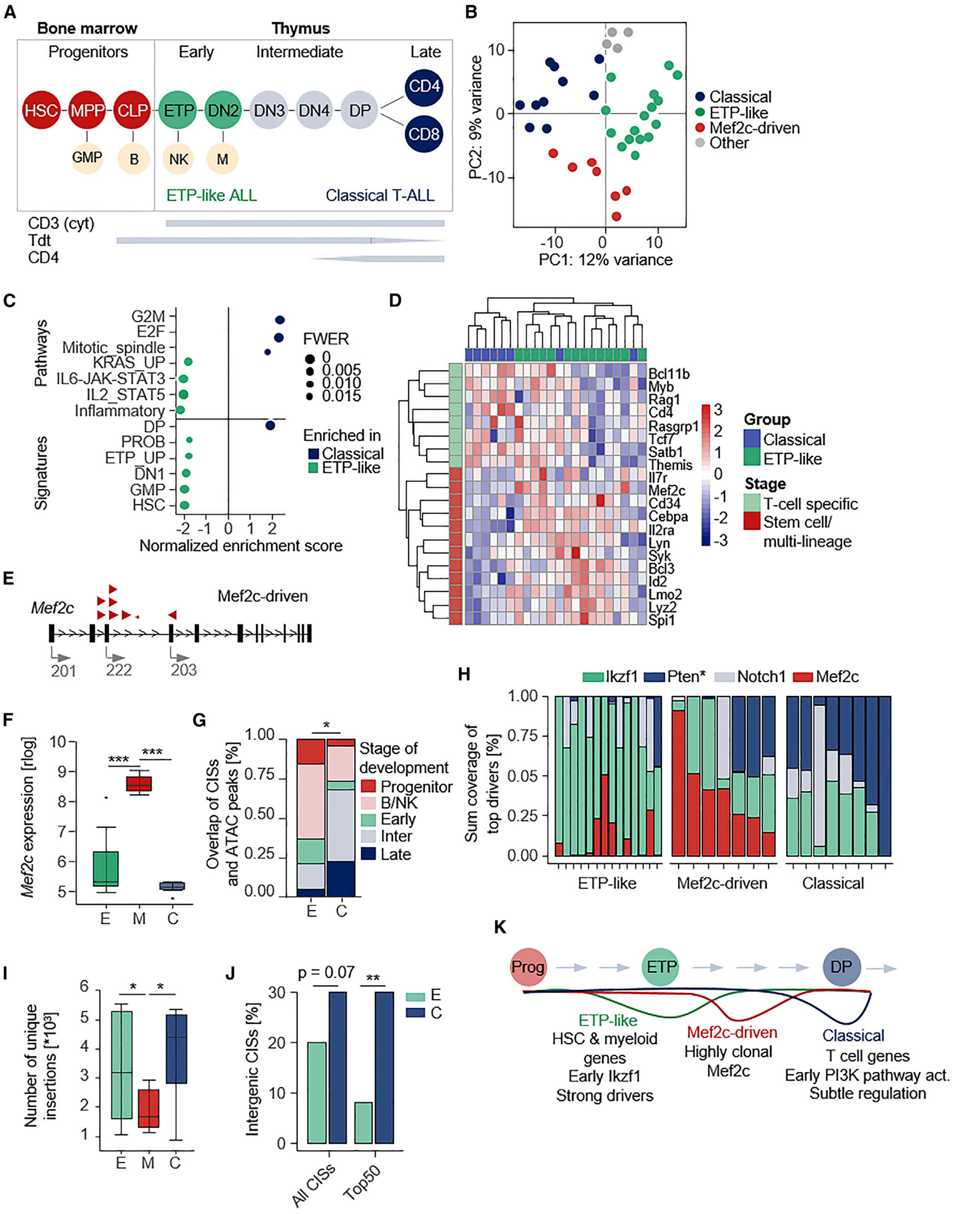


(legend on next page)

biallelic insertions affecting the same functional RE are extremely unlikely to occur, in line with the notion that non-coding insertions have subtle regulatory effects.

This conclusion is further supported by functional studies, which we performed to examine effects of intergenic transposon insertions in non-hematopoietic tumors. In a pancreas screen conducted in a *Kras*$^{G12D}$ mutant background (Figure 5), one of the top intergenic CISs marks a region with putative regulatory function located ~145 kb downstream of the nuclear receptor-interacting protein 1 (*Nrip1*) promoter. Using CRISPR-Cas9, we generated 4.5 kb deletion knockouts of this region in *Kras*$^{G12D}$ mutant mouse pancreatic cancer cells. Comparative analyses using 17 wild-type and knockout clones revealed an overall reduction of *Nrip1* expression by 30% in the knockout clones (Figure S11). Another CIS-linked regulatory region in this screen is located 80 kb downstream of *Enpp1*, a nucleotide pyrophosphatase involved in anticancer immunity. We again performed CRISPR-Cas9 knockouts of this region (3.5 kb), which resulted in downregulation of *Enpp1* (Figure S11). Thus, both experimental series support the functional relevance of intergenic transposon insertions.

We next performed mouse to human lift-over of CIS coordinates for all intergenic CISs identified in the mouse screens (n = 2,337). We observed a significant enrichment of cancer-associated GWAS variants in the syntenic human CIS regions compared with the rest of the genome ($p = 7.95 \times 10^{-7}$, $\chi^2$ test), supporting the human relevance of discoveries made in mice.

Comparison of mouse screens exposed that individual entities and subentities display striking differences in the occurrence of intergenic CISs (Figure 5A), suggesting context dependencies. In the hematopoietic system, for example, immature AMLs (defined by morphology/IHC) displayed much lower numbers of regulatory CISs than more mature forms (10% vs. 18%–19%, Figure 5A, immature AML vs. AML_P: Fisher's exact test, p = 0.045). Likewise, immature ETP-ALL have fewer regulatory CISs than their more mature "classic" T-ALL counterparts (detailed information on these analyses and T-ALL subtyping is provided below).

Altogether, these studies imply pervasive roles of quasi-insufficiency across cancers and suggest the existence of substantial context dependencies.

## T-ALL subtyping for the study of regulatory context dependencies

To explore possible context-dependent roles of quasi-insufficiency, we investigated whether our T-ALL model develops different disease subtypes. Human T-ALL is a heterogeneous disease. However, in the latest WHO classification one subset with unique biology has been recognized as a distinct sub-entity: ETP-ALL is characterized by the retention of myeloid and stem cell markers[32–34,67] (Figure 6A). To characterize mouse tumors, we analyzed transcriptomes and determined immunophenotypes of T-ALL, mature T cell lymphomas (MTL), and healthy thymus (Figure S12). IHC-based profiling revealed CD4-positive and -negative tumors, suggesting T-ALL heterogeneity in the cohort (Figure S12). Model-based clustering of gene expression data identified three major subgroups (Figures 6B and S13; Table S14). We first compared the two largest clusters using gene set enrichment analysis (Figure 6C) and found enrichment of signatures characteristic for human ETP-ALL in one group (e.g., IL6/Jak/Stat), while the second group was enriched for cell-cycle-associated genes, a characteristic of human non-ETP T-ALL (hereafter referred to as "classical" T-ALL).[67,68] Moreover, the enrichment of signatures specific for hematopoietic stem cells (HSCs), myeloid progenitors, and early T cells (ETP/DN1) in ETP-like tumors reflects their origin in early precursors, as in humans. In contrast, classical T-ALL displayed

**Figure 6. The extent of quasi-insufficiency differs in T-ALL subtypes**

(A) Schematic overview of T cell development, marker expression, and origin of T-ALL subtypes.

(B) Transcriptome-based sub-classification of T-ALLs (n = 37). Clustering was performed using model-based clustering (k = 4).

(C) Gene set enrichment analysis comparing the two major PCA clusters. Hallmark pathways and gene signatures in hematopoietic and T cell development are compared. FWER values are depicted as circles relative to significance. Enriched pathways in the ETP-like group are displayed with a negative normalized enrichment score (NES), pathways identified in the classical group are displayed with a positive NES.

(D) A murine classifier gene set (n = 20) was generated to differentiate classical and ETP-ALLs. The heatmap shows z-transformed expression values. Genes enriched in ETP were linked to early T cell development (*Mef2c*, *Il7r*, *Il2ra*, *Lmo2*), the B cell lineage (*Syk*, *Lyn*, *Bcl3*), HSCs (*Spi1*, *Cd34*, *Cebpa*, *Id2*), and the innate immune system (*Lyz2*), while classical T-ALL showed enrichment for genes associated with T cell commitment (*Tcf7*, *Bcl11b*, *Satb1*, *Cd4*), TCR rearrangement/signaling (*Rag1*, *Themis*) or specific oncogenes (*Rasgrp1*, *Myb*).

(E) Pattern of insertions in the *Mef2c* gene in samples from the Mef2c-driven subgroup. *Mef2c* possesses different isoforms, of which some are transcribed from alternative in-frame ATGs. Arrows show the orientation of insertions and indicate the direction of functionality of the transposon's unidirectional promoter. Arrow size indicates the sequencing read coverage supporting individual insertions.

(F) Expression of *Mef2c* in the three major subgroups. rlog expression value is shown (***$p < 0.001$, Wilcoxon test).

(G) Overlap of CIS regions from ETP-like and classical T-ALLs with stage-specific open chromatin peaks identified by ATAC-seq[69] in the T cell developmental lineage (*$p = 0.046$, Fisher's exact test).

(H) Sum of normalized sequencing read coverages for insertions in top CIS genes (*Ikzf1*, *Pten*, *Mef2c*, *Notch1*), indicated for each sample in the three T-ALL subtypes. The PI3K signaling and proliferation genes *Rasgrp1* and *Rpl11* (not shown) were assigned to "Pten."

(I) Number of unique insertions for each sample in indicated T-ALL subgroups: ETP-like (n = 14), Mef2c-driven (n = 7), and classical (n = 8) T-ALL (*$p < 0.05$, Student's t test).

(J) Percentage of intergenic CISs among all and the top 50 CIS regions in the classical and ETP-like subgroups (Tables S16 and S17). Intergenic CIS regions overlapping also with protein-coding genes were not considered for these analyses (**$p = 0.009$, Fisher's exact test).

(K) Simplified model of T-ALL subtype evolution. Main molecular, cellular, and temporal determinants of differential subtype evolution are shown. HSC, hematopoietic stem cell; MPP, multipotent progenitors; CLP, common lymphoid progenitor; B, B cells; ETP, early T cell precursor; NK, natural killer cells; DN, double-negative stage; DP, double-positive stage; GMP, granulocyte macrophage progenitor; M, macrophages; FWER, family-wise error rate; M, Mef2c-driven; C, classical; CIS, common insertion site.

enrichment for double-positive (DP) T cell signatures (Figures 6 and S14; Table S15). To account for the lack of mouse T-ALL classifiers, we built a 20 gene panel that separates subgroups (Figure 6D).

The characteristics of the third subgroup were initially difficult to classify based on gene-expression profiles. We therefore inspected the insertion profiles in this group and found activating insertions in *Mef2c* as a top hit in the majority of samples, as defined by high-coverage *Mef2c* insertions with sense orientation (Figure 6E). In contrast, classical or ETP-like tumors had no or predominantly low-coverage Mef2c insertions, respectively. We therefore refer to the third tumor cluster as the "Mef2c-driven" group, which indeed had high *Mef2c* expression levels (Figure 6F). Mef2c-driven T-ALLs are CD4 negative (as are ETP-like tumors), indicating their development from precursor T cells (Figure S12C). Indeed, *MEF2C* is strongly expressed in human HSCs and CLPs but not in T cells.[70] Activation of human *MEF2C* can occur through different translocations, which were associated with immature T-ALL and *MEF2C*-dependent suppression of Notch signaling.[70–72]

## Multi-scale mapping of T-ALL subtype evolution reveals context-dependent quasi-insufficiency patterns

We next investigated whether quasi-insufficiency displays context dependencies at cellular, molecular, and temporal levels in tumor evolution. To this end we integrated analyses of chromatin profiles along the T cell lineage and transposon insertion landscapes.

### *Cell of origin*

T-ALL is prototypical for tumor types that can arise from different developmental precursors or cell types. While the cell of origin can profoundly affect the biological properties of the evolving tumor, it often cannot easily be inferred through standard phenotyping. We hypothesized that, in scenarios of insertion biases (such as the preference of *PiggyBac* for open chromatin), the transposon insertion landscape in a cell population reflects a vague screenshot of global chromatin conformation at the stage of genome integration. To examine whether insertion profiles can give indications on a tumor's cell of origin, we overlapped subtype-specific CISs with regions of accessible chromatin in different cell types along the T cell developmental lineage (stage-specific ATAC-seq from Johnson and co-workers[69]). We found that 79% of CISs in ETP-like tumors overlap with ATAC peaks specific for progenitor, natural killer, B, or early T cells, while classical T-ALL CISs overlap predominantly (68%) with regions of accessible chromatin that are specific for intermediate and late stages of T cell development (Figure 6G). These data suggest that, despite ongoing transposon mobilization, some insertions carry a certain level of "historical information" that allows us to infer developmental origin, a concept that could possibly be expanded to the study of other cancer types.

### *Sequentiality*

The molecular determinants driving individual stages of tumor evolution in different T-ALL subtypes are poorly understood.[73,74] Interrogation of such evolutionary principles in our dataset requires clonal deconvolution, which is, however, not supported by standard CIS calling algorithms. This is due to the non-quantitative statistical concept used to search for genomic "insertion hotspots" in a cohort of mice. To overcome this problem, we conducted a second type of analysis, which integrates quantitative data for each of the 170,000 non-redundant insertions based on quantitative insertion site sequencing, a method we developed earlier for this purpose.[40] For each cancer, read coverages supporting individual insertions (range: 2 to 10,000) reflect their clonal distribution and likely position at the tumor's evolutionary tree.

These analyses revealed that, while top CISs are shared between T-ALL subtypes, their clonal distribution differs markedly, indicating distinct evolutionary hierarchies (Figure 6H). The most prominent hit in ETP-like tumors was *Ikzf1*, which is supported by very high read coverages in virtually all tumors (Figure 6H). Strong positive selection for *Ikzf1* insertions in virtually all ETP-ALL establishes a critical role of *Ikzf1* in the initiation of this T-ALL subtype. In contrast, *Pten*, which was characterized by highly subclonal insertions in ETP-like tumors, was the dominant high-coverage hit in classical T-ALL (Figure 6H), indicating differential temporal orders of tumor-driving events in T-ALL subtypes. Importantly, chromatin accessibility at *Ikzf1* and *Pten* (or other main drivers, including *Notch1* and *Mef2c*) is similar at different stages of T cell development (that is in the different cell types from which T-ALL subtypes arise; Figure S15), excluding the possibility of integration biases driving the differential distribution or sequentiality of driver gene insertions in T-ALL subtypes.

### *Intratumor heterogeneity*

We next exploited screening data to infer global characteristics of clonal architecture in different T-ALL subtypes. The analyses revealed that the Mef2c-driven group differs from other subtypes in that it displays: (1) fewer CISs, which is also true in sample-size-matched analyses (Figures S16A and S16B) and (2) reduced numbers of total insertions per tumor (Figures 6I and S16C). In humans, there is controversy as to whether MEF2C dysregulated and ETP-ALL feature a single or distinct disease entities.[75,76] Our results support the latter by highlighting substantial biological differences between subtypes.

### *Subtype-specific driver genes*

We next performed subgroup-specific CIS analyses (Figures S16D–S16F; Tables S16–S18). CISs specific for ETP-ALL affected mature T cell genes (inactivation of *Ikzf2*, *Ikzf3*), Ras pathway components (*Rapgef2*, *Nf1*), and potential negative regulators of Wnt signaling (*Kremen1*, *Tmem170b*; not linked to T-ALL so far). Moreover, several genes linked to stemness or the myeloid lineage were among the top hits in this group (*Cnr2*, *Chd2*, *Crebbp*, *Mecom*) (Figures S16D and S16E), which sheds light on several open questions in human ETP-ALL biology (detailed in the discussion). In the classical T-ALL subgroup, two observations stood out in addition to the predominance of *Pten* hits described above: (1) recurrent insertions in genes linked to late thymocyte development (*Tcf12*, *Rpl5*), consistent with the notion of classical T-ALLs arising from post-commitment DP cells, and (2) a large number of CISs affecting intergenic REs, especially among the top CISs (Figures 6J, S16D, and S16F), suggesting a so far unappreciated importance of subtle gene regulation, specifically in classical T-ALL.

Collectively, these results uncovered key characteristics of different T-ALL subtypes (Figures 6K and S16G) and highlight the ability of our experimental system to interrogate tumor evolutionary principles in space and time. Their application exposed

how the complex interplay of distinct cellular contexts, molecular triggers, and the temporal dynamics of their alterations and regulatory interactions drive cancer evolution along different trajectories that give rise to distinct cancer phenotypes/subentities.

## DISCUSSION

During oncogenesis, regulatory landscapes undergo extensive changes, captured by global profiling studies, but not well understood at the functional level. Determining whether an alteration is cancer causing (driver) or neutral (passenger) is a challenge in epigenetics, more so than in structural cancer genomics and mutational profiling. Systematic *in vivo* perturbation of the non-coding genomic space is—given its enormous size and limited annotation—difficult to achieve by targeted approaches, such as library-based CRISPR screens (which face size limits). Even greater restraints to screening scalability arise from the hurdles associated with somatic delivery of libraries to many organs and cell types.[20,77] Insertional mutagenesis using endogenous transposon systems addresses these limitations. Continuous mobilization and random reintegration of multiple transposons in every single cell of the mouse produces enormous mutational complexity, constituting a pool of hundreds of billions of alterations in non-transformed cells that are then subject to selection. We developed universally applicable methods for interrogation of the nPC genome using transposon mutagenesis. Applied to the evolution in T-ALL, the screens assembled catalogs of cancer-relevant REs and nPC transcripts, constituting the first systematic survey of its kind.

Another area of cancer research that faces methodological constraints is evolutionary genetics. In human cancer samples, genetic evolution is inferred retrospectively. Owing to selection and clonal sweep, such analyses typically capture sequentiality of the latest mutations only. Even more challenging is the discovery of subtle, often temporally restricted (and reversible) regulatory processes during tumor evolution, or the capture of other determinants, such as the cell of origin. We devised methods for prospective interrogation of cancer evolution by intersecting epigenomic data along the T cell developmental lineage and insertional mutagenesis to induce different T-ALL subentities. We mapped perturbations driving evolution, including their type and quality, regulatory fine-tuning, combinatorial code, temporal sequence, and cellular/evolutionary history (Figure S16G). Capturing the complex interactions of these different layers provided multifaceted new insights into T-ALL subtype evolution (Figure 6K) and explain several open questions in the field.

It was reported earlier that *IKZF1* alterations are enriched in human ETP-ALL compared with classic T-ALL.[67] The screens in mice add both functional and temporal information to this association by linking *Ikzf1* to the initiation of this T-ALL subtype. Moreover, ETP-ALL displayed exclusivity for a number of screening hits, including genes or REs/gene pairs linked to stemness or the myeloid lineage. Coupled with the observation that stem cell and/or myeloid markers are expressed in human ETP-ALL,[78] the latter suggests a specific vulnerability of pre-commitment progenitors (but not committed T cells) to transformation by insults sustaining the lympho-myeloid program. The variety of myeloid/stem cell-related CISs we found in ETP-like ALL indicates that various such triggers can contribute to ETP cell transformation. This might explain the phenotypic diversity of human ETP-ALL, which express different combinations of stem cell and/or myeloid markers.[33] We speculate that distinct genetic interactions define whether perturbation of such "myeloid/stem cell" genes in precursors promotes AML or rather ETP-ALL development. Indeed, while in our AML screen *Mecom* activation was found as a top truncal driver (unpublished data), *Mecom* insertions in ETP-ALL were preceded by truncal *Notch1* and *Ikzf1* insertions.

Our screening system constitutes a model for Mef2c-driven T-ALL, which gave new insights into the biology of this sub-entity. In humans, there is controversy as to whether MEF2C dysregulated and ETP-ALL feature a single disease entity or should be regarded as distinct groups (there is often partial discordance of immunophenotypic characteristics[75,76]). The mouse model supports the latter. It shows that there are biological differences between ETP-like and Mef2c-driven tumors at various levels, including their transcriptional profiles, driver genes or clonal architectures. This might also explain differences in treatment response and resistance between groups as human MEF2C T-ALL respond poorly to glucocorticoids.[76]

The dominance of high-coverage *Pten* hits in classical mouse T-ALLs (vs. low-coverage hits in ETP-ALL) highlights differential sequentiality of tumor driving events in T-ALL subtypes. Strong selection for *Pten* inactivation in our functional screens likely reflects subtype-specific constraints or exigencies during early evolution of classical T-ALL and rationalizes the enrichment of PI3K pathway alterations in the equivalent mature forms of human T-ALL.[49,79] Another observation that stood out in the classical subgroup was the large number of CISs affecting intergenic REs, suggesting a so far unappreciated importance of subtle gene regulation specifically in classical T-ALL. The evolutionary pressures underlying these observations remain to be explored. It is possible, for example, that classical T-ALL rely on establishing a fine-tuned dosage reduction of T cell commitment genes rather than their complete inactivation, which might either be deleterious at this stage or lead to a phenotypic switch toward a less differentiated state.

Over the past two decades, much effort in cancer genetics has focused on identifying coding mutations, a process that had transformative impact in cancer biology. Our results suggest, however, that—beyond coding mutations—a vast and so far understudied layer of molecular dysregulations contributes to oncogenesis. The screens across many cancer types described here provide evidence for widespread haplo- and quasi-insufficiency in tumor evolution, and we show in mouse models that even small or temporal gene expression changes of tumor suppressors, such as *Bcl11b*, can be oncogenic. Transposon insertions affecting REs predominantly cause subtle gene dysregulations, as do human cancer risk variants, which are commonly located in the nPC genome. The strong enrichment of GWAS hotspots in our catalogs of regulatory CISs thus reinforces the human relevance of this study.

Functional experiments targeting cancer-relevant REs provided mechanistic insights into the pathogenic outcomes of gradual gene dysregulations. Allelic deletion series that we engineered in a large "gene desert" downstream *Bcl11b* in mice

displayed striking developmental and cancer phenotypes. This not only confirmed the predictions made by the screen, but also provided new insights into the biology of the most frequent human translocation in pediatric T-ALL. Allele-specific differences in tumor penetrance, latency, and spectra/types reflect gradual gene dysregulations through enhancer modularity, with additive effects and tissue-specific phenotypic outcomes—concepts that require organismal models, such as the ones developed here, for their interrogation and proof.

Global interrogation of quasi-insufficiency requires methodology capable to induce genome-wide subtle perturbations—in experimental systems that can capture the relevant readout, that is cancer development in an organism. Our screening approach fulfills these requirements and enabled a comprehensive survey toward a systems-level understanding of subtle gene dysregulation in cancer. Both, our studies covering multiple-entities as well as the focused hematopoietic screens revealed (sub)entity-specific differences in the global extent of RE alterations and quasi-insufficiency. Subtle gene dysregulations were less predominant, for example, in immature T-ALL than in tumors originating from committed T cells, an observation that was also mirrored in screens for myeloid malignancies. Selection of fine-tuned rewiring of signaling networks during transformation is not surprising, nor is its context-specific variance: depending on the cell of origin and oncogenic insult, the path between oncogenic cell fate changes and cell death can be narrow, requiring precise orchestration of molecular reprogramming during transformation.

### Limitations of the study

One limitation of the screening approach is the dependence of transposon-induced gene activation on splicing. As a result, all genes having their translation initiation codon in exon-1 (first exons do not have a splice acceptor) cannot be activated by the promoter engineered into transposons, unless the genes possess an alternative in-frame ATG. Another limitation is the difficulty to predict effects of insertions in the non-coding genomic space. This contrasts the analysis of the protein-coding CISs, where transposon insertion patterns predict whether gene activation or inactivation is the cancer-driving mechanism. In principle, transposon insertions in regulatory regions can positively or negatively affect expression of target genes. Possible mechanisms underlying repressive effects include disruption of transcription factor binding sites, interference with 3D chromosomal conformation and topology-associated domain structure. In contrast, gene activation can in principle be mediated by the transposon's activating elements or the disruption of silencer/insulator sequences. In this study, effects related to selected intergenic CISs were validated functionally. Finally, some of the analyses comparing different T-ALL subentities would have benefited from a larger sample size, which may increase the power of the discovery approach.

## STAR★METHODS

Detailed methods are provided in the online version of this paper and include the following:


- KEY RESOURCES TABLE
- RESOURCE AVAILABILITY
  - Lead contact
  - Materials availability
  - Data and code availability
- EXPERIMENTAL MODEL AND SUBJECT DETAILS
  - Mouse strains
  - Cell lines
  - Human subjects
- METHOD DETAILS
  - Generation of mouse strains and cohorts
  - Necropsy and histopathological analysis
  - Immunohistochemistry
  - Quantitative transposon insertion site sequencing
  - CIS calling and downstream analyses
  - Footprint plots and transcription factor motif search
  - CIS annotation pipeline (ARCIS)
  - GRO-Seq
  - Cell-culture-based CRISPR-Cas9 knockout experiments
  - DNA and RNA isolation
  - cDNA synthesis and qPCR
  - 3-Prime RNA sequencing
  - RNA-seq data analysis
  - GSEA
  - Analyses of GWAS data
- QUANTIFICATION AND STATISTICAL ANALYSIS
  - General statistical analyses
  - CIS analysis
  - RNA-seq data analysis
  - GSEA
  - Analyses of GWAS data


## SUPPLEMENTAL INFORMATION

Supplemental information can be found online at https://doi.org/10.1016/j.xgen.2023.100276.

## ACKNOWLEDGMENTS

We thank Julia Eichinger, Anja Grotloh, Markus Utzt, Danijela Heide, Marion Mielke, and Olga Seelbach for excellent technical assistance. We further thank the Emil Aaltonen Foundation for funding the human sample generation and the EMBL GeneCore (https://www.genecore.embl.de/index.cfm) for sequencing services. We thank Carl Bredthauer, Ata Ahari, Leonhard Wachutka, and Julien Gagneur for performing Transmicron analysis.

This study was supported by the German Cancer Consortium (DKTK), the Deutsche Forschungsgemeinschaft (DFG RA 1629/2-1 to R.R.; DFG SA 1374/4-2 to D.S.; SFB 1321 to R.R., J.M., D.S., M.S.S., and G.S.; and SFB 1371 to D.S.), the Deutsche Krebshilfe (70114314 to R.R.), the German Federal Ministry of Education and Research (CNATM Cluster, to R.R.), and the European Research Council (CoG PACA-MET no. 819642 and MSCA-ITN-ETN PRECODE to R.R., CoG no. 648521 to D.S.). J.C. is supported by Fundación María Cristina Masaveu Peterson.

## AUTHOR CONTRIBUTIONS

A.F., N.C., A.B., L.R., and R.R. designed different parts of the study. A.F., R.L., A.S., M.J.F., J.W., R.Ö., D.S., L.K., T.K., C.K., M.R., T.A., J.J.M., N.C., and L.R. performed the research. A.F., N.d.A.K., M.J.F., and T.E. conducted bioinformatic analyses. H.-Y.Y., U.K., I.G.-M., L.Q.-M., and K.S. performed histopathological assessment. M.L., S.L., O.L., and M. Heinäniemi generated and analyzed human GRO-seq data. G.S., J.M., M. Heikenwälder, M.S.-S., P.L., J.C., G.S.V., D.S., O.L., and M. Heinäniemi provided resources and critical

input. N.C., A.B., L.R., and R.R. supervised the study. A.F. and R.R. wrote the manuscript.

## DECLARATION OF INTERESTS

The authors declare no competing interests.

## INCLUSION AND DIVERSITY

We support inclusive, diverse, and equitable conduct of research.

Received: March 10, 2022
Revised: September 5, 2022
Accepted: February 8, 2023
Published: March 8, 2023

## REFERENCES

1. Stratton, M.R., Campbell, P.J., and Futreal, P.A. (2009). The cancer genome. Nature *458*, 719–724.
2. Bradner, J.E., Hnisz, D., and Young, R.A. (2017). Transcriptional addiction in cancer. Cell *168*, 629–643.
3. Knudson, A.G., Jr. (1971). Mutation and cancer: statistical study of retinoblastoma. Proc. Natl. Acad. Sci. USA *68*, 820–823.
4. Friend, S.H., Bernards, R., Rogelj, S., Weinberg, R.A., Rapaport, J.M., Albert, D.M., and Dryja, T.P. (1986). A human DNA segment with properties of the gene that predisposes to retinoblastoma and osteosarcoma. Nature *323*, 643–646.
5. Donehower, L.A., Harvey, M., Slagle, B.L., McArthur, M.J., Montgomery, C.A., Jr., Butel, J.S., and Bradley, A. (1992). Mice deficient for p53 are developmentally normal but susceptible to spontaneous tumours. Nature *356*, 215–221.
6. Lee, E.Y., Chang, C.Y., Hu, N., Wang, Y.C., Lai, C.C., Herrup, K., Lee, W.H., and Bradley, A. (1992). Mice deficient for Rb are nonviable and show defects in neurogenesis and haematopoiesis. Nature *359*, 288–294.
7. Venkatachalam, S., Shi, Y.P., Jones, S.N., Vogel, H., Bradley, A., Pinkel, D., and Donehower, L.A. (1998). Retention of wild-type p53 in tumors from p53 heterozygous mice: reduction of p53 dosage can promote cancer formation. Embo j *17*, 4657–4667.
8. Di Cristofano, A., Kotsi, P., Peng, Y.F., Cordon-Cardo, C., Elkon, K.B., and Pandolfi, P.P. (1999). Impaired Fas response and autoimmunity in Pten+/- mice. Science *285*, 2122–2125.
9. Berger, A.H., and Pandolfi, P.P. (2011). Haplo-insufficiency: a driving force in cancer. J. Pathol. *223*, 137–146.
10. Mueller, S., Engleitner, T., Maresch, R., Zukowska, M., Lange, S., Kaltenbacher, T., Konukiewitz, B., Öllinger, R., Zwiebel, M., Strong, A., et al. (2018). Evolutionary routes and KRAS dosage define pancreatic cancer phenotypes. Nature *554*, 62–68.
11. Wartewig, T., Kurgyis, Z., Keppler, S., Pechloff, K., Hameister, E., Öllinger, R., Maresch, R., Buch, T., Steiger, K., Winter, C., et al. (2017). PD-1 is a haploinsufficient suppressor of T cell lymphomagenesis. Nature *552*, 121–125.
12. de la Rosa, J., Weber, J., Friedrich, M.J., Li, Y., Rad, L., Ponstingl, H., Liang, Q., de Quirós, S.B., Noorani, I., Metzakopian, E., et al. (2017). A single-copy Sleeping Beauty transposon mutagenesis screen identifies new PTEN-cooperating tumor suppressor genes. Nat. Genet. *49*, 730–741.
13. Alimonti, A., Carracedo, A., Clohessy, J.G., Trotman, L.C., Nardella, C., Egia, A., Salmena, L., Sampieri, K., Haveman, W.J., Brogi, E., et al. (2010). Subtle variations in Pten dose determine cancer susceptibility. Nat. Genet. *42*, 454–458.
14. Berger, A.H., Knudson, A.G., and Pandolfi, P.P. (2011). A continuum model for tumour suppression. Nature *476*, 163–169.
15. Sarkisian, C.J., Keister, B.A., Stairs, D.B., Boxer, R.B., Moody, S.E., and Chodosh, L.A. (2007). Dose-dependent oncogene-induced senescence in vivo and its evasion during mammary tumorigenesis. Nat. Cell Biol. *9*, 493–505.
16. Murphy, D.J., Junttila, M.R., Pouyet, L., Karnezis, A., Shchors, K., Bui, D.A., Brown-Swigart, L., Johnson, L., and Evan, G.I. (2008). Distinct thresholds govern Myc's biological output in vivo. Cancer Cell *14*, 447–457.
17. Junttila, M.R., Karnezis, A.N., Garcia, D., Madriles, F., Kortlever, R.M., Rostker, F., Brown Swigart, L., Pham, D.M., Seo, Y., Evan, G.I., and Martins, C.P. (2010). Selective activation of p53-mediated tumour suppression in high-grade tumours. Nature *468*, 567–571.
18. Feldser, D.M., Kostova, K.K., Winslow, M.M., Taylor, S.E., Cashman, C., Whittaker, C.A., Sanchez-Rivera, F.J., Resnick, R., Bronson, R., Hemann, M.T., and Jacks, T. (2010). Stage-specific sensitivity to p53 restoration during lung cancer progression. Nature *468*, 572–575.
19. Rad, R., Cadiñanos, J., Rad, L., Varela, I., Strong, A., Kriegl, L., Constantino-Casas, F., Eser, S., Hieber, M., Seidler, B., et al. (2013). A genetic progression model of Braf(V600E)-induced intestinal tumorigenesis reveals targets for therapeutic intervention. Cancer Cell *24*, 15–29.
20. Weber, J., Braun, C.J., Saur, D., and Rad, R. (2020). In vivo functional screening for systems-level integrative cancer genomics. Nat. Rev. Cancer *20*, 573–593.
21. Elliott, K., and Larsson, E. (2021). Non-coding driver mutations in human cancer. Nat. Rev. Cancer *21*, 500–509.
22. Khurana, E., Fu, Y., Chakravarty, D., Demichelis, F., Rubin, M.A., and Gerstein, M. (2016). Role of non-coding sequence variants in cancer. Nat. Rev. Genet. *17*, 93–108.
23. Rheinbay, E., Nielsen, M.M., Abascal, F., Wala, J.A., Shapira, O., Tiao, G., Hornshøj, H., Hess, J.M., Juul, R.I., Lin, Z., et al. (2020). Analyses of non-coding somatic drivers in 2,658 cancer whole genomes. Nature *578*, 102–111.
24. Lee, J.T. (2012). Epigenetic regulation by long noncoding RNAs. Science *338*, 1435–1439.
25. Lawrence, M.S., Stojanov, P., Polak, P., Kryukov, G.V., Cibulskis, K., Sivachenko, A., Carter, S.L., Stewart, C., Mermel, C.H., Roberts, S.A., et al. (2013). Mutational heterogeneity in cancer and the search for new cancer-associated genes. Nature *499*, 214–218.
26. Hu, S., Qian, M., Zhang, H., Guo, Y., Yang, J., Zhao, X., He, H., Lu, J., Pan, J., Chang, M., et al. (2017). Whole-genome noncoding sequence analysis in T-cell acute lymphoblastic leukemia identifies oncogene enhancer mutations. Blood *129*, 3264–3268.
27. Hnisz, D., Weintraub, A.S., Day, D.S., Valton, A.L., Bak, R.O., Li, C.H., Goldmann, J., Lajoie, B.R., Fan, Z.P., Sigova, A.A., et al. (2016). Activation of proto-oncogenes by disruption of chromosome neighborhoods. Science *351*, 1454–1458.
28. Mansour, M.R., Abraham, B.J., Anders, L., Berezovskaya, A., Gutierrez, A., Durbin, A.D., Etchin, J., Lawton, L., Sallan, S.E., Silverman, L.B., et al. (2014). Oncogene regulation. An oncogenic super-enhancer formed through somatic mutation of a noncoding intergenic element. Science *346*, 1373–1377.
29. Abraham, B.J., Hnisz, D., Weintraub, A.S., Kwiatkowski, N., Li, C.H., Li, Z., Weichert-Leahey, N., Rahman, S., Liu, Y., Etchin, J., et al. (2017). Small genomic insertions form enhancers that misregulate oncogenes. Nat. Commun. *8*, 14385.
30. Herranz, D., Ambesi-Impiombato, A., Palomero, T., Schnell, S.A., Belver, L., Wendorff, A.A., Xu, L., Castillo-Martin, M., Llobet-Navás, D., Cordon-Cardo, C., et al. (2014). A NOTCH1-driven MYC enhancer promotes T cell development, transformation and acute lymphoblastic leukemia. Nat. Med. *20*, 1130–1137.
31. Li, Z., Abraham, B.J., Berezovskaya, A., Farah, N., Liu, Y., Leon, T., Fielding, A., Tan, S.H., Sanda, T., Weintraub, A.S., et al. (2017). APOBEC

signature mutation generates an oncogenic enhancer that drives LMO1 expression in T-ALL. Leukemia *31*, 2057–2064.

32. Arber, D.A., Orazi, A., Hasserjian, R., Thiele, J., Borowitz, M.J., Le Beau, M.M., Bloomfield, C.D., Cazzola, M., and Vardiman, J.W. (2016). The 2016 revision to the World Health Organization classification of myeloid neoplasms and acute leukemia. Blood *127*, 2391–2405.
33. Coustan-Smith, E., Mullighan, C.G., Onciu, M., Behm, F.G., Raimondi, S.C., Pei, D., Cheng, C., Su, X., Rubnitz, J.E., Basso, G., et al. (2009). Early T-cell precursor leukaemia: a subtype of very high-risk acute lymphoblastic leukaemia. Lancet Oncol. *10*, 147–156.
34. Jain, N., Lamb, A.V., O'Brien, S., Ravandi, F., Konopleva, M., Jabbour, E., Zuo, Z., Jorgensen, J., Lin, P., Pierce, S., et al. (2016). Early T-cell precursor acute lymphoblastic leukemia/lymphoma (ETP-ALL/LBL) in adolescents and adults: a high-risk subtype. Blood *127*, 1863–1869.
35. Dupuy, A.J., Akagi, K., Largaespada, D.A., Copeland, N.G., and Jenkins, N.A. (2005). Mammalian mutagenesis using a highly mobile somatic Sleeping Beauty transposon system. Nature *436*, 221–226.
36. Collier, L.S., Carlson, C.M., Ravimohan, S., Dupuy, A.J., and Largaespada, D.A. (2005). Cancer gene discovery in solid tumours using transposon-based somatic mutagenesis in the mouse. Nature *436*, 272–276.
37. Rad, R., Rad, L., Wang, W., Cadinanos, J., Vassiliou, G., Rice, S., Campos, L.S., Yusa, K., Banerjee, R., Li, M.A., et al. (2010). PiggyBac transposon mutagenesis: a tool for cancer gene discovery in mice. Science *330*, 1104–1107.
38. Rad, R., Rad, L., Wang, W., Strong, A., Ponstingl, H., Bronner, I.F., Mayho, M., Steiger, K., Weber, J., Hieber, M., et al. (2015). A conditional piggyBac transposition system for genetic screening in mice identifies oncogenic networks in pancreatic cancer. Nat. Genet. *47*, 47–56.
39. Weber, J., de la Rosa, J., Grove, C.S., Schick, M., Rad, L., Baranov, O., Strong, A., Pfaus, A., Friedrich, M.J., Engleitner, T., et al. (2019). PiggyBac transposon tools for recessive screening identify B-cell lymphoma drivers in mice. Nat. Commun. *10*, 1415.
40. Friedrich, M.J., Rad, L., Bronner, I.F., Strong, A., Wang, W., Weber, J., Mayho, M., Ponstingl, H., Engleitner, T., Grove, C., et al. (2017). Genome-wide transposon screening and quantitative insertion site sequencing for cancer gene discovery in mice. Nat. Protoc. *12*, 289–309.
41. de Ridder, J., Uren, A., Kool, J., Reinders, M., and Wessels, L. (2006). Detecting statistically significant common insertion sites in retroviral insertional mutagenesis screens. PLoS Comput. Biol. *2*, e166.
42. Liu, Y., Easton, J., Shao, Y., Maciaszek, J., Wang, Z., Wilkinson, M.R., McCastlain, K., Edmonson, M., Pounds, S.B., Shi, L., et al. (2017). The genomic landscape of pediatric and young adult T-lineage acute lymphoblastic leukemia. Nat. Genet. *49*, 1211–1218.
43. Hu, G., Cui, K., Fang, D., Hirose, S., Wang, X., Wangsa, D., Jin, W., Ried, T., Liu, P., Zhu, J., et al. (2018). Transformation of accessible chromatin and 3D nucleome underlies lineage commitment of early T cells. Immunity *48*, 227–242.e8.
44. Ren, G., Jin, W., Cui, K., Rodrigez, J., Hu, G., Zhang, Z., Larson, D.R., and Zhao, K. (2017). CTCF-mediated enhancer-promoter interaction is a critical regulator of cell-to-cell variation of gene expression. Mol. Cell *67*, 1049–1058.e6.
45. de Jong, J., Akhtar, W., Badhai, J., Rust, A.G., Rad, R., Hilkens, J., Berns, A., van Lohuizen, M., Wessels, L.F.A., and de Ridder, J. (2014). Chromatin landscapes of retroviral and transposon integration profiles. PLoS Genet. *10*, e1004250.
46. Yoshida, J., Akagi, K., Misawa, R., Kokubu, C., Takeda, J., and Horie, K. (2017). Chromatin states shape insertion profiles of the piggyBac, Tol2 and Sleeping Beauty transposons and murine leukemia virus. Sci. Rep. *7*, 43613.
47. Gogol-Döring, A., Ammar, I., Gupta, S., Bunse, M., Miskey, C., Chen, W., Uckert, W., Schulz, T.F., Izsvák, Z., and Ivics, Z. (2016). Genome-wide profiling reveals remarkable parallels between insertion site selection properties of the MLV retrovirus and the piggyBac transposon in primary human CD4(+) T cells. Mol. Ther. *24*, 592–606.
48. Li, M.A., Pettitt, S.J., Eckert, S., Ning, Z., Rice, S., Cadiñanos, J., Yusa, K., Conte, N., and Bradley, A. (2013). The piggyBac transposon displays local and distant reintegration preferences and can cause mutations at noncanonical integration sites. Mol. Cell Biol. *33*, 1317–1330.
49. Zuurbier, L., Petricoin, E.F., 3rd, Vuerhard, M.J., Calvert, V., Kooi, C., Buijs-Gladdines, J.G.C.A.M., Smits, W.K., Sonneveld, E., Veerman, A.J.P., Kamps, W.A., et al. (2012). The significance of PTEN and AKT aberrations in pediatric T-cell acute lymphoblastic leukemia. Haematologica *97*, 1405–1413.
50. Palomero, T., Sulis, M.L., Cortina, M., Real, P.J., Barnes, K., Ciofani, M., Caparros, E., Buteau, J., Brown, K., Perkins, S.L., et al. (2007). Mutational loss of PTEN induces resistance to NOTCH1 inhibition in T-cell leukemia. Nat. Med. *13*, 1203–1210.
51. Choi, S., Warzecha, C., Zvezdova, E., Lee, J., Argenty, J., Lesourne, R., Aravind, L., and Love, P.E. (2017). THEMIS enhances TCR signaling and enables positive selection by selective inhibition of the phosphatase SHP-1. Nat. Immunol. *18*, 433–441.
52. Liu, C., Somasundaram, A., Manne, S., Gocher, A.M., Szymczak-Workman, A.L., Vignali, K.M., Scott, E.N., Normolle, D.P., John Wherry, E., Lipson, E.J., et al. (2020). Neuropilin-1 is a T cell memory checkpoint limiting long-term antitumor immunity. Nat. Immunol. *21*, 1010–1021.
53. Tottone, L., Lancho, O., Loh, J.W., Singh, A., Kimura, S., Roels, J., Kuchmiy, A., Strubbe, S., Lawlor, M.A., da Silva-Diz, V., et al. (2021). A tumor suppressor enhancer of PTEN in T-cell development and leukemia. Blood Cancer Discov. *2*, 92–109.
54. Lahortiga, I., De Keersmaecker, K., Van Vlierberghe, P., Graux, C., Cauwelier, B., Lambert, F., Mentens, N., Beverloo, H.B., Pieters, R., Speleman, F., et al. (2007). Duplication of the MYB oncogene in T cell acute lymphoblastic leukemia. Nat. Genet. *39*, 593–595.
55. Palomero, T., Lim, W.K., Odom, D.T., Sulis, M.L., Real, P.J., Margolin, A., Barnes, K.C., O'Neil, J., Neuberg, D., Weng, A.P., et al. (2006). NOTCH1 directly regulates c-MYC and activates a feed-forward-loop transcriptional network promoting leukemic cell growth. Proc. Natl. Acad. Sci. USA *103*, 18261–18266.
56. Porcu, M., Kleppe, M., Gianfelici, V., Geerdens, E., De Keersmaecker, K., Tartaglia, M., Foà, R., Soulier, J., Cauwelier, B., Uyttebroeck, A., et al. (2012). Mutation of the receptor tyrosine phosphatase PTPRC (CD45) in T-cell acute lymphoblastic leukemia. Blood *119*, 4476–4479.
57. Su, W., Xu, M., Chen, X., Chen, N., Gong, J., Nie, L., Li, L., Li, X., Zhang, M., and Zhou, Q. (2017). Long noncoding RNA ZEB1-AS1 epigenetically regulates the expressions of ZEB1 and downstream molecules in prostate cancer. Mol. Cancer *16*, 142.
58. Higashi, Y., Moribe, H., Takagi, T., Sekido, R., Kawakami, K., Kikutani, H., and Kondoh, H. (1997). Impairment of T cell development in deltaEF1 mutant mice. J. Exp. Med. *185*, 1467–1479.
59. Soen, B., Vandamme, N., Berx, G., Schwaller, J., Van Vlierberghe, P., and Goossens, S. (2018). ZEB proteins in leukemia: friends, foes, or friendly foes? Hemasphere *2*, e43.
60. Machiela, M.J., and Chanock, S.J. (2015). LDlink: a web-based application for exploring population-specific haplotype structure and linking correlated alleles of possible functional variants. Bioinformatics *31*, 3555–3557.
61. Belver, L., and Ferrando, A. (2016). The genetics and mechanisms of T cell acute lymphoblastic leukaemia. Nat. Rev. Cancer *16*, 494–507.
62. Su, X.Y., Della-Valle, V., Andre-Schmutz, I., Lemercier, C., Radford-Weiss, I., Ballerini, P., Lessard, M., Lafage-Pochitaloff, M., Mugneret, F., Berger, R., et al. (2006). HOX11L2/TLX3 is transcriptionally activated through T-cell regulatory elements downstream of BCL11B as a result of the t(5;14)(q35;q32). Blood *108*, 4198–4201.
63. Nagel, S., Scherr, M., Kel, A., Hornischer, K., Crawford, G.E., Kaufmann, M., Meyer, C., Drexler, H.G., and MacLeod, R.A.F. (2007). Activation of

TLX3 and NKX2-5 in t(5;14)(q35;q32) T-cell acute lymphoblastic leukemia by remote 3'-BCL11B enhancers and coregulation by PU.1 and HMGA1. Cancer Res. *67*, 1461–1471.

64. Li, L., Zhang, J.A., Dose, M., Kueh, H.Y., Mosadeghi, R., Gounari, F., and Rothenberg, E.V. (2013). A far downstream enhancer for murine Bcl11b controls its T-cell specific expression. Blood *122*, 902–911.
65. Isoda, T., Moore, A.J., He, Z., Chandra, V., Aida, M., Denholtz, M., Piet van Hamburg, J., Fisch, K.M., Chang, A.N., Fahl, S.P., et al. (2017). Non-coding transcription instructs chromatin folding and compartmentalization to dictate enhancer-promoter communication and T cell fate. Cell *171*, 103–119.e18.
66. Lennon, M.J., Jones, S.P., Lovelace, M.D., Guillemin, G.J., and Brew, B.J. (2017). Bcl11b-A critical neurodevelopmental transcription factor-roles in health and disease. Front. Cell. Neurosci. *11*, 89.
67. Zhang, J., Ding, L., Holmfeldt, L., Wu, G., Heatley, S.L., Payne-Turner, D., Easton, J., Chen, X., Wang, J., Rusch, M., et al. (2012). The genetic basis of early T-cell precursor acute lymphoblastic leukaemia. Nature *481*, 157–163.
68. Booth, C.A.G., Barkas, N., Neo, W.H., Boukarabila, H., Soilleux, E.J., Giotopoulos, G., Farnoud, N., Giustacchini, A., Ashley, N., Carrelha, J., et al. (2018). Ezh2 and Runx1 mutations collaborate to initiate lympho-myeloid leukemia in early thymic progenitors. Cancer Cell *33*, 274–291.e8.
69. Johnson, J.L., Georgakilas, G., Petrovic, J., Kurachi, M., Cai, S., Harly, C., Pear, W.S., Bhandoola, A., Wherry, E.J., and Vahedi, G. (2018). Lineage-determining transcription factor TCF-1 initiates the epigenetic identity of T cells. Immunity *48*, 243–257.e10.
70. Canté-Barrett, K., Pieters, R., and Meijerink, J.P.P. (2014). Myocyte enhancer factor 2C in hematopoiesis and leukemia. Oncogene *33*, 403–410.
71. Homminga, I., Pieters, R., Langerak, A.W., de Rooi, J.J., Stubbs, A., Verstegen, M., Vuerhard, M., Buijs-Gladdines, J., Kooi, C., Klous, P., et al. (2011). Integrated transcript and genome analyses reveal NKX2-1 and MEF2C as potential oncogenes in T cell acute lymphoblastic leukemia. Cancer Cell *19*, 484–497.
72. Nagel, S., Venturini, L., Meyer, C., Kaufmann, M., Scherr, M., Drexler, H.G., and Macleod, R.A.F. (2011). Transcriptional deregulation of oncogenic myocyte enhancer factor 2C in T-cell acute lymphoblastic leukemia. Leuk. Lymphoma *52*, 290–297.
73. De Bie, J., Demeyer, S., Alberti-Servera, L., Geerdens, E., Segers, H., Broux, M., De Keersmaecker, K., Michaux, L., Vandenberghe, P., Voet, T., et al. (2018). Single-cell sequencing reveals the origin and the order of mutation acquisition in T-cell acute lymphoblastic leukemia. Leukemia *32*, 1358–1369.
74. Albertí-Servera, L., Demeyer, S., Govaerts, I., Swings, T., De Bie, J., Gielen, O., Brociner, M., Michaux, L., Maertens, J., Uyttebroeck, A., et al. (2021). Single-cell DNA amplicon sequencing reveals clonal heterogeneity and evolution in T-cell acute lymphoblastic leukemia. Blood *137*, 801–811.
75. Zuurbier, L., Gutierrez, A., Mullighan, C.G., Canté-Barrett, K., Gevaert, A.O., de Rooi, J., Li, Y., Smits, W.K., Buijs-Gladdines, J.G.C.A.M., Sonneveld, E., et al. (2014). Immature MEF2C-dysregulated T-cell leukemia patients have an early T-cell precursor acute lymphoblastic leukemia gene signature and typically have non-rearranged T-cell receptors. Haematologica *99*, 94–102.
76. Colomer-Lahiguera, S., Pisecker, M., König, M., Nebral, K., Pickl, W.F., Kauer, M.O., Haas, O.A., Ullmann, R., Attarbaschi, A., Dworzak, M.N., and Strehl, S. (2017). MEF2C-dysregulated pediatric T-cell acute lymphoblastic leukemia is associated with CDKN1B deletions and a poor response to glucocorticoid therapy. Leuk. Lymphoma *58*, 2895–2904.
77. Braun, C.J., Adames, A.C., Saur, D., and Rad, R. (2022). Tutorial: design and execution of CRISPR in vivo screens. Nat. Protoc. *17*, 1903–1925.
78. Haydu, J.E., and Ferrando, A.A. (2013). Early T-cell precursor acute lymphoblastic leukaemia. Curr. Opin. Hematol. *20*, 369–373.
79. Gutierrez, A., Sanda, T., Grebliunaite, R., Carracedo, A., Salmena, L., Ahn, Y., Dahlberg, S., Neuberg, D., Moreau, L.A., Winter, S.S., et al. (2009). High frequency of PTEN, PI3K, and AKT abnormalities in T-cell acute lymphoblastic leukemia. Blood *114*, 647–650.
80. MacArthur, J., Bowler, E., Cerezo, M., Gil, L., Hall, P., Hastings, E., Junkins, H., McMahon, A., Milano, A., Morales, J., et al. (2017). The new NHGRI-EBI Catalog of published genome-wide association studies (GWAS Catalog). Nucleic Acids Res. *45*. D896-d901.
81. Adams, D.J., Biggs, P.J., Cox, T., Davies, R., van der Weyden, L., Jonkers, J., Smith, J., Plumb, B., Taylor, R., Nishijima, I., et al. (2004). Mutagenic insertion and chromosome engineering resource (MICER). Nat. Genet. *36*, 867–871.
82. Bredthauer, C., Fischer, A., Ahari, A.J., Cao, X., Weber, J., Rad, L., Rad, R., Wachutka, L., and Gagneur, J. (2023). Transmicron: accurate prediction of insertion probabilities improves detection of cancer driver genes from transposon mutagenesis screens. Nucleic Acids Res., gkac1215.
83. Heinz, S., Benner, C., Spann, N., Bertolino, E., Lin, Y.C., Laslo, P., Cheng, J.X., Murre, C., Singh, H., and Glass, C.K. (2010). Simple combinations of lineage-determining transcription factors prime cis-regulatory elements required for macrophage and B cell identities. Mol. Cell *38*, 576–589.
84. Ernst, J., and Kellis, M. (2012). ChromHMM: automating chromatin-state discovery and characterization. Nat. Methods *9*, 215–216.
85. Subramanian, A., Tamayo, P., Mootha, V.K., Mukherjee, S., Ebert, B.L., Gillette, M.A., Paulovich, A., Pomeroy, S.L., Golub, T.R., Lander, E.S., and Mesirov, J.P. (2005). Gene set enrichment analysis: a knowledge-based approach for interpreting genome-wide expression profiles. Proc. Natl. Acad. Sci. USA *102*, 15545–15550.
86. Gu, Z., Schlesner, M., and Hübschmann, D. (2021). cola: an R/Bioconductor package for consensus partitioning through a general framework. Nucleic Acids Res. *49*, e15.
87. Falcomatà, C., Bärthel, S., Ulrich, A., Diersch, S., Veltkamp, C., Rad, L., Boniolo, F., Solar, M., Steiger, K., Seidler, B., et al. (2021). Genetic screens identify a context-specific PI3K/p27Kip1 node driving extrahepatic biliary cancer. Cancer Discov. *11*, 3158–3177.
88. Schick, M., Zhang, L., Maurer, S., Maurer, H.C., Isaakaidis, K., Schneider, L., Patra, U., Schunck, K., Rohleder, E., Hofstetter, J., et al. (2022). Genetic alterations of the SUMO isopeptidase SENP6 drive lymphomagenesis and genetic instability in diffuse large B-cell lymphoma. Nat. Commun. *13*, 281.
89. Morse, H.C., 3rd, Anver, M.R., Fredrickson, T.N., Haines, D.C., Harris, A.W., Harris, N.L., Jaffe, E.S., Kogan, S.C., MacLennan, I.C.M., Pattengale, P.K., et al. (2002). Bethesda proposals for classification of lymphoid neoplasms in mice. Blood *100*, 246–258.
90. Quinlan, A.R., Clark, R.A., Sokolova, S., Leibowitz, M.L., Zhang, Y., Hurles, M.E., Mell, J.C., and Hall, I.M. (2010). Genome-wide mapping and assembly of structural variant breakpoints in the mouse genome. Genome Res. *20*, 623–635.
91. Ramírez, F., Dündar, F., Diehl, S., Grüning, B.A., and Manke, T. (2014). deepTools: a flexible platform for exploring deep-sequencing data. Nucleic Acids Res. *42*, W187–W191.
92. Bailey, M.H., Tokheim, C., Porta-Pardo, E., Sengupta, S., Bertrand, D., Weerasinghe, A., Colaprico, A., Wendl, M.C., Kim, J., Reardon, B., et al. (2018). Comprehensive characterization of cancer driver genes and mutations. Cell *173*, 371–385.e18.
93. Reddy, A., Zhang, J., Davis, N.S., Moffitt, A.B., Love, C.L., Waldrop, A., Leppa, S., Pasanen, A., Meriranta, L., Karjalainen-Lindsberg, M.L., et al. (2017). Genetic and functional drivers of diffuse large B cell lymphoma. Cell *171*, 481–494.e15.
94. Tyner, J.W., Tognon, C.E., Bottomly, D., Wilmot, B., Kurtz, S.E., Savage, S.L., Long, N., Schultz, A.R., Traer, E., Abel, M., et al. (2018). Functional genomic landscape of acute myeloid leukaemia. Nature *562*, 526–531.

95. Zhang, J., McCastlain, K., Yoshihara, H., Xu, B., Chang, Y., Churchman, M.L., Wu, G., Li, Y., Wei, L., Iacobucci, I., et al. (2016). Deregulation of DUX4 and ERG in acute lymphoblastic leukemia. Nat. Genet. *48*, 1481–1489.

96. (2020). Pan-cancer analysis of whole genomes. Nature *578*, 82–93.

97. Nguyen, B., Fong, C., Luthra, A., Smith, S.A., DiNatale, R.G., Nandakumar, S., Walch, H., Chatila, W.K., Madupuri, R., Kundra, R., et al. (2022). Genomic characterization of metastatic patterns from prospective clinical sequencing of 25,000 patients. Cell *185*, 563–575.e11.

98. Ing-Simmons, E., Seitan, V.C., Faure, A.J., Flicek, P., Carroll, T., Dekker, J., Fisher, A.G., Lenhard, B., and Merkenschlager, M. (2015). Spatial enhancer clustering and regulation of enhancer-proximal genes by cohesin. Genome Res. *25*, 504–513.

99. Wei, G., Abraham, B.J., Yagi, R., Jothi, R., Cui, K., Sharma, S., Narlikar, L., Northrup, D.L., Tang, Q., Paul, W.E., et al. (2011). Genome-wide analyses of transcription factor GATA3-mediated gene regulation in distinct T cell types. Immunity *35*, 299–311.

100. Sidoli, S., Lopes, M., Lund, P.J., Goldman, N., Fasolino, M., Coradin, M., Kulej, K., Bhanu, N.V., Vahedi, G., and Garcia, B.A. (2019). A mass spectrometry-based assay using metabolic labeling to rapidly monitor chromatin accessibility of modified histone proteins. Sci. Rep. *9*, 13613.

101. Yue, F., Cheng, Y., Breschi, A., Vierstra, J., Wu, W., Ryba, T., Sandstrom, R., Ma, Z., Davis, C., Pope, B.D., et al. (2014). A comparative encyclopedia of DNA elements in the mouse genome. Nature *515*, 355–364.

102. Lara-Astiaso, D., Weiner, A., Lorenzo-Vivas, E., Zaretsky, I., Jaitin, D.A., David, E., Keren-Shaul, H., Mildner, A., Winter, D., Jung, S., et al. (2014). Immunogenetics. Chromatin state dynamics during blood formation. Science *345*, 943–949.

103. Shih, H.Y., Sciumè, G., Mikami, Y., Guo, L., Sun, H.W., Brooks, S.R., Urban, J.F., Jr., Davis, F.P., Kanno, Y., and O'Shea, J.J. (2016). Developmental acquisition of regulomes underlies innate lymphoid cell functionality. Cell *165*, 1120–1133.

104. Yoshida, H., Lareau, C.A., Ramirez, R.N., Rose, S.A., Maier, B., Wroblewska, A., Desland, F., Chudnovskiy, A., Mortha, A., Dominguez, C., et al. (2019). The cis-regulatory atlas of the mouse immune system. Cell *176*, 897–912.e20.

105. Lawrence, M., Huber, W., Pagès, H., Aboyoun, P., Carlson, M., Gentleman, R., Morgan, M.T., and Carey, V.J. (2013). Software for computing and annotating genomic ranges. PLoS Comput. Biol. *9*, e1003118.

106. Fulco, C.P., Nasser, J., Jones, T.R., Munson, G., Bergman, D.T., Subramanian, V., Grossman, S.R., Anyoha, R., Doughty, B.R., Patwardhan, T.A., et al. (2019). Activity-by-contact model of enhancer-promoter regulation from thousands of CRISPR perturbations. Nat. Genet. *51*, 1664–1669.

107. Bouvy-Liivrand, M., Hernández de Sande, A., Pölönen, P., Mehtonen, J., Vuorenmaa, T., Niskanen, H., Sinkkonen, L., Kaikkonen, M.U., and Heinäniemi, M. (2017). Analysis of primary microRNA loci from nascent transcriptomes reveals regulatory domains governed by chromatin architecture. Nucleic Acids Res. *45*, 12054.

108. Leong, W.Z., Tan, S.H., Ngoc, P.C.T., Amanda, S., Yam, A.W.Y., Liau, W.S., Gong, Z., Lawton, L.N., Tenen, D.G., and Sanda, T. (2017). ARID5B as a critical downstream target of the TAL1 complex that activates the oncogenic transcriptional program and promotes T-cell leukemogenesis. Genes Dev. *31*, 2343–2360.

109. Knoechel, B., Roderick, J.E., Williamson, K.E., Zhu, J., Lohr, J.G., Cotton, M.J., Gillespie, S.M., Fernandez, D., Ku, M., Wang, H., et al. (2014). An epigenetic mechanism of resistance to targeted therapy in T cell acute lymphoblastic leukemia. Nat. Genet. *46*, 364–370.

110. Michel, B.C., D'Avino, A.R., Cassel, S.H., Mashtalir, N., McKenzie, Z.M., McBride, M.J., Valencia, A.M., Zhou, Q., Bocker, M., Soares, L.M.M., et al. (2018). A non-canonical SWI/SNF complex is a synthetic lethal target in cancers driven by BAF complex perturbation. Nat. Cell Biol. *20*, 1410–1420.

111. Kwiatkowski, N., Zhang, T., Rahl, P.B., Abraham, B.J., Reddy, J., Ficarro, S.B., Dastur, A., Amzallag, A., Ramaswamy, S., Tesar, B., et al. (2014). Targeting transcription regulation in cancer with a covalent CDK7 inhibitor. Nature *511*, 616–620.

112. Manser, M., Sater, M.R.A., Schmid, C.D., Noreen, F., Murbach, M., Kuster, N., Schuermann, D., and Schär, P. (2017). ELF-MF exposure affects the robustness of epigenetic programming during granulopoiesis. Sci. Rep. *7*, 43345.

113. Lucic, B., Chen, H.C., Kuzman, M., Zorita, E., Wegner, J., Minneker, V., Wang, W., Fronza, R., Laufs, S., Schmidt, M., et al. (2019). Spatially clustered loci with multiple enhancers are frequent targets of HIV-1 integration. Nat. Commun. *10*, 4059.

114. Kaikkonen, M.U., Spann, N.J., Heinz, S., Romanoski, C.E., Allison, K.A., Stender, J.D., Chun, H.B., Tough, D.F., Prinjha, R.K., Benner, C., and Glass, C.K. (2013). Remodeling of the enhancer landscape during macrophage activation is coupled to enhancer transcription. Mol. Cell *51*, 310–325.

115. Parekh, S., Ziegenhain, C., Vieth, B., Enard, W., and Hellmann, I. (2016). The impact of amplification on differential expression analyses by RNA-seq. Sci. Rep. *6*, 25533.

116. Macosko, E.Z., Basu, A., Satija, R., Nemesh, J., Shekhar, K., Goldman, M., Tirosh, I., Bialas, A.R., Kamitaki, N., Martersteck, E.M., et al. (2015). Highly parallel genome-wide expression profiling of individual cells using nanoliter droplets. Cell *161*, 1202–1214.

117. Love, M.I., Huber, W., and Anders, S. (2014). Moderated estimation of fold change and dispersion for RNA-seq data with DESeq2. Genome Biol. *15*, 550.

118. Laurenti, E., Doulatov, S., Zandi, S., Plumb, I., Chen, J., April, C., Fan, J.B., and Dick, J.E. (2013). The transcriptional architecture of early human hematopoiesis identifies multilevel control of lymphoid commitment. Nat. Immunol. *14*, 756–763.

119. Novershtern, N., Subramanian, A., Lawton, L.N., Mak, R.H., Haining, W.N., McConkey, M.E., Habib, N., Yosef, N., Chang, C.Y., Shay, T., et al. (2011). Densely interconnected transcriptional circuits control cell states in human hematopoiesis. Cell *144*, 296–309.

120. Zhu, A., Ibrahim, J.G., and Love, M.I. (2019). Heavy-tailed prior distributions for sequence count data: removing the noise and preserving large differences. Bioinformatics *35*, 2084–2092.

## STAR★METHODS

### KEY RESOURCES TABLE

| REAGENT or RESOURCE | SOURCE | IDENTIFIER |
|---|---|---|
| **Antibodies** | | |
| rat anti-B220/CD45R | BD Bioscience | B220; AB_393581 |
| rat anti-CD138 | BD Bioscience | 281–2; RRID:AB_394999 |
| rat anti-MPO | DAKO | A0398 |
| rabbit anti-CD3 | DCS | Sp7; RRID:AB_2864584 |
| rabbit anti-Tdt | Supertechs | 005 |
| rat anti-CD4 | Dianova | GHH4; RRID:AB_2800530 |
| Rabbit anti-rat secondary antibody | Vector | AI-4001-.5 |
| **Critical commercial assays** | | |
| Amaxa® Cell Line Nucleofector® Kit V | Lonza Bioscience | Kit V |
| Amaxa® Cell Line Nucleofector® Kit V | Lonza Bioscience | Kit L |
| **Deposited data** | | |
| Publicly available data | See Tables S5 and S6 | NA |
| Deposited human GRO-Seq data | This paper | EGAS00001005864 |
| Deposited murine RNA-seq data | This paper | PRJEB59121 |
| GWAS Catalog (v1.0.2) | MacArthur et al.[80] | RRID:SCR_012745 |
| **Experimental models: Cell lines** | | |
| Jurkat | ATCC | TIB-152, RRID:CVCL_0065 |
| EL4 | ATCC | TIB-39, RRID:CVCL_0255 |
| HEK293T | ATCC | CRL-3216, RRID:CVCL_0063 |
| C2a_16990 | Müller et al.[10] | NA |
| C1_9091 | Müller et al.[10] | NA |
| **Experimental models: Organisms/strains** | | |
| *Rosa26$^{PB}$* | Rad et al.[37] | *Rosa26$^{PB}$* |
| *ATP2* | Rad et al.[37] | *ATP2* |
| *Bcl11b$^{\Delta 1Mb}$* | This paper | NA |
| *Bcl11b$^{\Delta 105kb}$* | This paper | NA |
| **Oligonucleotides** | | |
| Oligonucleotides | See Table S19 | NA |
| **Recombinant DNA** | | |
| pX333 vector | Addgene | RRID:Addgene_64073 |
| lentiCas9-Blast | Addgene | RRID:Addgene_52962 |
| guide-GFP vector | Addgene | RRID:Addgene_57822 |
| MICER targeting vectors | Adams et al.[81] | NA |
| **Software and algorithms** | | |
| QiSeq | Friedrich et al.[40] | NA |
| CIMPL | de Ridder et al.[41] | NA |
| Transmicron | Bredthauer et al.[82] | NA |
| HOMER (v4.11) | Heinz et al.[83] | RRID:SCR_010881 |
| chromHMM | Ernst and Kellis[84] | RRID:SCR_018141 |
| GSEA v4.0.3 | Subramanian et al.[85] | RRID:SCR_003199 |
| LDlink | Machiela and Chanock[60] | NA |
| GenomicRanges | Bioconductor | RRID:SCR_000025 |
| DESeq2 | Bioconductor | RRID:SCR_015687 |

(*Continued on next page*)

| Continued | | |
|---|---|---|
| REAGENT or RESOURCE | SOURCE | IDENTIFIER |
| cola | Gu et al.[86] | NA |
| Other | | |
| UCSC Genome Browser | http://genome.ucsc.edu/ | RRID:SCR_005780 |
| Inkscape | https://inkscape.org/en/ | RRID:SCR_014479 |

## RESOURCE AVAILABILITY

### Lead contact

Further information and requests for resources and reagents should be directed to and will be fulfilled by the lead contact, Roland Rad (roland.rad@tum.de).

### Materials availability

This study did not generate new unique reagents. Mouse lines generated in this study are available from the lead contact upon request.

### Data and code availability

Murine RNA-seq raw data are deposited at EBI European Nucleotide Archive under the accession number PRJEB59121. Human raw GRO-Seq data are deposited at European Genome-Phenome Archive EGA under the accession number EGAS00001005864.

Accession numbers are listed in the key resources table and Table S1. This paper additionally analyzes existing, publicly available data. Accession numbers are also listed in the key resources table and Tables S5 and S6.

Any additional information required to reanalyze the data reported in this paper is available from the lead contact upon request.

## EXPERIMENTAL MODEL AND SUBJECT DETAILS

### Mouse strains

Constitutive *PiggyBac* (PB) transposase knock-in mice (*Rosa26$^{PB}$*) and transgenic transposon mouse lines harboring *ATP2* have been described earlier.[37] Experimental (*Rosa26$^{PB/+}$;ATP2*) and control (*Rosa26$^{PB/+}$* and *ATP2* single transgenic) mice were maintained on a mixed C57BL/6 x 129Sv x FVB background. The T-ALL cohort included 48 *ATP2-S1*, 2 *ATP2-H27* and 1 *ATP2-H32* mice (information on sex and age listed in Table S1).

We generated intergenic knockout mouse models using MICER targeting vectors as previously described.[81] Mouse embryonic stem (ES) cells were transfected by electroporation and those carrying the vector were selected. Experimental mice were maintained on a mixed C57BL/6 x 129Sv x FVB background (information on sex and age listed in Table S12).

Mice were kept in the animal facilities of the Wellcome Trust Sanger Institute, Hinxton/Cambridge, UK under specific-pathogen-free (SPF) conditions on a 12-h light/dark cycle, receiving food and water *ad libitum*. All animal experiments were carried out in compliance with the requirements of the European guidelines for the care and use of laboratory animals and were approved by the UK Home Office and the Institutional Animal Care and Use Committees (IACUC). Genotyping primers are listed in Table S19.

### Cell lines

The human T-ALL cell line Jurkat (ATCC® TIB-152™, male) and the murine T cell lymphoma cell line EL4 (ATCC® TIB-39™, sex not reported) were used for knockout and HEK293T cells (ATCC® CRL-3216™, female) were used as a control. All cell lines were cultivated according to distributor's instructions. Additionally, primary murine pancreatic cancer cell lines were used from Müller et al.[10] Both pancreatic cell lines, the C2a cell line (16990, male) and the C1 cell line (9091, female), were cultivated in DMEM. All cell lines were cultured in media supplemented with fetal bovine serum (FBS, 10%) and 1% penicillin/streptomycin and maintained at 37°C with 5% $CO_2$.

### Human subjects

Primary bone marrow samples from two pediatric T-ALL patients (both male) were used for GRO-Seq assay. The study was approved by the Regional Ethics Committee in Pirkanmaa, Tampere, Finland (#R13109) and was conducted according to the guidelines of the Declaration of Helsinki, and a written informed consent was received by the patient and/or guardians.

## METHOD DETAILS

### Generation of mouse strains and cohorts

Constitutive *PiggyBac* (PB) transposase knock-in mice (*Rosa26$^{PB}$*) and transgenic transposon mouse lines harboring *ATP2* have been described earlier.[37] Experimental (*Rosa26$^{PB/+}$;ATP2*) and control (*Rosa26$^{PB/+}$* and *ATP2* single transgenic) mice were

maintained on a mixed C57BL/6 x 129Sv x FVB background. Different ATP2 lines were used to generate final cohorts, which differ in their number of transposon copies and the donor locus (*ATP2-S1*: donor locus chr17, 15 copies; *ATP2-H27*: donor locus chr4, 20 copies; *ATP2-H32*: donor locus chr2, 25 copies). The T-ALL cohort included 48 *ATP2-S1*, 2 *ATP2-H27* and 1 *ATP2-H32* mice (Table S1) In addition to in depth analyses of tumors originating from these mouse cohorts, we have also explored specific questions (on regulatory CISs) in datasets emanating from a large number of – mostly so far unpublished - constitutive and conditional *PiggyBac* screens[38,87,88] (Table S13). In total we analyzed 1450 tumors from 15 screens. Additionally, for comparison of integration profiles, we analyzed a cohort of hematologic malignancies (n = 11) derived from a whole-body *Sleeping Beauty* Screen.[82]

We generated intergenic knockout mouse models using MICER targeting vectors as previously described.[81] For the 1 Mb deletion, the MHPN-250E23 and the MHPP-53N24 and for the 105 kb deletion, the MHPN-262H24 and the MHPP-53N24 targeting vectors from the MICER 3'Hprt (MHPP) and 5'Hprt (MHPN) library (CloneDB database) were used. Mouse embryonic stem (ES) cells were transfected by electroporation and those carrying the vector were selected. After transient Cre expression, the *Hprt* minigene recombines in ES cells carrying both vectors and mediates hypoxanthine/aminopterin/thymidine (HAT) resistance. ES cells carrying the deletion were selected with HAT-Medium and injected into C57BL/6-derived blastocysts to generate the mice. Experimental mice were maintained on a mixed C57BL/6 x 129Sv x FVB background.

## Necropsy and histopathological analysis

All animals were monitored regularly for signs of sickness (e.g., inactivity, palpable masses and poor grooming). During necropsy, a gross inspection of all internal organs was carried out. For DNA/RNA isolation, tissue samples were stored in RNAlater (Sigma). For histology, tissue samples were fixed in 4% formaldehyde, paraffin-embedded, sectioned, and stained using hematoxylin and eosin following standard protocols.

## Immunohistochemistry

Immunohistochemistry (IHC) was performed on a Bond Rxm (Leica) using a Polymer Refine detection kit without post-primary antibody. Slides were deparaffinized and pretreated with Epitope retrieval solution 1 (ER1, citrate buffer, pH = 6) or solution 2 (ER2, EDTA buffer, pH = 9) as indicated. The following primary antibodies were used: rat anti-B220/CD45R (B220, BD Bioscience, 1:50 dilution, ER1, 20 min), rat anti-CD138 (281-2, BD Bioscience, 1:59, ER2, 20 min), rat anti-MPO (A0398, DAKO, 1:100, ER2, 20 min), rabbit anti-CD3 (Sp7, DCS, 1:100 dilution, ER1, 20 min), rabbit anti-Tdt (005, Supertechs, 1:100, ER2, 20 min) and rat anti-CD4 (GHH4, Dianova DIA-404, 1:50 dilution, ER2, 40 min). Rabbit anti-rat secondary antibody (Vector, 1:400) was applied for primary rat antibodies. Slides were counterstained with hematoxylin and coverslipped after manual rehydration. Slides were scanned with a Leica AT2 scanning system. HE stainings and IHCs were evaluated by experienced mouse pathologists, who were blinded to the mouse genotypes according to the Bethesda proposals for classification of lymphoid neoplasms.[89]

## Quantitative transposon insertion site sequencing

QiSeq is a method for semi-quantitative transposon insertion sequencing that we developed earlier.[40] Briefly, DNA samples were sheared with a Covaris AFA sonicator to a mean fragment length of 250 bp. The fragmented DNA was then end-repaired, A-tailed and a splinkerette adapter was ligated to each DNA end. For the 5' and 3' transposon end, subsequent steps (amplification and sequencing of transposon-genome junctions) were conducted separately. The specific structure of the splinkerette adapter (Y-shaped design with a template and a hairpin strand) ensures that only transposon-genome junction fragments (and not genomic fragments without transposon insert) can be amplified in the first PCR step (which was conducted with transposon- and splinkerette-specific primers). Afterwards, a second nested PCR step was performed for further amplification, barcoding of samples and extension with Illumina flow cell binding sites P5 and P7. Each sample was then quantified with quantitative real-time PCR (using P5- and P7-specific primers). Subsequently, samples were equimolarly mixed and the library pool was again quantified. Libraries were sequenced on the Illumina MiSeq sequencer (75 bp, paired-end). Mapping of integrations to the mouse genome (mm10) was performed using the SSAHA2 algorithm and sequences containing transposon-genome junctions were selected for downstream analyses. Read coverage of insertions was normalized to the top hit of each sample (normalized read coverage).

## CIS calling and downstream analyses

For the identification of common insertion sites (CISs; genomic regions that are more frequently hit by transposons than expected by chance), ATP2 insertions were subjected to statistical analysis using CIMPL (Common Insertion site Mapping PLatform),[41] which is based on a Gaussian kernel convolution framework. CIMPL assigns a p value to each CIS (listed in Table S2) and controls the errors at an average of 5%. Insertions within a 3 Mb region upstream and downstream of the transposon donor locus were excluded from the analysis (local hopping area of the transposon as described in[37]). CISs were ranked according to the number of contributing insertions. *Sfi1*, a known artefact frequently detected in insertional mutagenesis screens, was removed from the list of CIS genes.[90] Additionally, *Arid1b* and *Mmp16* were excluded due to their close proximity to the donor locus on chr17 and chr4, respectively. CIS genes for Table S2 were ranked according to the number of contributing samples. A scale parameter of 30 k was used for CIS identification. Profile plots and profile heatmap plots for visualization of ChIP-Seq peak enrichment in CIS regions were created using deeptools.[91] Subgroup specific CIS analysis were performed using a scale parameter of 5 k and were ranked according to the number of contributing insertions. For comparisons of the number of intergenic CISs in multiple cancer types, a scale parameter of 5 k was used. The

GENCODE Biotype annotation was used to differentiate genic (protein-coding and immunoglobulin genes) and intergenic (all other biotypes) CISs. CIS genes were compared to human cancer sequencing studies[92–97](Table S3).

To exclude the possibility that insertion biases rather than selection are underlying non-coding CISs accumulation, we also used Transmicron[82] for CIS calling, which corrects for insertion biases by modelling neutral insertion rates based on chromatin accessibility, transcriptional activity, and sequence context. CISs were filtered for an adjusted p value < 0.05 and overlapped with CIMPL 5 k CISs. Of the 537 evaluated CIMPL-CISs (Table S8) and the top-ranked CIMPL-CISs (Figure 2E), 79% and 84% were also called by Transmicron, respectively.

### Footprint plots and transcription factor motif search

Footprint plots were generated using intergenic T-ALL CISs (n = 227) as an input. To show the specificity of the footprint signature, a random background model was generated. Instead of selecting arbitrary loci in the genome, a set of "control CISs" was generated with specific characteristics as similar as possible to the original CISs. For each CIS, the chromosome, width, number of insertions as well as the number of comprised TTAA positions was used to identify a matching region in the genome resulting in a large pool of "control CISs" for each original CIS. "Control CISs" overlapping with any CIS were removed. The peak position for each mimicry-CIS was determined by the relative position of the peak in the original CIS. The procedure of identifying the overlap density with annotated regions was identical for the original and the control data, whereby for the latter this step was repeated 100 times in a bootstrapping approach. Every time one randomly selected "control CIS" from the previously generated pool of candidates was selected, and the final density line was then generated based on the 97.5% quantile of all values. ChIP-Seq input files are listed in Table S5. For analysis of transcription factor binding sites, regions flanking 200 bp of single intergenic insertions sites were used as input. The findMotifsGenome.pl tool of HOMER (v4.11)[83] was used for known motif analysis using default parameter.

### CIS annotation pipeline (ARCIS)

For the identification of CISs using CIMPL, the scale parameter was set to 5 k to identify narrow regions with regulatory potential (Figures 2A, 2B, and S5A). The resulting CIS coordinates were overlapped with a collection of publicly available datasets listed in Table S5 [43,44,69,98–104] using the GenomicRanges R package.[105] The data was post-processed into a BED3 format with an additional column for name assignment. For overlap with peak-based files (ChIP-Seq, DNase-Seq), the number of overlapping peaks and the distance to the closest peak are reported. For interaction datasets (Hi-C from different stages of development and data from dbSuper [mouse thymus]), linked target genes are annotated. Chromatin regions specifically increasing or decreasing during T cell development (change in A and B compartment scores called from Hi-C data in Hu et al.[43]) were also overlapped with CIS regions (all publicly available datasets are listed in Table S5, detailed ARCIS scheme shown in Figure S5B).

Additionally, we run a chromatin Hidden Markov Model (chromHMM)[84] on a collection of thymus ChIP-Seq data from ENCODE to define chromatin states, based on distinct combinations of histone marks. Chromatin states were used as an additional input dataset for ARCIS. We used six thymus-specific ChIP-Seq datasets: H3K4me1, H3K27ac, H3K4me3, H3K27me3, H3K36me3 and CTCF. The observed chromatin combinations resulted in eight manually assigned chromatin states: active/weak/poised/insulated enhancer, active promotor, gene body, CTCF binding sites and quiescent (Figure S5C, Table S7). For the human T-ALL cell lines DND41 and Jurkat chromatin states are shown in Figure S7. The predicted regions showed a median range of 400-1000 bp. To overcome the issue of genomic sections with various small interrupted states, we implemented a merging and smoothing step. For each chromatin state, neighboring regions within 3000 bp of each other were merged into a single larger region, while bridging other states in between. Resulting smooth chromatin states were filtered to only include regions with a minimum size of 4000 bp. As an additional attempt to obtain large and coherent regulatory elements, the same procedure was also applied to combinations of interrelated chromatin states in close distance, like active with weak enhancers as well as promoters with active enhancers. Regarding the many different states, only active and weak enhancer chromatin states were used for CIS annotation. The number of overlaps as well as the distance to the closest element is reported. As silencers are less well-studied, and CISs affecting insulators were rare, we focused our downstream analyses on enhancers and ncRNAs.

ARCIS computes the overlap between CIS regions and all input datasets and reports number of observed overlaps as well as the putative target gene. For each CIS, ARCIS reports: (i) transcript annotation, (ii) number of peaks or distance to the closest peak (for ChIP-, ATAC- and DNase-Seq), (iii) intersection with super-enhancers, (iv) connected target gene (for Hi-C and dbSuper), (v) information on chromatin access change during T cell development, and (vi) annotation from chromHMM (Table S7). ARCIS calculates a 'RE-score' based on a combination of features and ranks the CISs according to their regulatory potential reflecting an additional layer of information to support data interpretation (details shown in Figure S6). In brief, overlaps with super-enhancers (dbSuper), increasing chromatin accessibility, active/weak enhancers according to chromHMM data and Hi-C connections were used for scoring. The ARCIS output comprises a full (Table S7) as well as a user-friendly "reduced" format (Table S8).

The classification of intergenic enhancers without overlap to other functional elements is straightforward. However, because functional elements in the genome often overlap (e.g. enhancer overlapping with lncRNAs and/or mRNAs) or are found in close proximity to each other, a substantial part of putative REs has to be further inspected individually (Figures 2E and S6).

Main criteria for discriminating different RE categories are the position of CIS-insertions in relation to potential overlapping/neighboring functional units in the genome, their orientation as well as the pattern of insertion clusters across samples. Integrating lncRNA and mRNA expression profiles in respective tissues further aids discrimination of the RE type targeted by transposons in regions with

multiple overlapping functional elements (Figure S5D). In a subset of these cases, however, definitive classification is not possible, but requires downstream experimental interrogation. A detailed description of the manual annotation algorithm as well as related decision trees are provided in Figure S6.

ARCIS can be used for any insertional mutagenesis screen as the availability of chromatin accessibility and histone modification data constantly increases. If no tissue-specific data is available (especially for Hi-C) there are efforts to create global datasets that can be applied universally.[106]

Human ARCIS was performed with datasets listed in Table S6.[27,29,107–113]

## GRO-Seq

Primary bone marrow samples from two pediatric T-ALL patients were used for GRO-Seq assay. The study was approved by the Regional Ethics Committee in Pirkanmaa, Tampere, Finland (#R13109) and was conducted according to the guidelines of the Declaration of Helsinki, and a written informed consent was received by the patient and/or guardians. In addition, the T-ALL cell line Jurkat was included (from the Leibniz-Institut DSMZ-Deutsche Sammlung von Mikroorganismen und Zellkulturen GmbH, Germany). The nuclei isolation and GRO-Seq library construction was performed as previously described,[114] yielding $\sim$1–5$\times$10$^6$ nuclei per condition. Single-end Illumina sequencing was performed by GeneCore EMBL, Heidelberg Germany. GRO-Seq reads were trimmed using the HOMER (http://homer.salk.edu/homer) software (homerTools trim) to remove A-stretches originating from the library preparation. From the resulting sequences, those shorter than 25 bp or with poor quality were discarded. Genome alignment with Bowtie was done in two steps, first removing reads mapping to rRNA regions (AbundantSequences as annotated by iGenomes) and blacklisted regions (unusual low or high mappability as defined by ENCODE) followed by alignment to hg19. Up to two mismatches and up to three locations were accepted per read and the best alignment was reported. For visualization, reads were normalized to 107 reads to generate bedGraph and bigWig files using HOMER. GRO-Seq data for HEK293T cells were published previously[107] (Table S6).

## Cell-culture-based CRISPR-Cas9 knockout experiments

For *in vitro* knockout experiments of candidate regions, region specific guides or *lacZ* guide controls (sequences listed in Table S19) were sequentially cloned into the pX333 vector for expression of two sgRNAs from two independent U6 promoters and Cas9 expression by the *Cbh* promoter (Addgene #64073). For each knockout experiment, six guides were selected (three on each site of the knockout region). Vectors of different guide combinations were pooled before electroporation. The human T-ALL cell line Jurkat (ATCC® TIB-152™) and the murine T cell lymphoma cell line EL4 (ATCC® TIB-39™) were used for knockout and HEK293T cells (ATCC® CRL-3216™) were used as a control. All cell lines were cultivated according to distributor's instructions. Cell lines were electroporated using the Amaxa® Cell Line Nucleofector® Kit V and Kit L (Lonza Bioscience). For each knockout, the pX333 vector and a GFP vector were co-electroporated into 2 $\times$ 10$^6$ cells according to manufacturer's protocol. For *Nrip1* and *Enpp1* knockout in pancreatic cancer, a C2a cell line (16990) and a C1 cell line (9091) from Müller et al.[10] were used, respectively. Cells were first transduced with a lentiCas9-Blast vector (Addgene #52962). Stable single-cell derived Cas9-expressing clones were transfected with a guide-GFP vector (Addgene #57822) using Lipofectamine 3000 (Thermo Fisher Scientific) according to manufacturer's instructions. Here, two guides were used on each site of the knockout region. GFP positive cells were single-cell sorted in 96-well plates and cultured with conditioned medium. Colonies grown from single cell clones were screened for the knockout using PCR with region specific primers (Table S19). Positive clones were expanded for RNA isolation. Expression of the target gene was determined by real-time quantitative PCR (qPCR) using primers specific for the target transcripts (Table S19). For normalization of RNA input, *Gapdh* qPCR (Table S19) was performed. Expression of the target gene was compared to cell clones electroporated with *lacZ* guides.

## DNA and RNA isolation

DNA and total RNA isolation of tissue samples and cell clones was performed according to manufacturer's instructions using the Qiagen DNeasy Blood & Tissue Kit, the Qiagen RNeasy Plus Mini Kit or the Qiagen Allprep DNA/RNA Mini Kit. miRNA isolation of tissue samples was performed using the mirVanaTM miRNA Isolation Kit (Thermo Fisher Scientific) according to manufacturer's instructions.

## cDNA synthesis and qPCR

cDNA synthesis was conducted using SuperScript II Reverse Transcriptase (Thermo Fisher Scientific) using 1 $\mu$g of total RNA according to standard protocols. Real-time qPCR was conducted with SYBR Select Master Mix (Thermo Fisher Scientific) with primers listed in Table S19. Murine and human *GAPDH* were used as housekeeping genes for normalization. For microRNAs, expression was assessed using the TaqMan™ technology. cDNA was synthesized using the TaqMan™ Advanced miRNA cDNA Synthesis Kit (Thermo Fisher Scientific). Expression was assessed using the TaqMan™ Advanced miRNA assays hsa-miR-29a-3p and hsa-miR-29b-3p for microRNA29a and microRNA29b, respectively. Expression was normalized to microRNA16 using the hsa-miR-16-5p assay (all Thermo Fisher Scientific).

## 3-Prime RNA sequencing

Library preparation for bulk-sequencing of poly(A)-RNA was done as described previously.[115] Briefly, barcoded cDNA of each sample was generated with a Maxima RT polymerase (Thermo Fisher) using oligo-dT primer containing barcodes, unique molecular

identifiers (UMIs) and an adaptor. Ends of the cDNAs were extended by a template switch oligo (TSO) and full-length cDNA was amplified with primers binding to the TSO-site and the adaptor. NEB UltraII FS kit was used to fragment cDNA. After end repair and A-tailing a TruSeq adapter was ligated and 3'-end-fragments were finally amplified using primers with Illumina P5 and P7 overhangs. In comparison to Parekh et al.,[115] the P5 and P7 sites were exchanged to allow sequencing of the cDNA in read1 and barcodes and UMIs in read2 to achieve a better cluster recognition. The library was sequenced on a NextSeq 500 (Illumina) with 63 cycles for the cDNA in read1 and 16 cycles for the barcodes and UMIs in read2.

### RNA-seq data analysis

Gencode gene annotations M25 and the mouse reference genome GRCm38 were derived from the Gencode homepage (EMBL-EBI). Data was processed using the published Drop-Seq pipeline (v1.12) to generate sample- and gene-wise UMI tables.[116] The resulting UMI filtered count matrix was imported into R v4.0.1. Lowly expressed genes were filtered so that 80% of samples have at least three read counts per gene. The data was normalized to sequencing depth (within sample normalization) and variance stabilized (between sample normalization). This was done via the rlog transformation implemented in the DESeq2 package and dispersion of the data was estimated with an intercept only model using DESeq2 v1.28.1.[117] Details on statistical analysis are described in the chapter 'Quantification and Statistical Analysis'.

### GSEA

For gene set enrichment analysis GSEA v4.0.3[85] and the hallmark gene sets (h.all.v7.2.symbols.gmt) were used. Hematopoietic gene signatures were obtained from Laurenti et al. (http://www.jdstemcellresearch.ca/node/32) and Novershtern et al.[118,119] A pathway was considered to be significantly associated with an experimental condition if the FWER was below 0.05. All statistical values can be found in Table S15. Details on statistical analysis are described in the chapter 'Quantification and Statistical Analysis'.

### Analyses of GWAS data

The GWAS catalog was downloaded from https://www.ebi.ac.uk/gwas/ (EMBL-EBI).[80] All 'associations' with available ontology annotations, GWAS Catalog study accession numbers and genotyping technology were used (v1.0.2). Disease traits were filtered for "cancer"/"tumor"/"neoplasm" and/or "leukemia"/"lymphoma" to get cancer- and hematologic malignancies-associated variants, respectively. All studies resulting from this filtering were used. We assessed whether reported genes in the GWAS catalog were over-represented among CIS-target genes (Table S11).

We additionally performed lift-over of mouse CISs (5 k size parameter) coordinates to the human genome (hg38) using the UCSC liftover tool and used the syntenic human regions to analyze their overlap with cancer-associated GWAS variants. Details on statistical analysis are described in the chapter 'Quantification and Statistical Analysis'.

## QUANTIFICATION AND STATISTICAL ANALYSIS

### General statistical analyses

All statistical analyses were performed using R v4.0.1. Methods used for statistical hypothesis testing and exact n numbers are directly stated in the figure legends. In general, the significance level was set to 0.05. Boxplots were generated using the default ggplot2 geom_boxplot settings (middle, median; lower hinge, 25% quantile; upper hinge, 75% quantile; upper/lower whisker, largest/smallest observation less/greater than or equal to upper/lower hinge ±1.5 * IQR).

### CIS analysis

For CIS analysis, CIMPL assigns a p value to each CIS (listed in Table S2) and controls the errors at an average of 5%.

### RNA-seq data analysis

Principal Component Analysis (PCA) was conducted with the 10 percent top variable genes in the rlog transformed dataset. The cola R package was used to compare different clustering methods.[86] The cola package provides a general framework for subgroup classification by consensus partitioning. The rlog transformed expression matrix was used as an input and cola was run with default parameters. The results show that 2 or 4 clusters were predicted as best choice (statistical details in Table S14, Figure S13A). After careful review of the biology behind the clusters, the combination of CV (coefficient of variance) as top value method and mclust as clustering method was chosen for downstream analyses. This approach predicted 4 as best k parameter and resulting cluster assignments are shown in the PCA embedding (Figure S13). Samples showing ambiguous clustering were not used in downstream analyses (Table S1). Detailed subtype analyses (Figures 6C–6J) were only performed on this subset of samples (ETP-like n = 14, classical n = 8, Mef2c-driven n = 7). Cluster assignments were then used as explanatory variable during model fitting with DESeq2. The Wald test was used for determining differentially regulated genes between all pairwise clusters. Shrunken log2 fold changes were calculated afterward. A gene was determined to be significantly regulated if the p value was below 0.05. Rlog transformation of the data was performed for visualization and further downstream analysis.

### GSEA

GSEA v4.0.3[85] was used to perform gene set enrichment analysis in the preranked mode using the apeglm shrunken log2 fold changes as ranking metric. apeglm shrinkage is a process to correct foldchanges that are overestimated due to low expression of genes or highly variable genes.[120] A pathway was considered to be significantly associated with an experimental condition if the FWER was below 0.05. All statistical values can be found in Table S15.

### Analyses of GWAS data

The GWAS catalog was downloaded from https://www.ebi.ac.uk/gwas/ (EMBL-EBI).[80] We assessed whether reported genes in the GWAS catalog were over-represented among CIS-target genes. For enrichment calculation, the number of protein-coding genes in the genome was used as a control (19,370; Gencode). $\chi^2$ test was used to calculate enrichment p values (Table S11).

We additionally performed lift-over of mouse CISs (5 k size parameter) coordinates to the human genome (hg38) using the UCSC liftover tool and used the syntenic human regions to analyze their overlap with cancer-associated GWAS variants. Variants (n = 8,677) were pruned for linkage disequilibrium using the SNPclip tool (https://analysistools.cancer.gov/LDlink/?tab=snpclip) from LDlink[60] with a threshold of $R^2$ = 0.8 and MAF = 0.01. The thinned list of variants (n = 4,625) was used for overlap with CIS regions. $\chi^2$ test was used to calculate enrichment p values considering the sum of all human CIS regions (size in basepairs) in comparison to the size of the complete genome (Table S12).

Cell Genomics, Volume *3*

## Supplemental information

# *In vivo* interrogation of regulatory genomes reveals extensive quasi-insufficiency in cancer evolution

**Anja Fischer, Robert Lersch, Niklas de Andrade Krätzig, Alexander Strong, Mathias J. Friedrich, Julia Weber, Thomas Engleitner, Rupert Öllinger, Hsi-Yu Yen, Ursula Kohlhofer, Irene Gonzalez-Menendez, David Sailer, Liz Kogan, Mari Lahnalampi, Saara Laukkanen, Thorsten Kaltenbacher, Christine Klement, Majdaddin Rezaei, Tim Ammon, Juan J. Montero, Günter Schneider, Julia Mayerle, Mathias Heikenwälder, Marc Schmidt-Supprian, Leticia Quintanilla-Martinez, Katja Steiger, Pentao Liu, Juan Cadiñanos, George S. Vassiliou, Dieter Saur, Olli Lohi, Merja Heinäniemi, Nathalie Conte, Allan Bradley, Lena Rad, and Roland Rad**

Figure S1

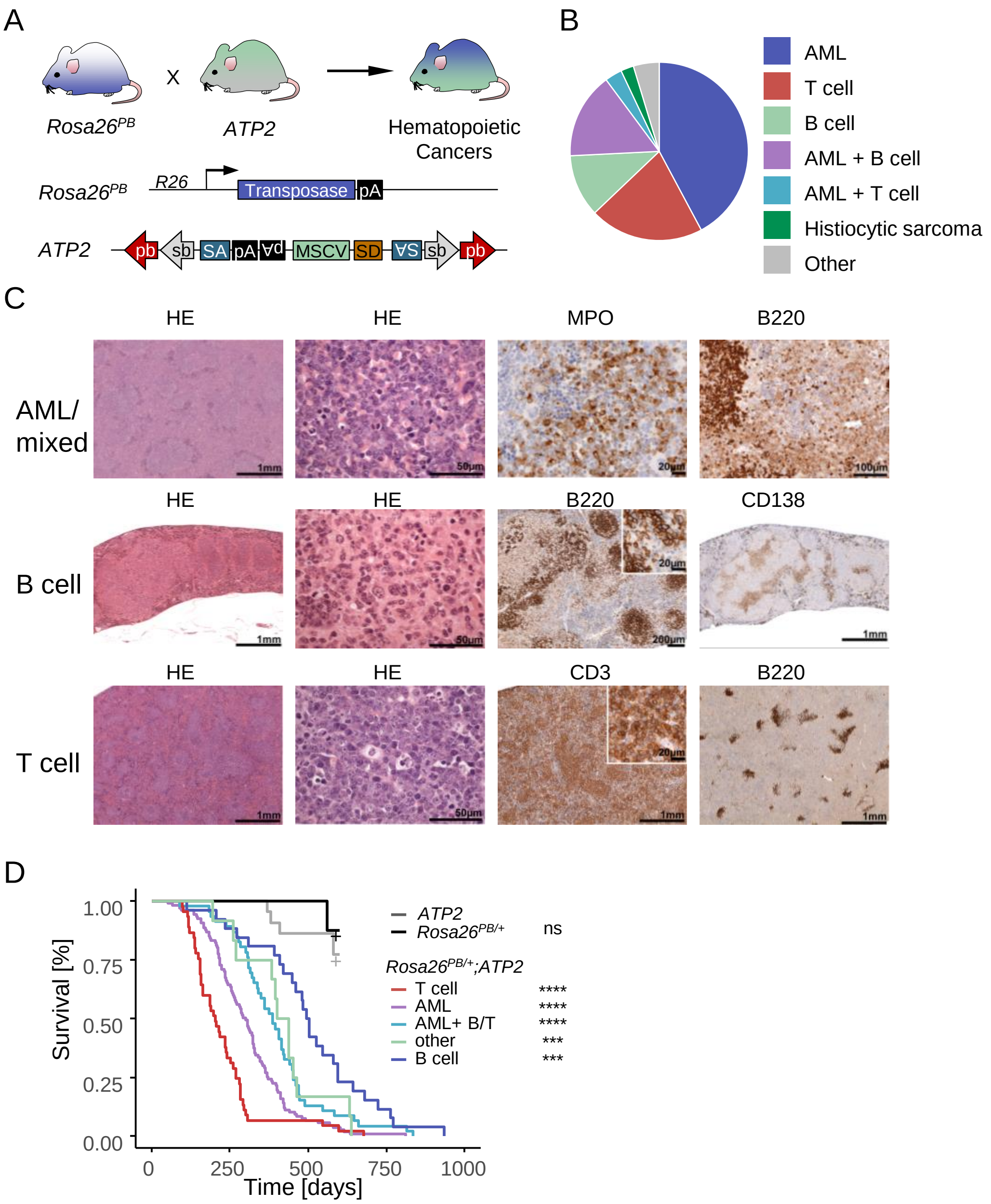


**Supplementary Figure 1. Histopathological characterization of the Rosa26PB;ATP2 screening cohort. A,** Mating scheme for *PiggyBac* screening using ATP2 mouse lines. In *Rosa26PB;ATP2* double positive mice transposon mobilization is active and mice develop hematopoietic cancers. Structure of Rosa26PB allele and ATP2 transposons used to generate ATP2 transgenic mice. **B,** Overview of histopathological diagnoses of hematopoietic malignancies in *Rosa26PB;ATP2* mice. Spectrum of 256 hematopoietic tumors diagnosed in the ATP2 screening cohort. Samples classified as 'other' represent samples where (i) no diagnosis was possible due to tissue lysis, (ii) the diagnosis of a benign immunological phenomenon such as spleen hyperplasia or (iii) the diagnosis of a lymphoblastic proliferation not further characterized. **C,** Representative images of tumors from all major subgroups. The first row shows an acute leukemia (AML) case with MPO expression. Additionally, the shown AML case expresses the B cell marker B220. The second row represents a diffuse large B cell lymphoma (DLBCL) characterized by B220 expression. The additional expression of CD138 led to the classification of DLBCL with plasmacytoid differentiation. The third row shows a T cell (lymphoblastic) lymphoma characterized by strong CD3 expression and the absence of B220 expression. Magnifications: first column: 25x; second: 630x; third: MPO 400x, B220 50x (insert 400x), CD3 25x (insert 400x); fourth: B220 200x, CD138 25x, B220 25x. **D,** Kaplan-Meier plot showing survival of *Rosa26PB/+;ATP2* and single-allele control mice. Survival of Rosa26PB;ATP2 mice with different types of hematopoietic tumors (AML n = 107, AML + B/T cell n = 46, B cell n = 26, T cell n = 45, other n = 12). All comparisons between the single-allele ATP2 control mice and the tumor cohorts using the Log-Rank test are shown. Benjamini-Hochberg (FDR) was used to adjust for multiple testing. *P < 0.05, **P < 0.01, ***P < 0.001, ****P < 0.0001. Related to Table S1.

Figure S2

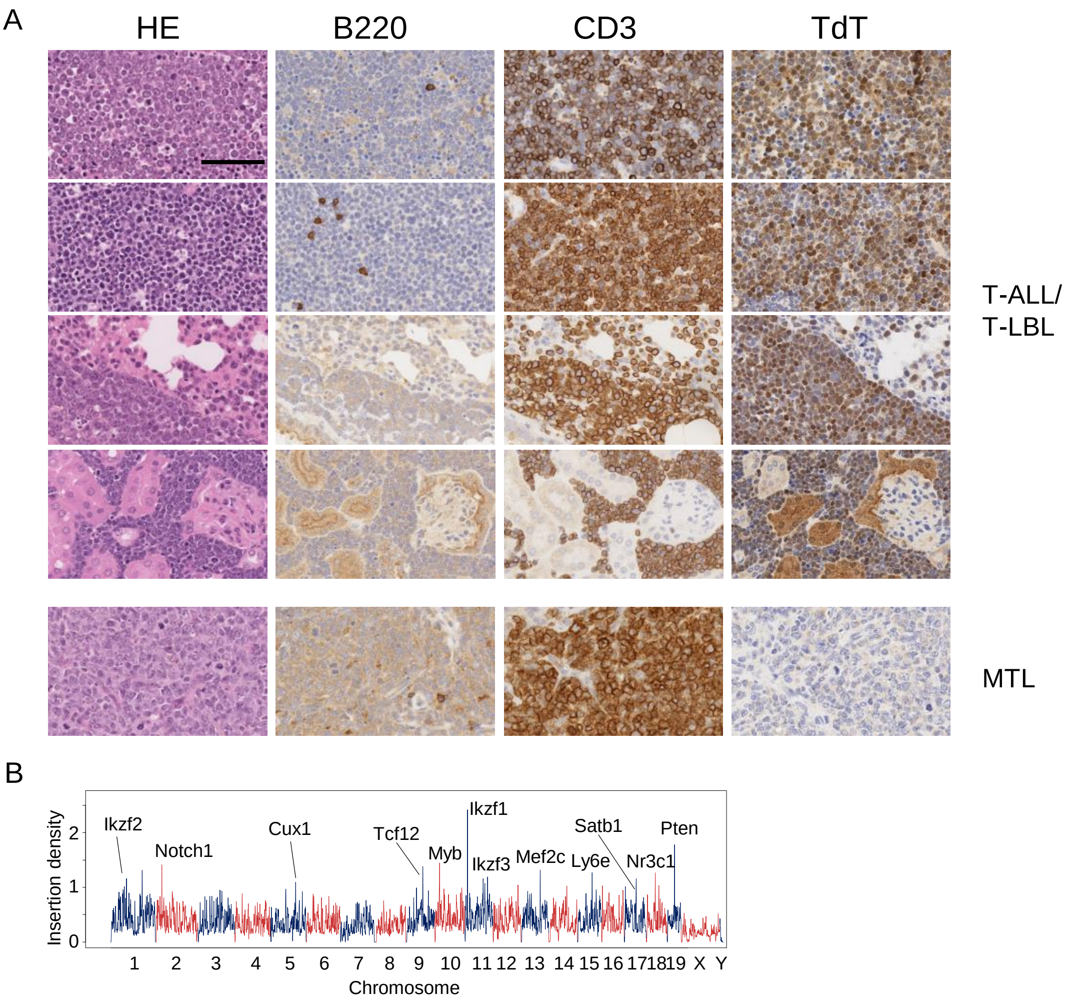


**Supplementary Figure 2. Histopathological classification of Rosa26$^{PB/+}$;ATP2 transposon mice with T cell malignancies. A,** Immunohistochemical characterization of mouse T cell leukemias/lymphomas. Microscopic images of five representative cases, 4 classified as T-ALL/T-LBL and one as mature T cell lymphoma (MTL). T-ALL/T-LBL cases were characterized by medium sized cells, round to ovoid nuclei and a scant cytoplasm. The nuclei had fine or dispersed (salt and pepper) chromatin and usually one central nucleolus. MTLs were negative for Tdt and showed a heterogeneous morphology ranging from cases with small cell size and inconspicuous nuclei to ones with medium cell size with irregular nuclei. Tumors are negative for B220/CD45R (B cell marker) and show strong expression of CD3 (T cell marker). CD3 expression was found on the membrane, but also in the cytoplasm. Cytoplasmic expression of Tdt (early T cell marker) was used to differentiate between T-ALL/T-LBL and mature T cell lymphomas (MTL). T-ALLs show infiltration in lung (third panel) and kidney (fourth panel). Tumors were classified according to the Bethesda proposals for classification of lymphoid neoplasms in mice (Morse et al., 2002). Scale bar, 50 µm. **B,** Genome-wide representation of transposon insertion density in T-ALLs (pooled data from 51 mice). Selected common insertion site target genes are indicated. Colors are used to distinguish chromosomes. Related to Table S1.

Figure S3

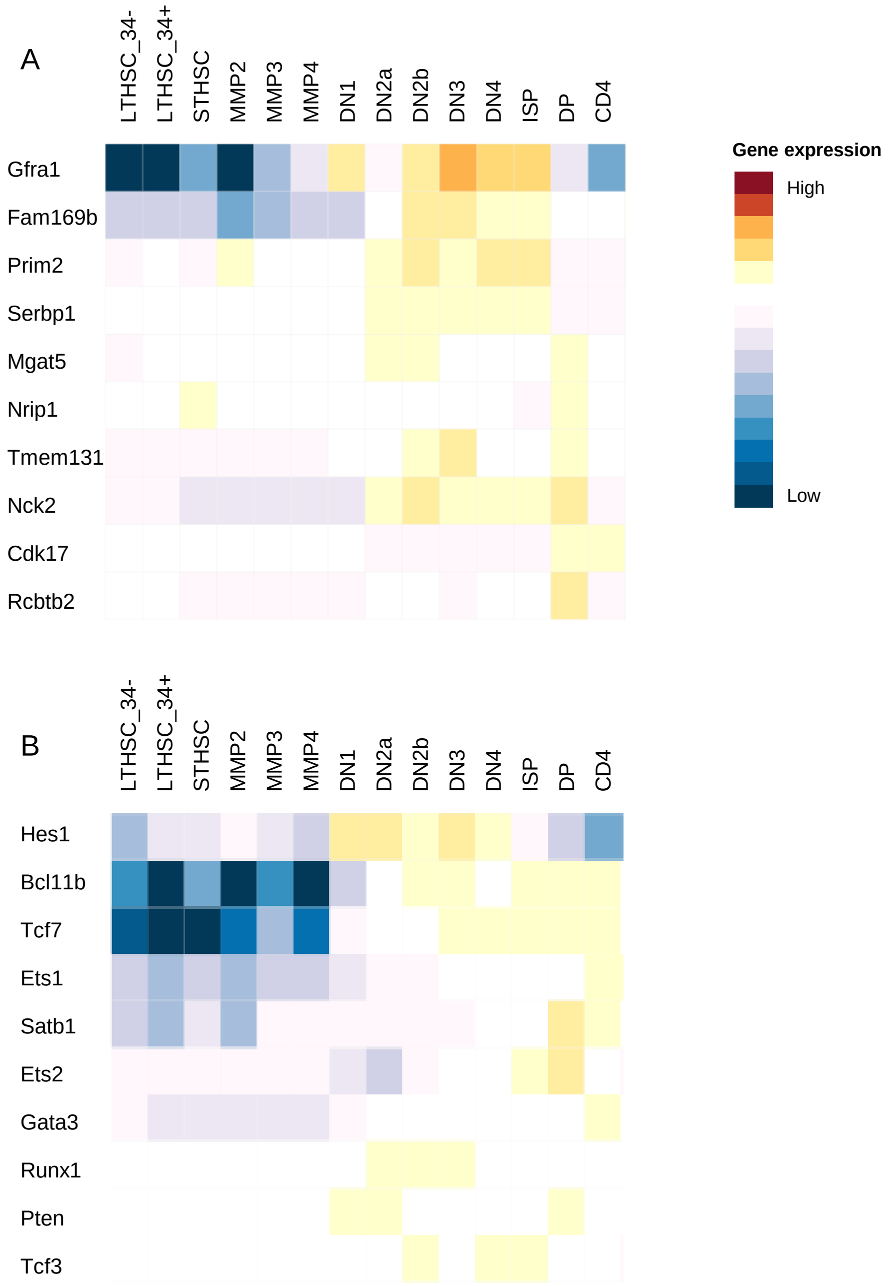


**Supplementary Figure 3. Expression of candidate genes in T cell development. A,** Heatmap showing expression of 10 candidate CIS genes with differential expression during murine T cell development. An increased expression level was observed in T cell precursor stages (*Prim2, Serbp1, Nck2*) or in later stages (*Nrip1, Cdk17, Rcbtb2*). **B,** Heatmap showing expression of known T cell developmental genes showing an increased expression level in T cell precursor stages (*Tcf7, Runx1*) or in later stages (*Satb1, Gata3, Ets2*). Mean-normalized heatmaps were created using the immunological genome project website (http://rstats.immgen.org/MyGeneSet_New/index.html). Related to Figure 1, Table S2/3.

Figure S4

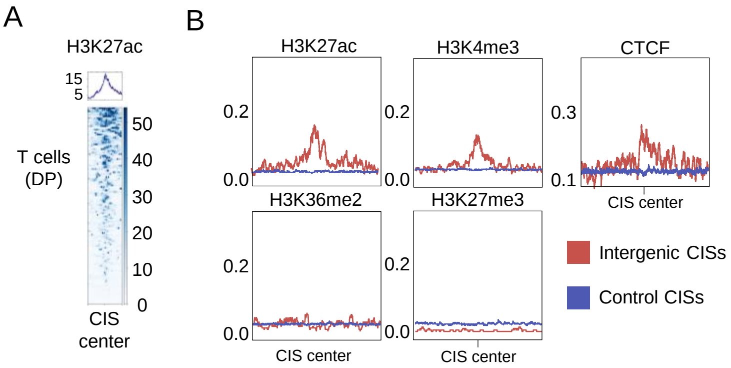


**Supplementary Figure 4: Characteristics of intergenic insertions. A)**. Profile heatmap plot showing overlap of non-coding CIS regions with H3K27ac peaks in T cells (DP stage). **B)** Footprint plots of intergenic CISs. Enrichment of chromatin features was assessed in intergenic CISs (n = 227) from all T-ALL samples. Input data of thymus tissue (CTCF) and the EL4 cell line was used. The background line represents a set of control CISs with similar characteristics (selection of control CISs is in detail described in the Methods part). Related to Figure 1.

Figure S5

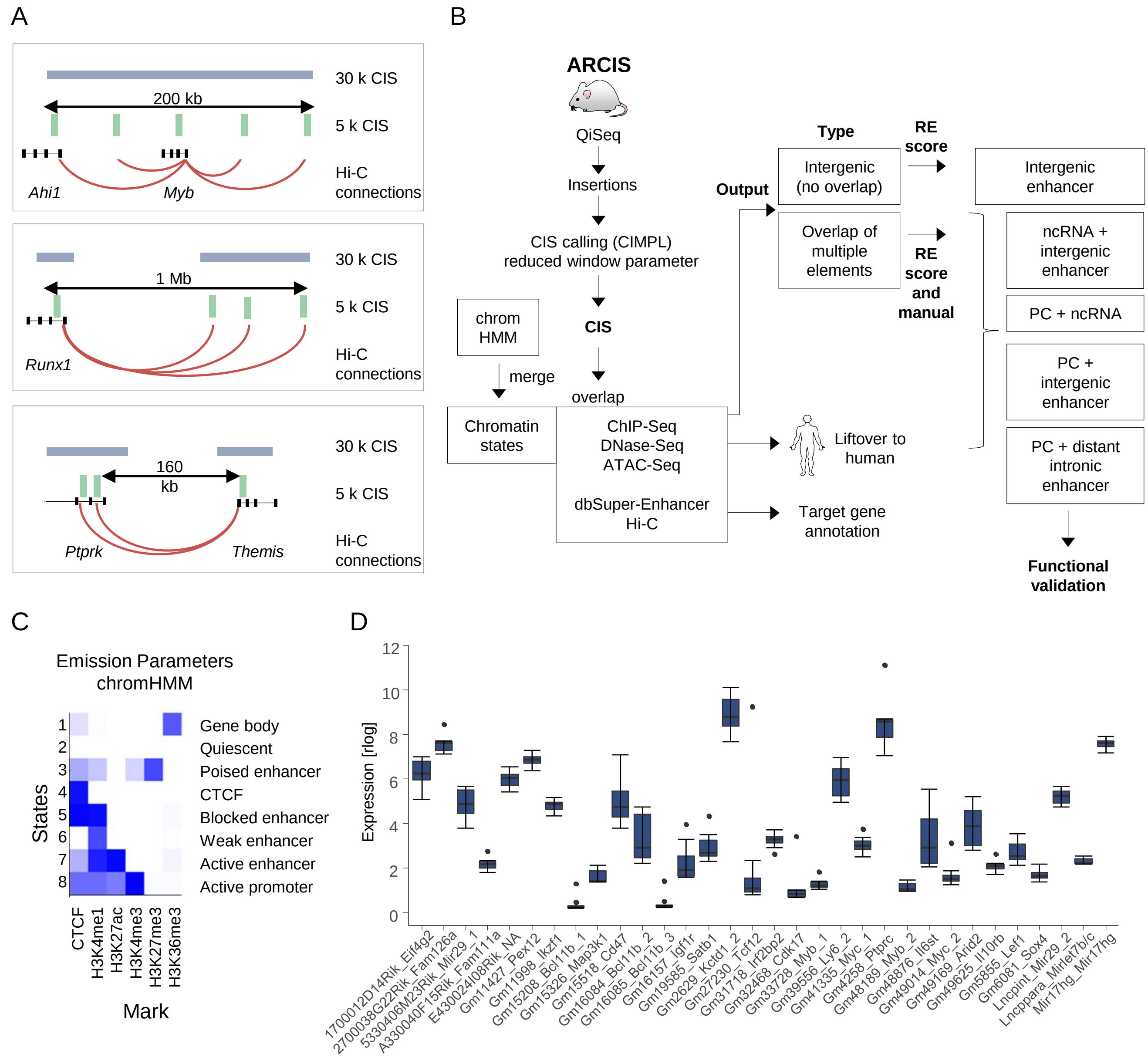


**Supplementary Figure 5. The ARCIS annotation framework. A,** Influence of size parameter change on common insertion sites (CIS). The effect of changing the scale parameter on CIS size using CIMPL. Exemplary loci of *Myb, Runx1* and *Themis* are shown. Blue bars represent CIS regions identified with a scale parameter of 30 k, green bars show CIS identified with a 5 k scale parameter. Relevant Hi-C connections from Hu et al. are schematically shown in red (GSE79422). To identify putative regulatory elements the desired (biologically relevant) scale needs to be adjusted by choosing a smaller window size. **B,** Schematic representation of the ARCIS framework to annotate putative regulatory elements in CIS regions. Intergenic CISs with a high "regulatory score" can directly be defined as intergenic enhancers, whilst CIS overlapping with multiple elements need further manual annotation. **C,** Emission parameters of the chromHMM model used as ARCIS input. ChromHMM was performed on thymus specific ChIP-Seq datasets (ENCODE). The 8 output states were defined as shown based on a combination of ChIP-Seq signals. **D,** rlog transformed expression values of non-coding transcripts identified by ARCIS in different stages of T cell development (HSC-DP, GSE79422). The CIS non-coding transcript and the PC gene located in close proximity are annotated. ARCIS identified 54 non-coding RNAs, 44 were annotated in the dataset and 32 were found expressed (73%). In total, the dataset includes 12,170 lncRNAs and miRNAs, of which only 1,925 are expressed (16%) with a median rlog expression level of 2,8. Related to Figure 2, Table S4/5.

Figure S6

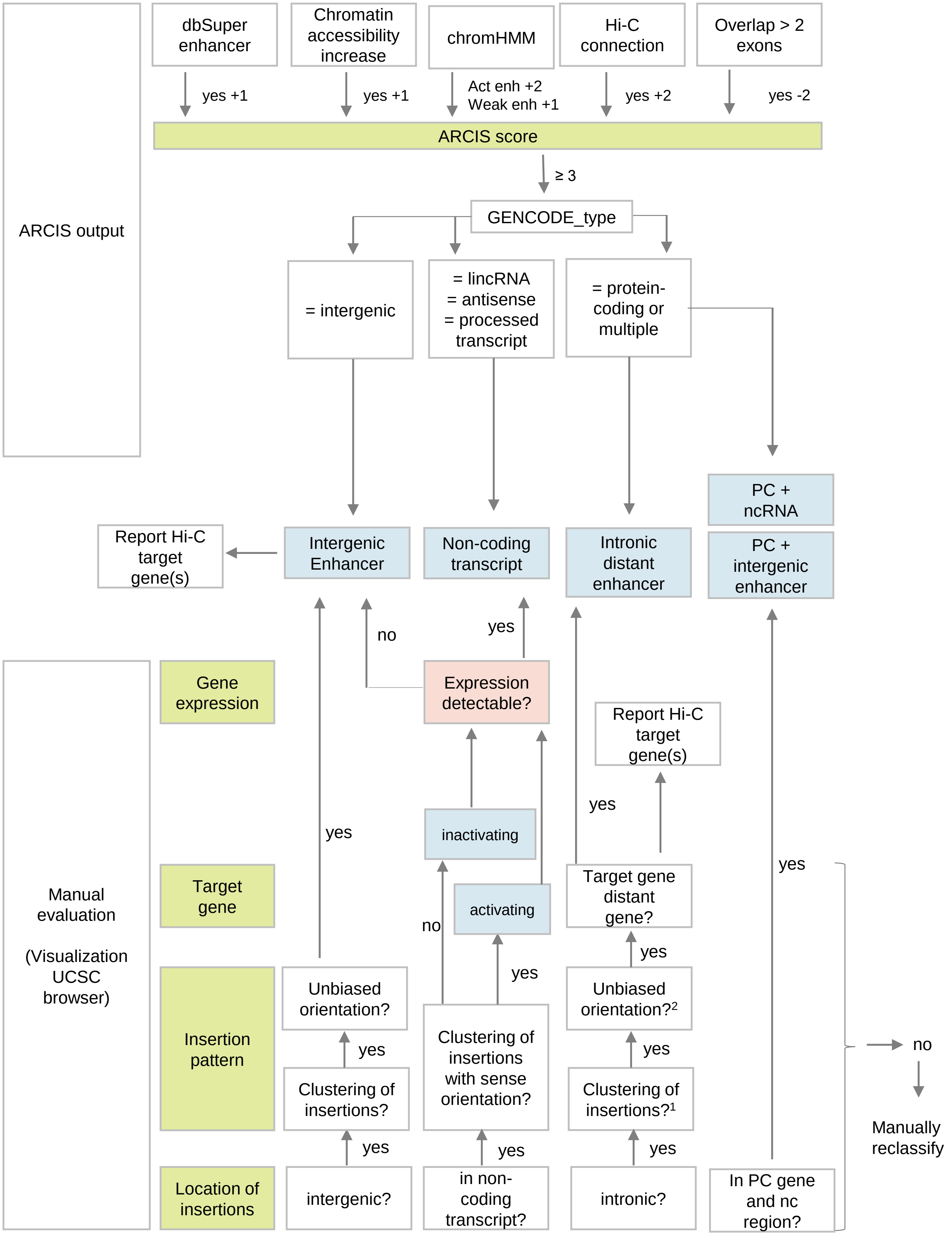


1 no: inactivated PC gene
2 no: activated PC gene

**Supplementary Figure 6. Identification of putative regulatory regions based on ARCIS and manual evaluation.** ARCIS calculates a score for each CIS region. The score is based on selected elements: overlap with an annotated super-enhancer (dbSuper) or increasing chromatin accessibility in T cell development (from Hu et al) was scored with +1, a weak and active enhancer (chromHMM) was scored with +1 and +2, respectively, any reported Hi-C connection was scored with +2. To avoid that inactivated PC genes are reported as intronic enhancer, the overlap with more than two exons was penalized with -2. The sum represents the final ARCIS score. If the ARCIS score is greater than or equal to 3, the CIS is putatively harboring a regulatory region. CIS can be ranked based on the score. To report the type of the element, the GENCODE M24 annotation type is used. According to the type, the CIS gets annotated as intergenic enhancer, as non-coding transcript, which is potentially overlapping with an enhancer or as protein-coding transcript, which might additionally harbor an intragenic or a close intergenic RE. The category 'non-coding' is defined by the overlap with an annotated nPC transcript, but does not exclude the presence of an additional enhancer element. In contrast, the annotation as enhancer element cannot exclude the presence of so far not annotated nPC transcripts. Assessing the expression of identified nPC-transcripts as shown in Supplementary Figure 5d represents further implication for the nPC transcript to play a functional role (also explained below).
The manual evaluation was based on the visualization of different datasets (Supplementary Table 5) in the UCSC genome browser. Here, we inspected the exact position of the insertions, their orientation and clustering within the CIS. In a first step we assessed whether or not the majority of the insertions overlaps with an intergenic/intronic region, non-coding transcript or with multiple types. For intergenic enhancers, we next investigated the clustering of the insertions, a characteristic for insertion peaks in REs. To exclude that this insertion peak is activating a transcript, we assessed the orientation of the insertions. If the orientation is unbiased, it is highly unlikely that these insertions activate a transcript. If any of these steps leads to a 'no' answer meaning that either insertions are not clustered or the orientation is biased, the CIS needs to be manually reclassified to the better matching category. If all steps were answered with 'yes', the target gene of the RE is reported based on the visible Hi-C connection. An optional step (red color) includes the investigation if the reported target gene is expressed in T cell development (based on RNAseq data from Hu et al.). A target gene expressed at any stage in T cell development, represents a highly interesting candidate. For intronic REs, the steps are comparable. If insertions are not clustered, the PC gene most likely gets inactivated by the transposon insertions, classifying this CIS as PC (marked with 1). If the insertions are clustered, but show strong orientation bias, the transposon might activate the PC gene (based on availability of a transcript with an ATG in exon 2 or higher). These CISs were classified as activating PC (marked with 2). If all steps were answered with 'yes', it needs to be assessed if the target gene is a distant gene (not connected to own promoter). If so, the target gene is reported. An optional step (red color) includes the investigation if the CIS gene (not the target gene) is expressed in T cell development. For genes not expressed, an inactivating function of the transposon insertions is highly unlikely, increasing the confidence to classify this CIS as intronic regulatory element. For non-coding transcripts, we differentiate into potentially activating or rather inactivating patterns based on insertion clustering and orientation. An optional step (red color) includes the investigation if the non-coding transcript is expressed in T cell development. Of note, nPC transcripts are expressed at low levels compared to PC genes. Identifying a transcript with detectable expression therefore increases the confidence that the nPC transcript is relevant for T cell biology. If insertions overlap with multiple transcripts (PC and nPC) or with a PC transcript and the intergenic area, these CISs were classified as 'PC + ncRNA' or 'PC + intergenic enhancer', respectively. Ambiguity in the ARCIS output often cannot be resolved by manual annotation. Here, functional validation is necessary.
PC, protein-coding; nPC, non-protein-coding; CIS, common insertion site.
Related to Figure 2, Table S7/8.

Figure S7

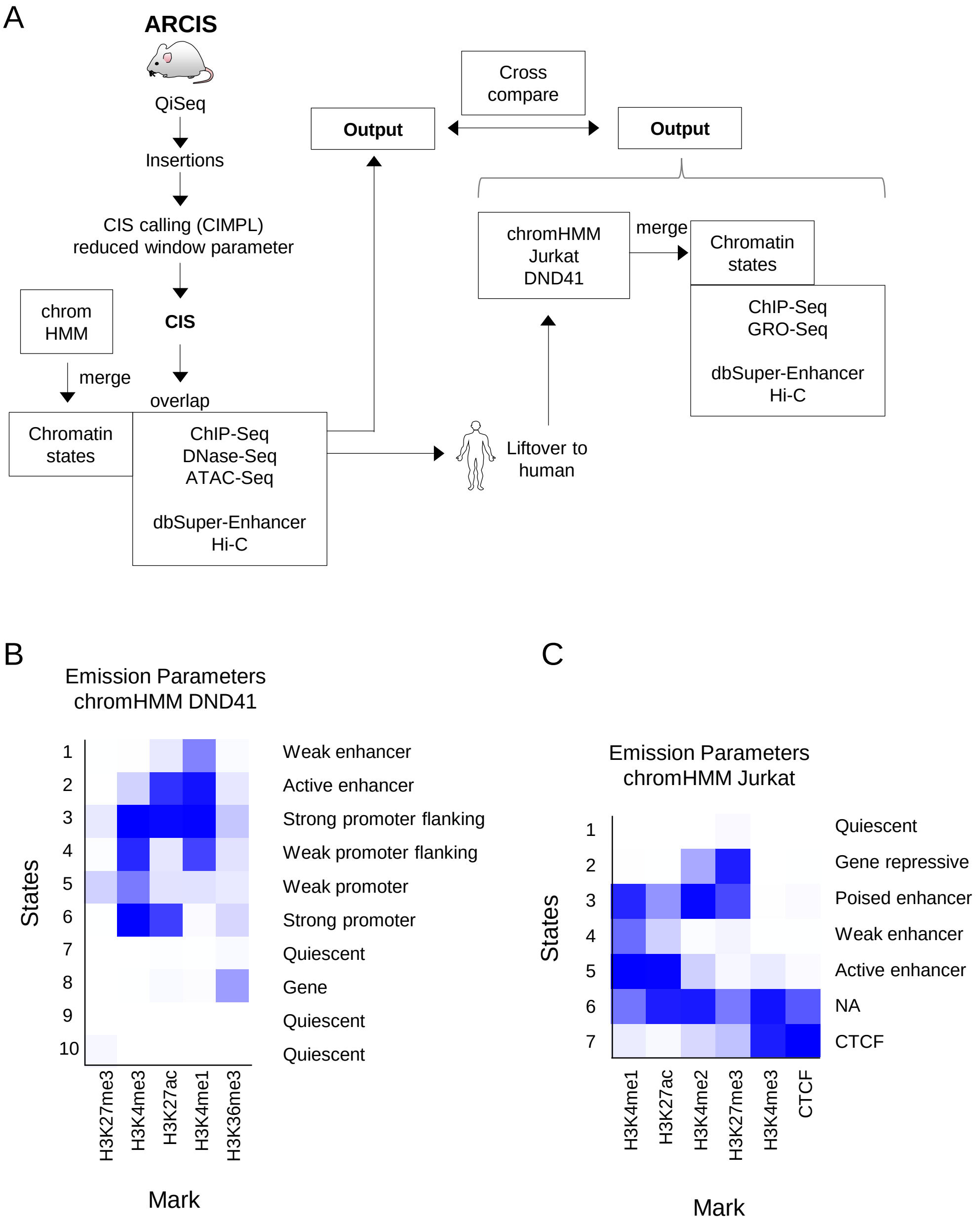


**Supplementary Figure 7. Human ARCIS**. **A,** Scheme showing workflow for the annotation of CISs after lift-over to the human genome. ChromHMM was performed for the T-ALL cell lines Jurkat and DND41 and publicly available ChIP-Seq, dbSuper-Enhancer and HiC data was used for annotation. Additionally, GRO-Seq data of the Jurkat and 2 T-ALL patients generated in this study was integrated. The output of the human ARCIS was cross-compared to the murine output (results shown in Figure 2d). **B,** ChromHMM Emission parameters and state annotation for the T-ALL cell line DND41. **C,** ChromHMM Emission parameters and state annotation for the T-ALL cell line Jurkat. Active and weak enhancer regions were used for CIS annotation. Related to Figure 2, Table S6/9.

Figure S8

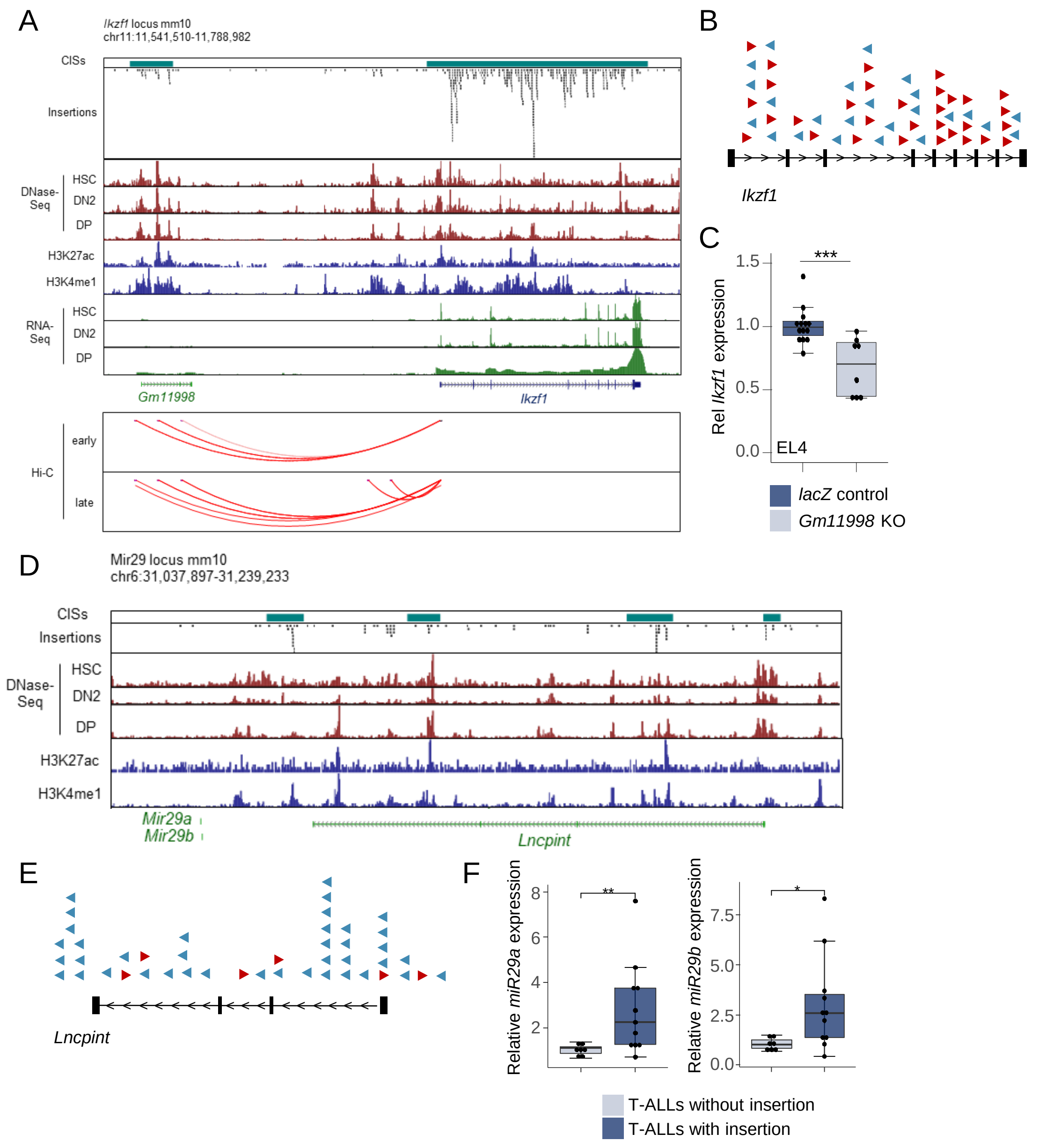


**Supplementary Figure 8. Functional validation of non-coding CISs**. **A,** Insertions and CISs in the murine *Ikzf1* gene locus. H3K27ac and H3K4me1 tracks from double-positive T cells, as well as DNase-Seq and Hi-C data from different stages of T cell evolution are shown below (early: HSC-DN2a, late: DN2b-DN3) (publicly available data as listed in Supplementary Table 5). An intergenic CIS region overlapping *Gm11998 also* overlaps with active chromatin and shows a Hi-C link to the *Ikzf1* promoter. **B,** Insertion pattern in *Gm11998*. Sense- and antisense-oriented insertions are depicted in different colors. **C,** *Ikzf1* expression in clones with CRISPR/Cas9-based knockout of the *Gm11998* lincRNA in the T cell lymphoblastic EL4 cell line (KO n = 8, 0/8 homozygous, ctrl n = 14). A region of ~18 kb was knocked out and electroporated cells were single cell sorted. Each dot represents relative *Ikzf1* gene expression in a single cell-derived clone normalized to *Gapdh* expression. **D,** Murine *Lncpint/miR29* gene locus showing several CISs with insertion orientations in sense direction (promoter directed to miR29a/b). **E,** Insertions pattern in the *Lncpint/miR29* locus. Sense- and antisense-oriented insertions are depicted in different colors. **D+E)** We observed four CISs in a 150 kb region upstream of the microRNA mir29a/b, of which three are overlapping with the lncRNA Lncpint. In all CISs, transposon insertions were sense-oriented, reflecting strong selective pressure for effects mediated by the transposon's unidirectional MSCV promoter. The pattern and location of all CISs is compatible with effects supporting over-expression of mir29a, whilst overexpression of full-length Lncpint is only supported by one of the four CISs. We therefore hypothesized that miR29a/b is the target of these CISs. **F,** By performing a microRNA specific qPCR, we indeed found increased mir29a/b expression in samples with related *PiggyBac* insertions as compared to samples without insertions. Whilst such pronounced effects can be readily detected even in bulk tissue, we noted that this type of activating CIS driven by the transposon's strong MSCV promoter is rare in the non-protein coding genome. *miR29a/b* expression in clones with and without sense-directed insertion in the *Lncpint* locus (with insertion n = 11, without insertion n = 7). Specific Taqman assays were used for quantification by qPCR. Expression was normalized to miR16. *P < 0.05, **P < 0.01, ***P < 0.001, Wilcoxon test. CIS, common insertion site; HSC, hematopoietic stem cells; DN2, double negative stage 2; DP, double positive stage; Rel, relative. Related to Figure 3.

Figure S9

A

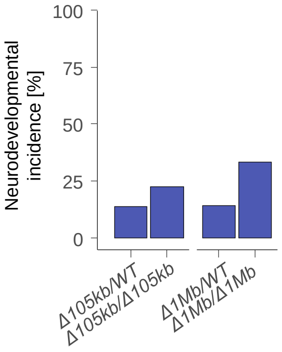


B

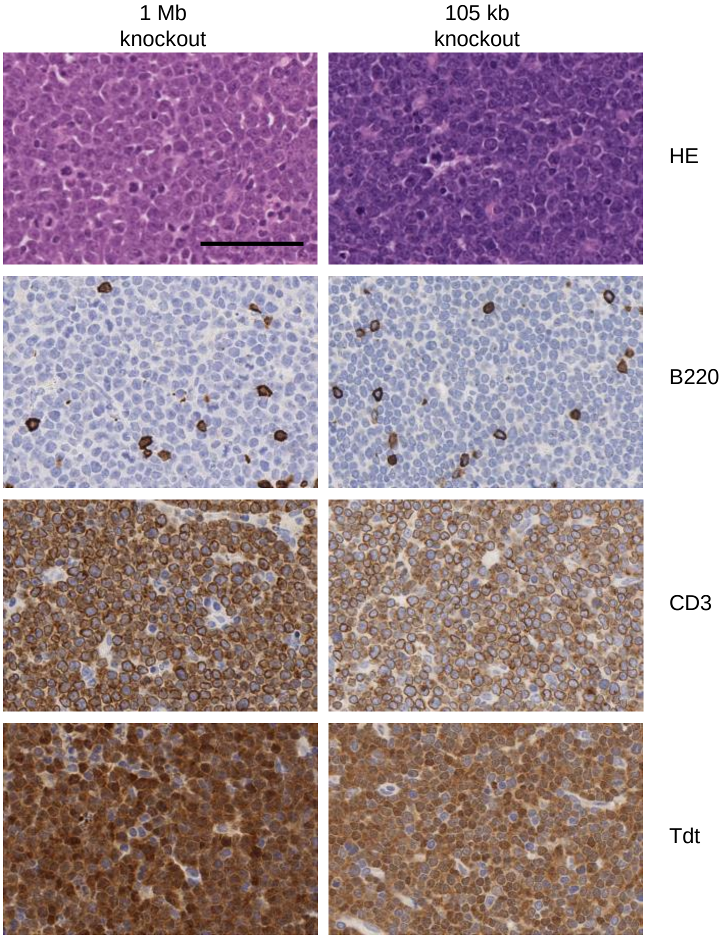


**Supplementary Figure 9. Survival, phenotype and histopathological classification of intergenic knockout mice**. **A,** Incidence of neurodevelopmental phenotype in all cohorts. Neurodevelopmental phenotypes include extremely small mice, mice with tremor-like symptoms or convulsions. **B,** Immunohistochemical characterization of mouse T cell leukemias/lymphomas. Microscopic images of a representative case of each cohort classified as T-ALL/T-LBL. Tumors are negative for B220 (B cell marker), show strong expression of CD3 (T cell marker) and Tdt (early T cell marker). Scale bar: 50 µm. Related to Figure 4, Table S12.

Figure S10

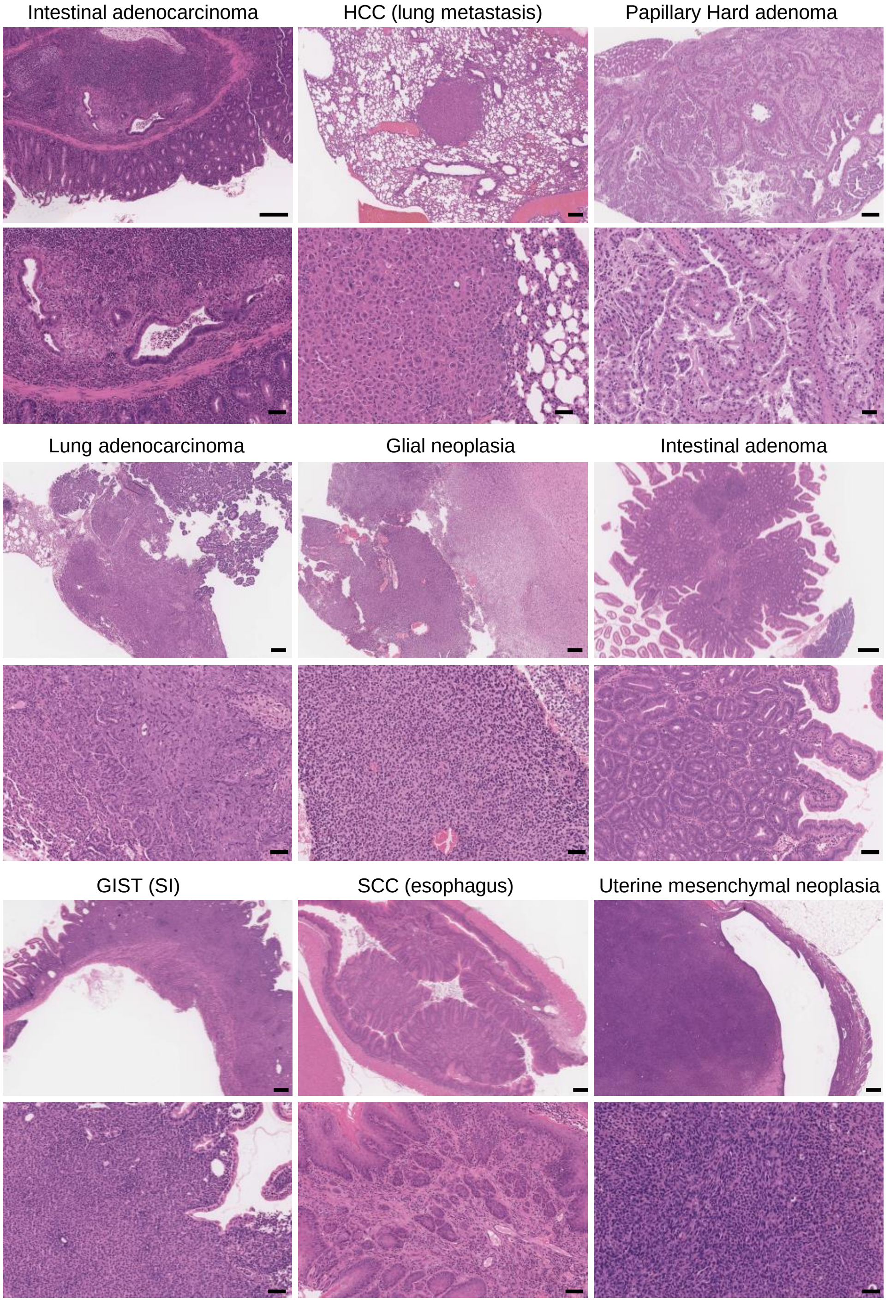


**Supplementary Figure 10. Histopathological classification of solid tumors developed in intergenic knockout mice.** Microscopic images show hematoxylin and eosin stainings of representative solid tumor cases from Δ105 kb and Δ1 Mb intergenic knockout mice. Only the lung adenocarcinoma was found in mice with the Δ1 Mb knockout, other solid tumors are from the Δ105 kb cohort. Two different magnifications are shown for each case. HCC, Hepatocellular carcinoma; Hard, Harderian gland; SCC, Squamous cell carcinoma; GIST, Gastrointestinal stromal tumor; SI, small intestine. Scale bars: upper panel 200 µm, lower panel 50 µm. Related to Figure 4, Table S12.

Figure S11

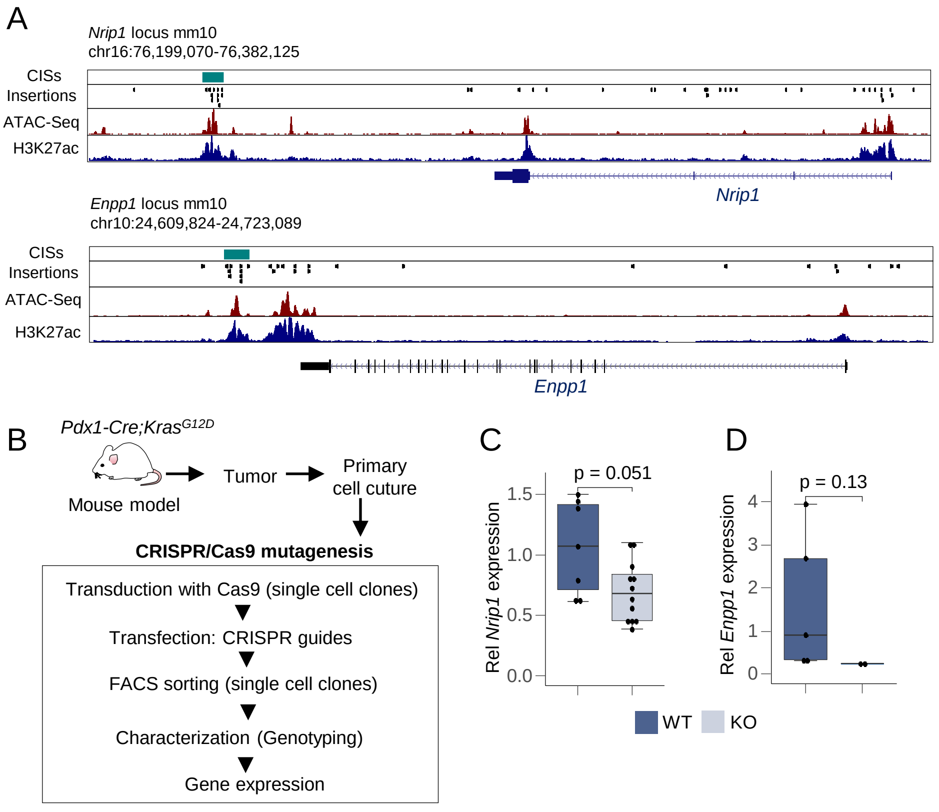


**Supplementary Figure 11. Functional validation of regulatory CISs in pancreatic cancer**. **A,** Insertions and CISs in the murine *Nrip1* (upper panel) and *Enpp1* (lower panel) gene locus. ATAC-Seq and H3K27ac tracks from a murine PDAC cell line are shown. **B,** Scheme showing functional validation workflow. Primary pancreatic ductal adenocarcinoma cell lines (PDAC) were isolated from mice as described in Müller et al., 2018. Stable Cas9 expressing cells were established by transducing a Cas9 expressing vector and single cell generation. Cas9-clones were further transfected with guide RNAs targeting the intergenic region of interest. As this single guide vector contains a GFP, cells were single-cell FACS sorted and expanded. Genetic knockout was characterized by PCR genotyping. Heterozygous and homozygous knockout clones were pooled (KO) and compared to wildtype (WT) clones in target gene expression analysis. **C,** *Nrip1* expression in clones with CRISPR/Cas9-based knockout of the intergenic region ~145 kb downstream of *Nrip1* in C2a-derived murine PDAC cell line (KO n = 12, 5/12 homozygous, WT n = 7). A region of ~4.5 kb was knocked out and transfected cells were single cell sorted. Each dot represents relative *Nrip1* gene expression in a single cell-derived clone normalized to *Gapdh* expression. p value is shown as a result of a t-test. **D,** *Enpp1* expression in clones with CRISPR/Cas9-based knockout of the intergenic region 80 kb downstream of *Enpp1* in C1-derived murine PDAC cell line (KO n = 2, 0/2 homozygous, WT n = 5). A region of ~3.5 kb was knocked out and transfected cells were single cell sorted. Each dot represents relative *Enpp1* gene expression in a single cell-derived clone normalized to *Gapdh* expression. p value is shown as a result of a t-test. Related to Figure 5, Table S13.

Figure S12

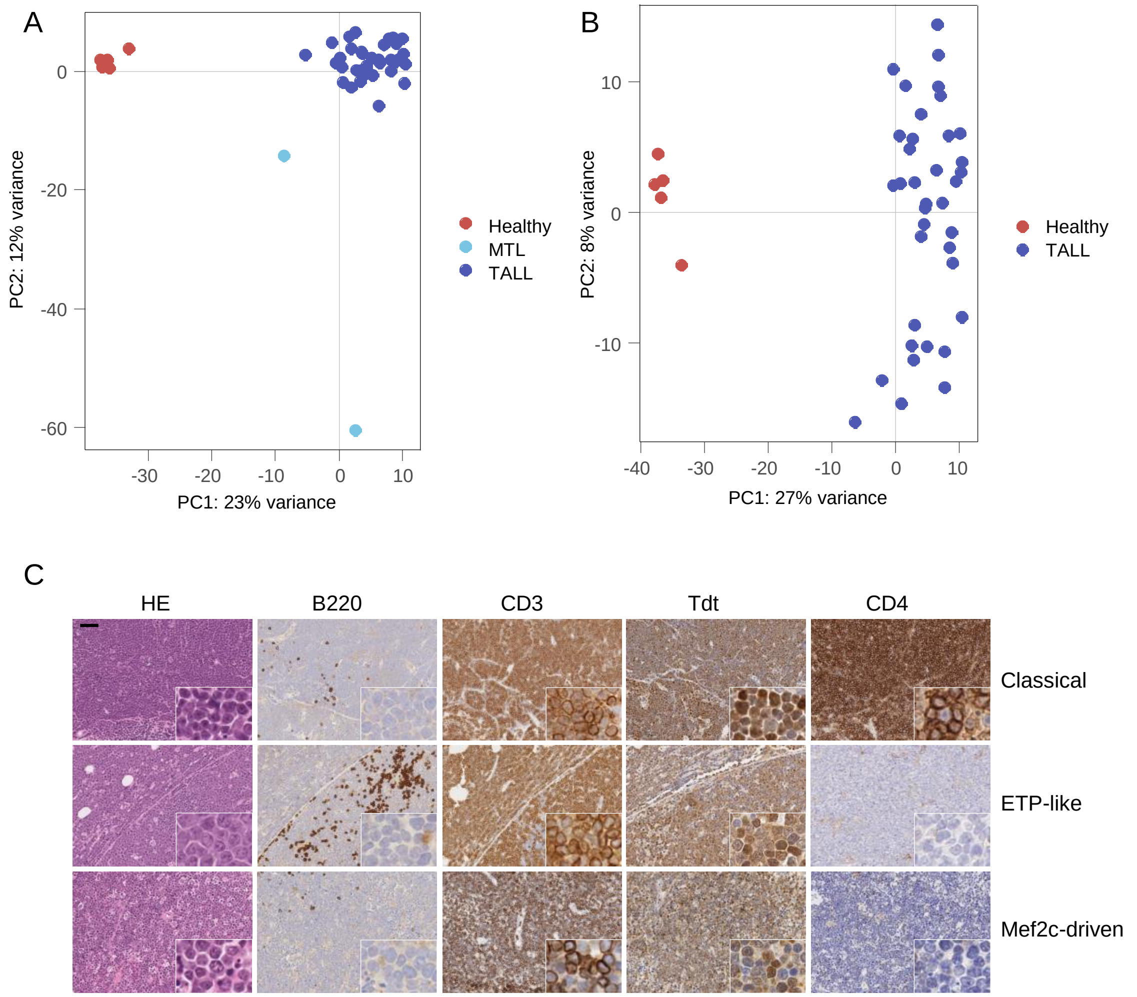


**Supplementary Figure 12. Transcriptome- and IHC-based subtyping of T cell leukemias/lymphomas developed in Rosa26$^{PB/+}$;ATP2 mice and healthy controls. A,** Mature T cell lymphomas (MTLs, light blue) cluster apart from T-ALL samples (dark blue) and healthy thymus (red). **B,** After removing MTL samples, T-ALL samples still cluster apart from healthy samples, but a variance in the PC2 can be detected. Only RNA isolated from thymus tissue was included in the analysis. Transcriptome-based subtyping confirms histological diagnoses, where MTLs did not show Tdt expression. **C,** Immunohistochemical characterization and sub-classification of T cell malignancies. CD4 positive T cell acute lymphoblastic leukemia/T cell lymphoblastic lymphoma (T-ALL/T-LBL) reflect the classical human T-ALL subtype. CD4 negative T-ALLs are immature pre- or pro-T ALLs. Scale bar, 50 µm. Related to Figure 6.

Figure S13

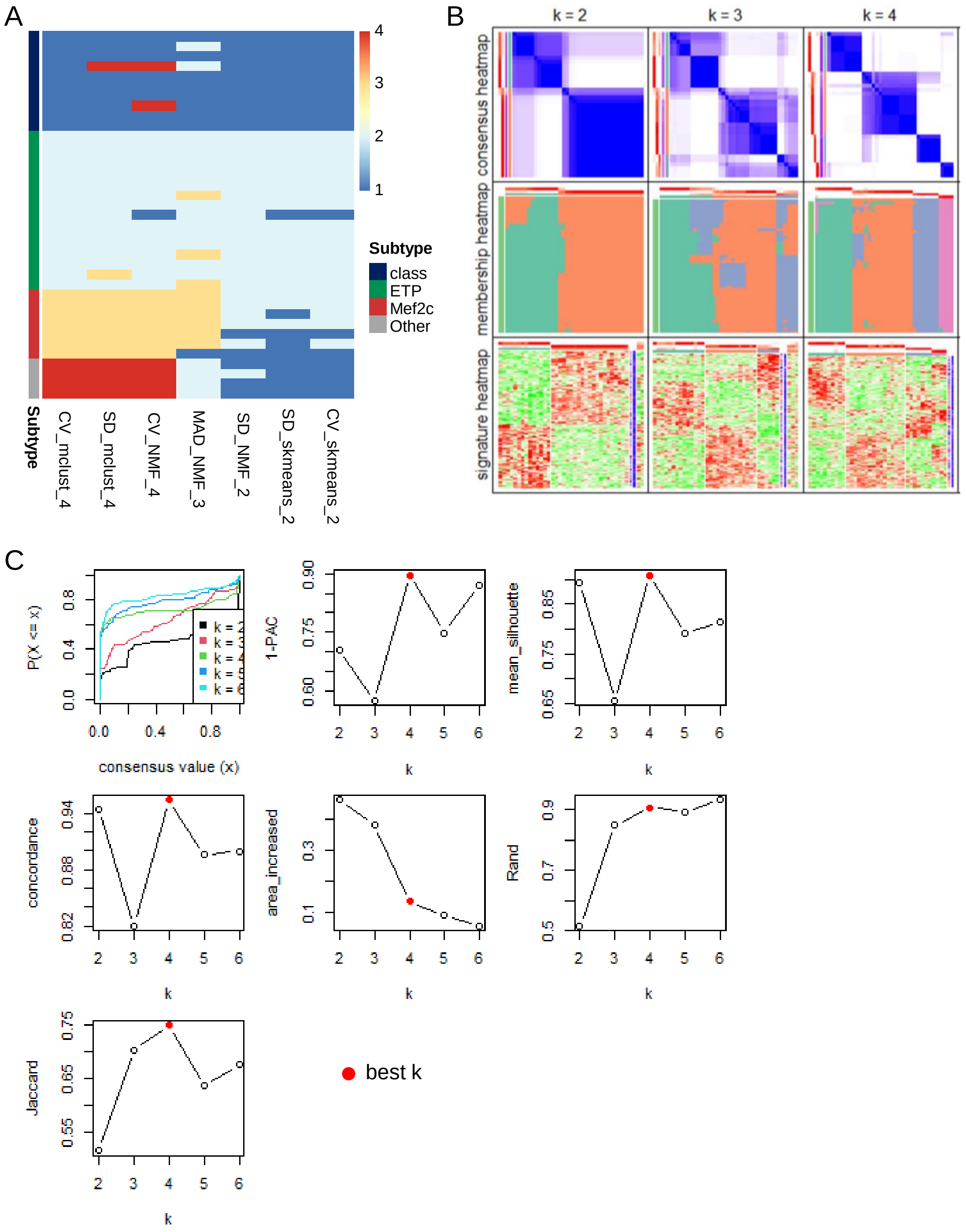


**Supplementary Figure 13. Clustering of RNA-Seq data**. The cola R package was used to compare multiple clustering approaches. **A,** Summary of subgroup assignments from highest ranked methods (full list available in Supplementary Table 14). The combination of top value method (SD, standard deviation; CV, coefficient of variance; MAD, median absolute deviation), clustering method (NMF, skmeans, mclust) and best k (2, 3, 4) is shown in the column names. Final subtype assignment is shown as column annotation. **B,** Consensus heatmap, membership heatmap and signature heatmap for the CV_mclust method for k=2, k=3 and k=4. **C,** Summary of statistical values for the CV_mclust method showing that k=4 is the best k. Related to Figure 6, Table S14.

Figure S14

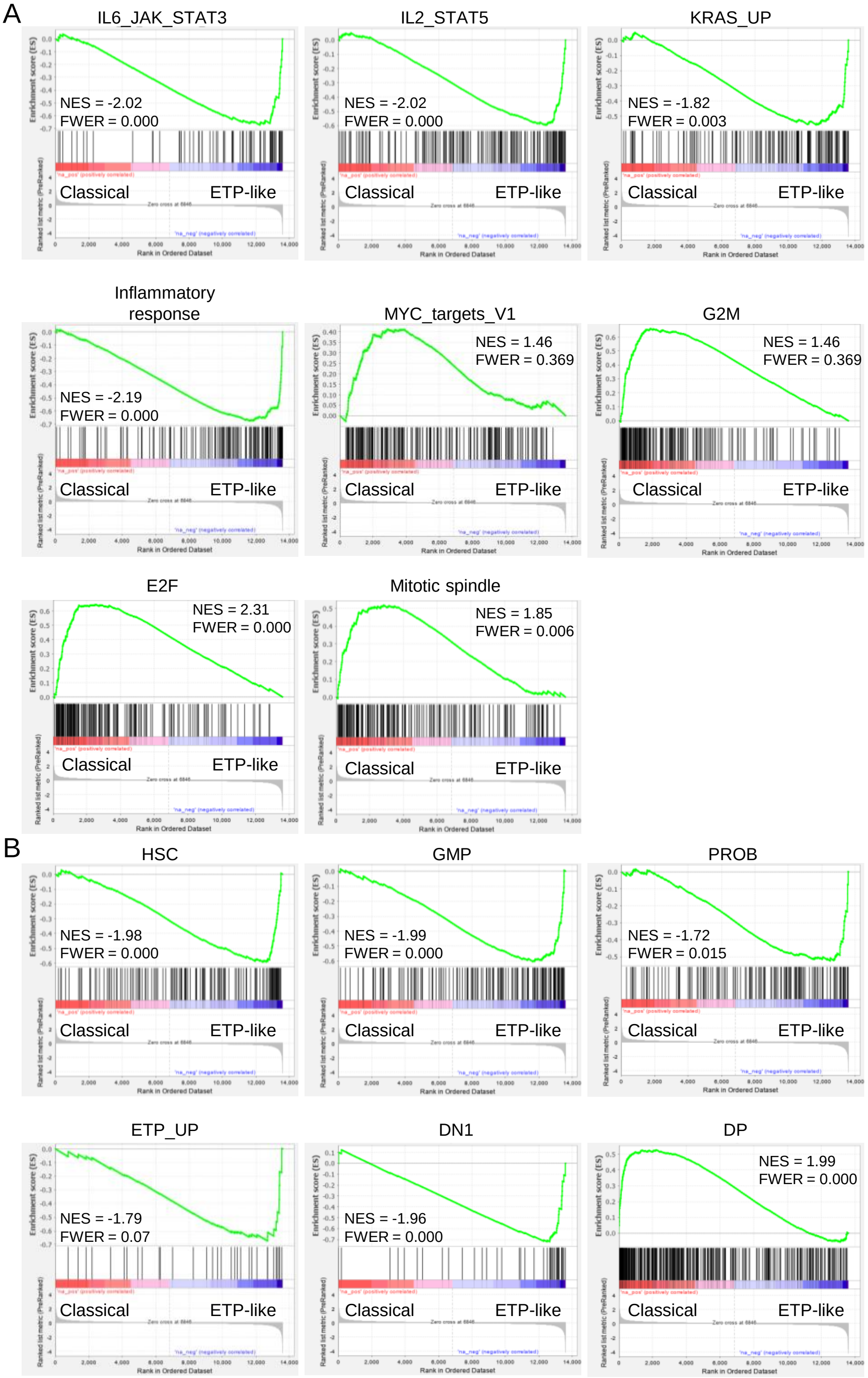


**Supplementary Figure 14. Transcriptome-based differentiation of classical and ETP-like T-ALL subgroups. A,** Gene set enrichment analysis (GSEA) using hallmark gene sets (h.all.v7.2.symbols.gmt) for pathway analysis between classical and ETP-like subgroups. **B,** GSEA using hematopoietic gene signatures (obtained from Laurenti et al. and Novershtern et al). Normalized enrichment score (NES) and FWER values are shown. Related to Figure 6, Table S15.

Figure S15

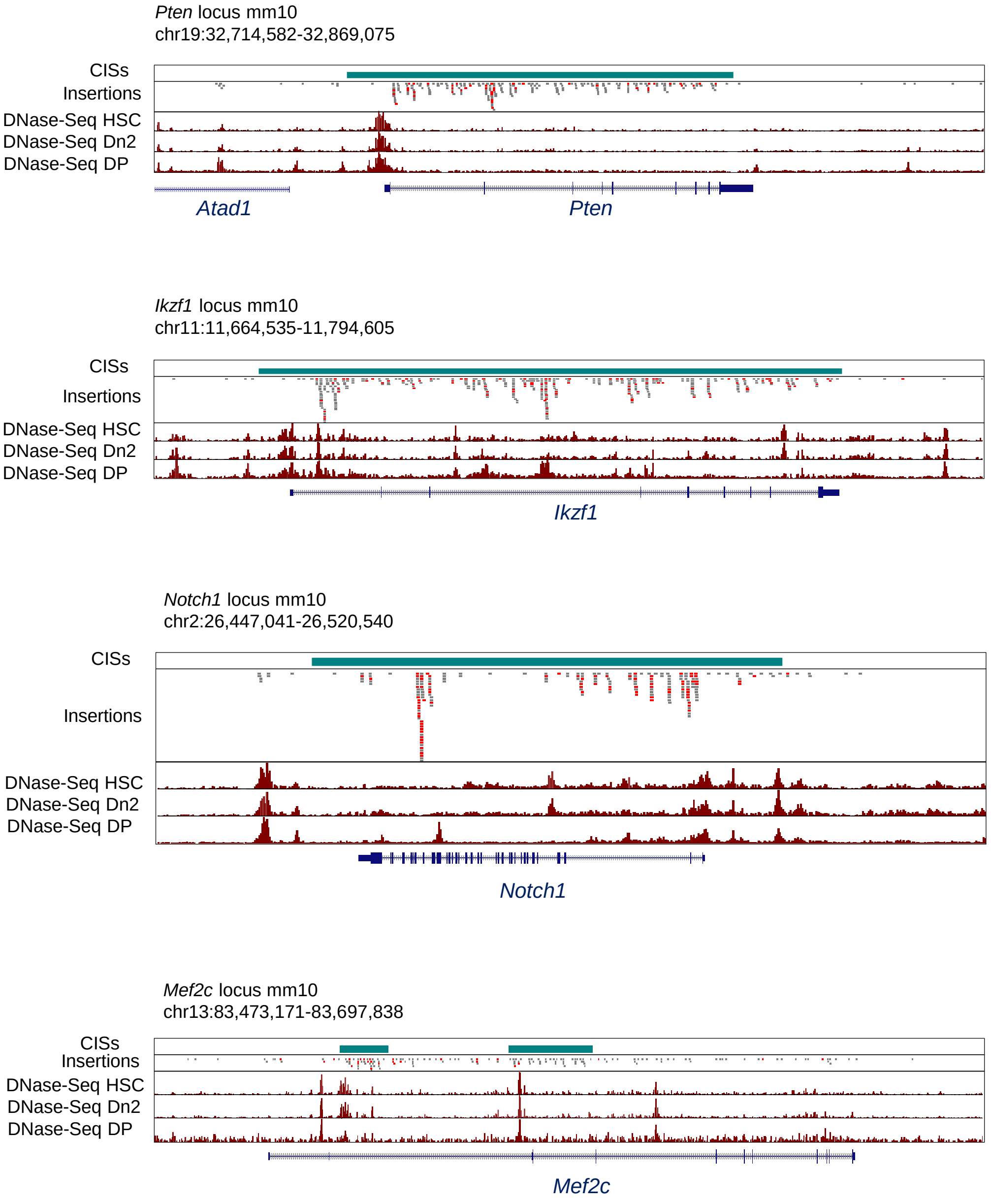


**Supplementary Figure 15. Chromatin accessibility of major driver genes identified in the screen.** UCSC Genome Browser image showing common insertion sites (CISs) and transposon insertions from *Rosa26PB/+;ATP2* mice. DNase-Seq data from different stages of T cell evolution are shown (publicly available data as listed in Supplementary Table 5). Protein-coding transcripts from the GENCODE gene annotation track are shown. High coverage insertions are shown in red, other insertions in grey. All T-ALL driver genes identified in the screen show a similar accessibility at their promoter in the course of T cell development. Insertion peaks are not primarily overlapping with peaks of open chromatin but show a broad distribution also in regions with less DNAse-Seq signal. Related to Figure 6.

Figure S16

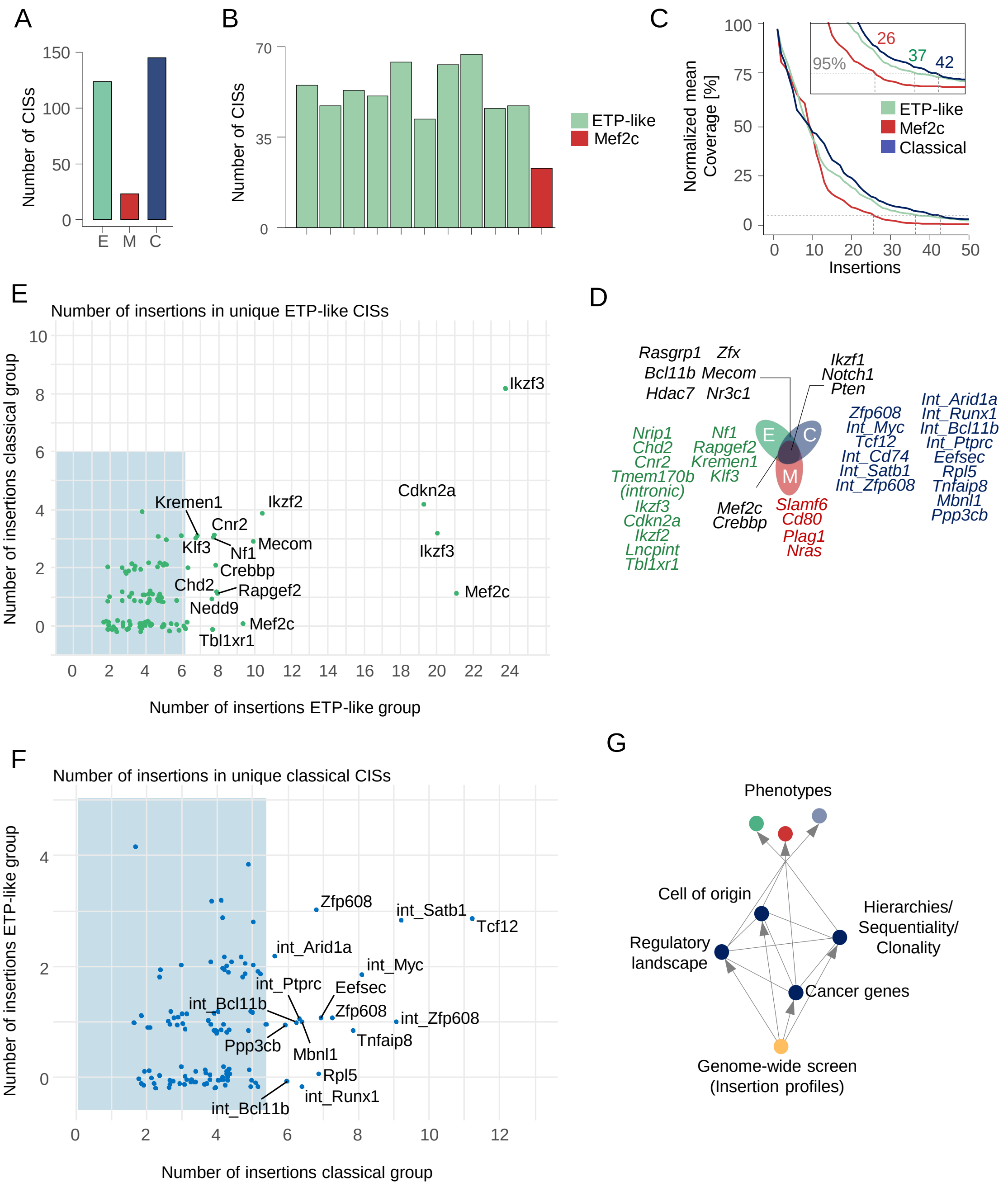


**Supplementary Figure 16. Differences in common insertions sites between subgroups. A,** Number of CISs identified in each T-ALL subgroup. Only CISs with at least two contributing insertions are considered. Number of unique insertions for each sample from ETP-like (n = 14), Mef2c-driven (n = 7) and classical (n = 8) T-ALL (*P < 0.05, Student's t test). **B,** Permutation test to identify number of CISs in ETP-like groups with same sample size as Mef2c-driven group (n = 7). Ten random selections of 7 samples of the ETP-like group (n = 14) were submitted to CIS analysis. CIS numbers are shown for the 10 permutation ETP samples and the Mef2c group. **C,** Normalized mean coverage of all samples in one subgroup is shown. Inlet depicts number of insertions contributing to 95% of the tumor. The Mef2c-driven subgroup is characterized by a lower number of insertions compared to the other groups. **D,** Shared and unique "top" CISs identified in the analyzed subgroups. Regulatory CISs are labelled with 'int' and 'intronic' for intergenic and intronic regulatory elements, respectively, and the putative target gene is reported. **E-F,** Comparison of CISs in ETP-like and classical subtype. Unique common insertion sites were selected by removing all CIS regions overlapping with a CIS region in the other group. Number of insertions in unique CIS regions were counted. Grey box marks region with CISs of low confidence. **E,** CIS regions unique to the ETP-like group are shown. CISs with more than 6 insertions are labelled with the respective gene overlapping the CIS region. **F,** CIS regions unique to the classical group are shown. CISs with more than 5 insertions are labelled with the respective gene overlapping the CIS region. Intergenic CISs are labelled with 'int' and the putative target gene. **G,** Integrative mapping of cellular and molecular determinants shaping tumor subtype evolution. Scheme showing the different layers of information that can be extracted from transposon insertion profiles to characterize the determinants of oncogenesis. The possibility to capture these determinants and their interplay as well as the related phenotypic outcomes in our model enables construction of multidimensional evolutionary maps. Related to Figure 6, Table S16-18.
